# Spermidine is essential for fasting-mediated autophagy and longevity

**DOI:** 10.1038/s41556-024-01468-x

**Published:** 2024-08-08

**Authors:** Sebastian J. Hofer, Ioanna Daskalaki, Martina Bergmann, Jasna Friščić, Andreas Zimmermann, Melanie I. Mueller, Mahmoud Abdellatif, Raffaele Nicastro, Sarah Masser, Sylvère Durand, Alexander Nartey, Mara Waltenstorfer, Sarah Enzenhofer, Isabella Faimann, Verena Gschiel, Thomas Bajaj, Christine Niemeyer, Ilias Gkikas, Lukas Pein, Giulia Cerrato, Hui Pan, YongTian Liang, Jelena Tadic, Andrea Jerkovic, Fanny Aprahamian, Christine E. Robbins, Nitharsshini Nirmalathasan, Hansjörg Habisch, Elisabeth Annerer, Frederik Dethloff, Michael Stumpe, Franziska Grundler, Françoise Wilhelmi de Toledo, Daniel E. Heinz, Daniela A. Koppold, Anika Rajput Khokhar, Andreas Michalsen, Norbert J. Tripolt, Harald Sourij, Thomas R. Pieber, Rafael de Cabo, Mark A. McCormick, Christoph Magnes, Oliver Kepp, Joern Dengjel, Stephan J. Sigrist, Nils C. Gassen, Simon Sedej, Tobias Madl, Claudio De Virgilio, Ulrich Stelzl, Markus H. Hoffmann, Tobias Eisenberg, Nektarios Tavernarakis, Guido Kroemer, Frank Madeo

**Affiliations:** 1 Institute of Molecular Biosciences, NAWI Graz, University of Graz, Graz, Austria; 2 Field of Excellence BioHealth, University of Graz, Graz, Austria; 3 BioTechMed Graz, Graz, Austria; 4 Centre de Recherche des Cordeliers, Équipe Labellisée par la Ligue Contre le Cancer, Université de Paris Cité, Sorbonne Université, Inserm U1138, Institut Universitaire de France, Paris, France; 5 Metabolomics and Cell Biology Platforms, Gustave Roussy Cancer Center, Université Paris Saclay, Villejuif, France; 6 Institute of Molecular Biology and Biotechnology, Foundation for Research and Technology – Hellas, Heraklion, Greece; 7 Department of Biology, School of Sciences and Engineering, University of Crete, Heraklion, Greece; 8 Department of Dermatology, Allergy and Venerology, University of Lübeck, Lübeck, Germany; 9 Institute for Systemic Inflammation Research, University of Lübeck, Lübeck, Germany; 10 Division of Cardiology, Medical University of Graz, Graz, Austria; 11 Department of Biology, University of Fribourg, Fribourg, Switzerland; 12 Institute of Pharmaceutical Sciences, Pharmaceutical Chemistry, University of Graz, Graz, Austria; 13 Neurohomeostasis Research Group, Department of Psychiatry and Psychotherapy, University Hospital Bonn, Bonn, Germany; 14 Institute for Biology and Genetics, Freie Universität Berlin, Berlin, Germany; 15 Cluster of Excellence, NeuroCure, Berlin, Germany; 16 Department of Biochemistry and Molecular Biology, University of New Mexico Health Sciences Center, Albuquerque, NM USA; 17 Research Unit Integrative Structural Biology, Otto Loewi Research Center, Medicinal Chemistry, Medical University of Graz, Graz, Austria; 18 Max Planck Institute for Biology of Ageing, Cologne, Germany; 19 Buchinger Wilhelmi Clinic, Überlingen, Germany; 20 Institute of Social Medicine, Epidemiology and Health Economics, corporate member of Freie Universität Berlin and Humboldt-Universität, Charité-Universitätsmedizin, Berlin, Germany; 21 Department of Pediatrics, Division of Oncology and Hematology, Charité – Universitätsmedizin Berlin, Corporate Member of Freie Universität Berlin and Humboldt-Universität zu Berlin, Berlin, Germany; 22 Department of Internal Medicine and Nature-based Therapies, Immanuel Hospital Berlin, Berlin, Germany; 23 Department of Dermatology, Venereology and Allergology, Charité – Universitätsmedizin Berlin, corporate member of Freie Universität Berlin and Humboldt-Universität zu Berlin, Berlin, Germany; 24 Interdisciplinary Metabolic Medicine Trials Unit, Division of Endocrinology and Diabetology, Medical University of Graz, Graz, Austria; 25 Division of Endocrinology and Diabetology, Department of Internal Medicine, Medical University of Graz, Graz, Austria; 26 HEALTH - Institute for Biomedical Research and Technologies, Joanneum Research Forschungsgesellschaft, Graz, Austria; 27 Experimental Gerontology Section, Translational Gerontology Branch, National Institute on Aging, National Institutes of Health, Baltimore, MD USA; 28 Institute of Physiology, Faculty of Medicine, University of Maribor, Maribor, Slovenia; 29 Division of Basic Sciences, School of Medicine, University of Crete, Heraklion, Greece; 30 Institut du Cancer Paris CARPEM, Department of Biology, Hôpital Européen Georges Pompidou, AP-HP, Paris, France

**Keywords:** Molecular biology, Macroautophagy, Metabolomics, Autophagy

## Abstract

Caloric restriction and intermittent fasting prolong the lifespan and healthspan of model organisms and improve human health. The natural polyamine spermidine has been similarly linked to autophagy enhancement, geroprotection and reduced incidence of cardiovascular and neurodegenerative diseases across species borders. Here, we asked whether the cellular and physiological consequences of caloric restriction and fasting depend on polyamine metabolism. We report that spermidine levels increased upon distinct regimens of fasting or caloric restriction in yeast, flies, mice and human volunteers. Genetic or pharmacological blockade of endogenous spermidine synthesis reduced fasting-induced autophagy in yeast, nematodes and human cells. Furthermore, perturbing the polyamine pathway in vivo abrogated the lifespan- and healthspan-extending effects, as well as the cardioprotective and anti-arthritic consequences of fasting. Mechanistically, spermidine mediated these effects via autophagy induction and hypusination of the translation regulator eIF5A. In summary, the polyamine–hypusination axis emerges as a phylogenetically conserved metabolic control hub for fasting-mediated autophagy enhancement and longevity.

## Abstract (web summary)

Hofer et al. show that fasting promotes the synthesis of spermidine, which stimulates eIF5A hypusination to induce autophagy and increase lifespan in various species in a conserved manner.

## Main

Continuous caloric restriction (CR) remains the gold standard for extending the lifespan and healthspan of model organisms^1,2^. Recently, intermittent fasting (IF) interventions, often combined with CR, emerged as alternatives for clinical implementation^3^. However, to date, it remains uncertain whether IF offers health benefits due to the temporary cessation of caloric intake (without CR) or due to a net reduction of total calories de facto resulting in CR^4–6^. IF, like CR, delays hallmarks of aging in yeast, worms, insects and mice^3,7–9^. In humans, intermittent^7^ and long-term^10^ fasting, as well as continuous CR^11^, are associated with favourable effects on multiple health-relevant parameters that may share a common mechanistic basis. Strong evidence exists that macroautophagy (hereafter referred to as ‘autophagy’) mediates these effects^12^.

In mammals, an age-associated reduction in autophagic flux^13^ contributes to the accumulation of protein aggregates and dysfunctional organelles, failing pathogen elimination and exacerbated inflammation^14^. Genetic autophagy inhibition accelerates aging processes in mice^13^ and loss-of-function mutations of genes that regulate or execute autophagy have been causally linked to cardiovascular, infectious, neurodegenerative, metabolic, musculoskeletal, ocular and pulmonary diseases, many of which resemble premature aging^15–18^. Conversely, genetic autophagy stimulation promotes healthspan and lifespan in model organisms, including flies^19^ and mice^20,21^. Besides nutritional interventions, administering the natural polyamine spermidine (SPD) to yeast, worms, flies and mice is another strategy to extend the lifespan in an autophagy-dependent fashion^22–26^. Moreover, SPD restores autophagic flux in circulating lymphocytes from aged humans^25,27^, coinciding with the observation that increased dietary SPD uptake is associated with reduced overall mortality in human populations^28^.

Hence, fasting, CR and SPD extend the lifespan of model organisms and activate phylogenetically conserved, autophagy-dependent geroprotection. Intrigued by these premises, we investigated whether the geroprotective effects of IF might be connected to, or depend on, SPD.

## Results

### Fasting elevates spermidine levels

**Fig. 1:**
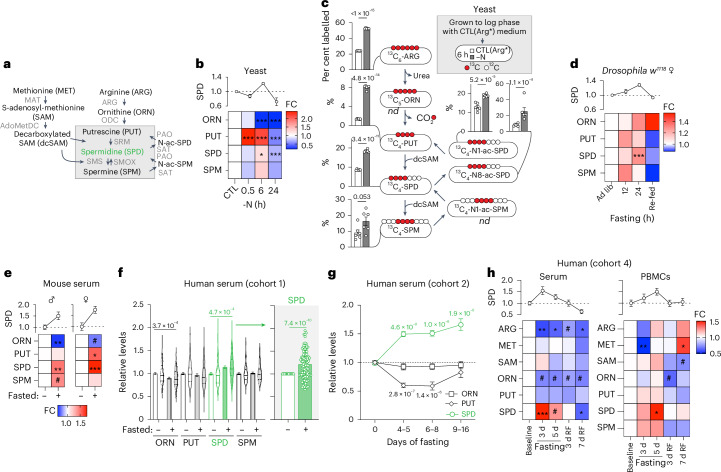
Fasting induces polyamine synthesis in various species. **a**, Schematic overview of the polyamine pathway and adjacent metabolites. AdoMetDC, adenosylmethionine decarboxylase; ARG, arginase; MAT, methionine adenosyltransferase; ODC, ornithine decarboxylase; SAT, SPD/SPM acetyltransferase, SRM, spermidine synthase; SMOX, spermine oxidase; SMS, spermine synthase; PAO, polyamine oxidase. **b**, Polyamine levels of WT BY4741 yeast shifted to nitrogen-deprived medium (−N) for the indicated times. Data are normalized to the mean of the control (CTL) condition at every time point. Note that the statistics were performed together with additional groups as indicated in Supplementary Fig. 1a. *n* = 6 biologically independent samples (yeast cultures). **c**, WT BY4741 yeast cells were pre-labelled with ^13^C_6_-arginine [CTL(Arg*)] and shifted to CTL(Arg*) or −N medium for 6 h. MS-based analysis of labelled products revealed a uniform increase in percentage of labelled polyamines in N-starved cells. nd, not detected. *n* = 6 biologically independent samples (yeast cultures). **d**, Polyamine levels of young female *w*^*1118*^ flies fasted for 12 or 24 h (starting at 20:00 upon lights turned off). Data are normalized to the ad libitum (ad lib) group at every time point. Re-fed, 12 h re-feeding after 24 h fasting. *n* = 5 (re-fed, 24 h ad lib ORN), 6 (24 h ad lib PUT, SPD and SPM), 7 (rest) biologically independent samples (groups of flies). **e**, Serum polyamine levels of young male and female C57BL/6 mice fasted or kept ad lib for 14–16 h overnight, starting at 16:00–17:00). *n* = 5 (male), 8 (female) mice. **f**, Relative polyamine levels in human serum from cohort 1 after fasting (9 (7–13) days) depicted as mean with s.e.m. and violin plots, showing median and quartiles as lines. The extra panel depicts the individual increase in SPD levels for every volunteer. *n* = 104 (PUT), 109 (SPD baseline), 100 (SPM baseline), 105 (SPD fasted), 94 (SPM fasted) volunteers. **g**, Relative polyamine levels in human serum from cohort 2 after increasing numbers of fasting days. *n* = 61 (baseline PUT), 62 (baseline ORN and SPD), 22 (4–5 d PUT and SPD), 25 (4–5 d ORN, 6–8 d ORN), 19 (6–8 d PUT), 20 (6–8 d SPD), 9 (9–16 d PUT and SPD), 13 (9–16 d ORN) volunteers. **h**, Relative polyamine and precursor levels in human serum and PBMCs from cohort 4 after increasing numbers of fasting days. BL, baseline, RF = days 3 or 7 after re-introduction of food. *N* (serum) = 7 (BL SAM), 11 (3 d SAM), 12 (7d RF SAM), 13 (5d SAM), 14 (BL PUT and SPD), 15 (BL ARG and MET; 5 d ARG; 3d RF SAM), 16 (BL ORN; 5 d MET, ORN, PUT and SPD), 17 (3 d ARG, MET, ORN, PUT and SPD; 7 d RF ARG, MET, ORN, PUT and SPD), 18 (3 d RF ARG, MET, ORN, PUT and SPD) volunteers. *N* (PBMCs) = 6 (5 d ORN), 7 (5 d rest), 9 (BL SPD), 10 (BL rest), 11 (3 d RF SPD) 12 (3 d SPD and SPM; 3 d RF rest), 13 (3 d rest; 7 d RF) volunteers. Statistics used were two-way analysis of variance (ANOVA) with Holm-Šídák’s multiple comparisons test (**b**,**d**,**f**,**h**) and two-tailed Student’s *t*-tests (**c**). For every analyte (**e**) two-way ANOVA with false discovery rate (FDR) correction (two-stage step-up method by Benjamini, Krieger and Yekutieli, *Q* = 0.05) together with data depicted in Extended Data Fig. 1f–i (male) and Extended Data Fig. 1k–n (female). Wilcoxon matched-pairs signed rank test (**f**). Kruskal–Wallis test with Dunn’s multiple comparison test (**g**). FC, fold change to control. Heatmaps show means. Bar and line graphs show mean ± s.e.m. *P < 0.05, **P < 0.01, ***P < 0.001, ^#^*P* < 0.2. Source numerical data are available in source data. Source data

To investigate polyamine metabolism during IF, we subjected four different organisms to acute fasting stimuli. Mass spectrometry (MS)-based quantification of SPD, the primary biologically active polyamine and its precursors ornithine (ORN), putrescine (PUT), as well as its metabolite spermine (SPM) (Fig. 1a) revealed a uniform increase in polyamine content upon fasting across various species.

**Extended Data Fig. 1:**
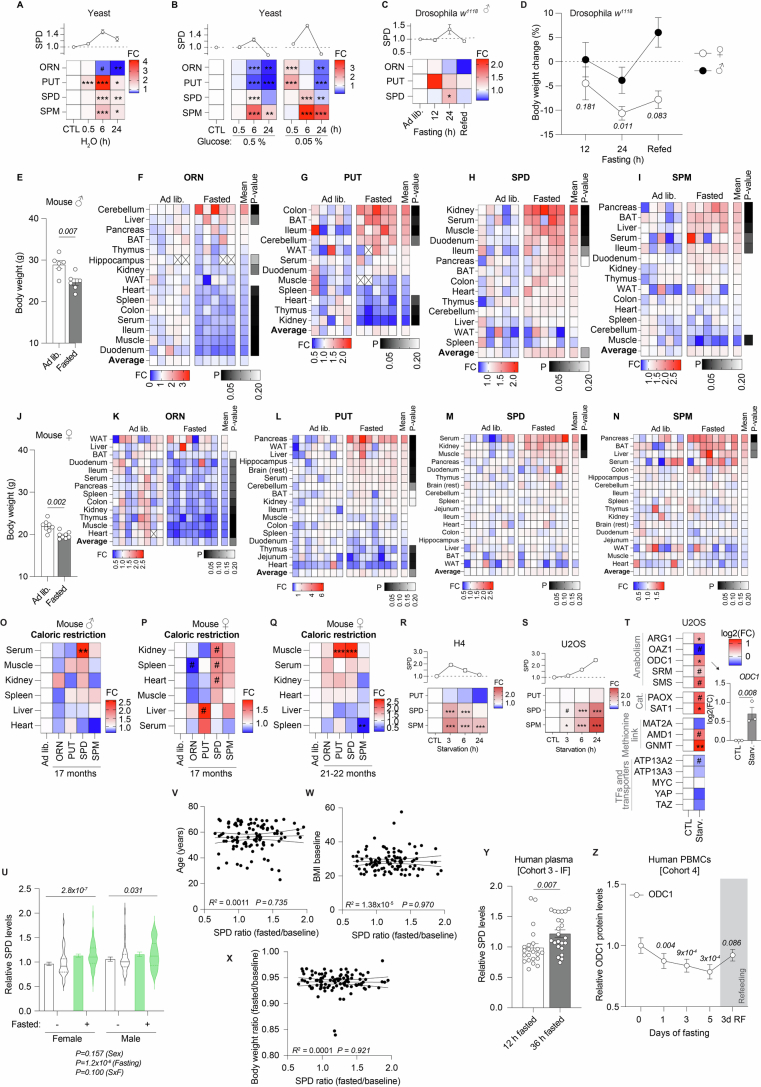
Changes in polyamine levels in starving organisms. **(a)** Polyamine levels of WT BY4741 yeast shifted to water for the indicated times. Data normalized to the mean of the control group at every time point. N = 5 biologically independent samples (yeast cultures). **(b)** Polyamine levels of glucose-restricted WT BY4741 after the indicated times. Data normalized to the mean of the control group at every time point. N = 5 biologically independent samples (yeast cultures). **(c)** Polyamine levels of young male *w*^*1118*^ flies fasted for 12 or 24 hours (starting at 20:00). Data normalized to the ad libitum group at every time point. Refed = 12 hours refeeding after 24 hours of fasting. N = 7 biologically independent samples (groups of flies). **(d)** Changes in body weight of young female and male *w*^*1118*^ flies fasted or kept ad lib for the indicated times, starting at 20:00. Data normalized to each ad lib group. *P* values against each sex’s ad lib group. Refed = 12 hours refeeding after 24 hours of fasting. N = 5(Refed female fasted), 6(24 h female ad lib; Refed female ad lib; Refed male ad lib), 7(12 h, 24 h female fasted; 12 h, 24 h male; Refed male fasted) biologically independent samples (groups of flies). **(e)** Changes in body weight of young male C57BL/6 mice fasted or kept ad lib for 14-16 hours overnight. N = 6 mice. **(f-i)** Relative polyamine levels of young male C57BL/6 mice fasted or kept ad lib for 14-16 hours overnight. BAT = brown adipose tissue, WAT = white adipose tissue. FC = fold change to means of ad lib N = 3(ORN Hippocampus; PUT Muscle fasted), 4(PUT WAT fasted), 5(rest) mice. **(j)** Changes in body weight of young female C57BL/6 mice fasted or kept ad lib for 14-16 hours overnight. N = 8 mice. **(k-n)** Relative polyamine levels of young female C57BL/6 mice fasted or kept ad lib for 14-16 hours overnight. BAT = brown adipose tissue, WAT = white adipose tissue. FC = fold change to means of ad lib. N = 7(ORN Heart ad lib), 8(rest) mice. **(o-q)** Relative polyamine levels of male and female C57BL/6 mice kept ad lib or calorie-restricted (30 %), starting at 9 months of age, until the age of 17 or 21 months. **(o)** N = 4(Fasted Spleen), 6(Fasted Kidney, Serum, Heart; SPM Fasted Liver), 7(Ad lib Spleen; Fasted Muscle, Liver rest), 8(Ad lib Kidney, Liver), 10(Ad lib Serum), 11(Ad lib Heart, Muscle) mice. **(p)** N = 3(CR Serum SPM), 4(CR Liver, Serum rest), 5(CR Kidney, Spleen), 6(Ad lib Serum; CR Heart, Muscle), 7(), 8(Ad lib Liver, Muscle), 9(Ad lib Kidney, Heart, Spleen) mice. **(q)**N = 3(Ad lib Liver), 4(Ad lib Kidney, Spleen), 6(Ad lib Serum, Heart, Muscle; CR Kidney, Spleen), 7(CR Heart, Muscle), 8(CR Liver, Serum) mice. **(r-s)** Polyamine levels in HBSS-starved human U2OS and H4 cells. N = 4 biologically independent samples. **(t)** Relative mRNA expression levels of polyamine-relevant genes in 6 hours starved U2OS cells. N = 3(ARG1, OAZ1, ODC1, SAT1, ATP13A2, ATP13A3, MYC, YAP, TAZ), 4(SRM, SMS, PAOX, MAT2A, AMD1, GNMT) biologically independent samples. **(u)** Serum SPD levels after fasting in cohort 1, stratified by sex, depicted as mean with S.E.M and violin plots, showing median and quartiles as lines. N = 67(female), 38(male). **(v-x)** Serum SPD levels after fasting in cohort 1 do not correlate with age, pre-fasting baseline body mass index (BMI) or body weight loss (body weight ratio). N = 99(Body weight ratio), 102(BMI), 105(Age) volunteers. **(y)** Relative SPD levels in human plasma from cohort 3 during IF. N = 22(12 h), 23(36 h) volunteers. **(z)** ODC1 protein levels in human PBMCs after increasing fasting times, measured by capillary electrophoresis. Refeeding = day 3 after re-introduction of food. N = 15(5 d), 16(3d RF), 17(rest) volunteers. Statistics: [A-D,R-S,U,Z] Two-way ANOVA with Holm-Šídák’s multiple comparisons test. [E,J,T] Two-tailed Student’s *t*-tests. [F-I, K-Q] Two-way ANOVA with FDR correction (Two-stage step-up method by Benjamini, Krieger and Yekutieli, Q = 0.05). [Y] Two-tailed Student’s *t*-test. [V-X] Simple linear regression analysis. Bar and line graphs show the mean ± s.e.m. Heatmaps show means. * *P* < 0.05, ** *P* < 0.01, *** *P* < 0.001, # *P* < 0.2. Source numerical data are available in source data. Source data

Starving *Saccharomyces* *cerevisiae* (yeast) cells in water increased the levels of SPD and SPM (Extended Data Fig. 1a), similar to glucose restriction (Extended Data Fig. 1b), while ORN generally decreased. Nitrogen starvation (−N), a classical autophagy-inducing intervention^29,30^, elicited a fast and transient increase in polyamines, mainly PUT, accompanied by a drastic decrease in the precursor ORN (Fig. 1b). To shed light on the dynamics under −N, we studied metabolic flux in yeast using ^13^C_6_-labelled arginine (ARG). We found that the cellular levels of ARG-derived polyamines after 6 h of nitrogen deprivation were higher than in N-containing control medium (Fig. 1c). Thus, despite the elimination of extracellular nitrogen, polyamine flux remained active, favouring the utilization of residual ARG molecules for polyamine synthesis.

SPD increased in female and male *Drosophila* *melanogaster* (fruit fly) fasted for 24 h (Fig. 1d and Extended Data Fig. 1c), which caused a body weight loss of 10% and 5%, respectively (Extended Data Fig. 1d). This SPD increase was reversed by 12 h re-feeding (Fig. 1d and Extended Data Fig. 1c). Similarly, young male and female C57BL6/J mice fasted for 14–16 h, which caused significant weight loss (Extended Data Fig. 1e,j), had higher SPD levels (but not PUT nor SPM) in the serum than their ad libitum-fed controls, whereas ORN content was reduced (Fig. 1e). Moreover, the abundance of ORN and polyamines changed significantly in several organs of acutely fasted mice in a tissue-specific manner (Extended Data Fig. 1f–i,k–n), favouring an increase of SPD in multiple tissues. We next asked whether these alterations in polyamine content would also occur during long-term CR, starting at 9 months of age^31^. We found increased serum SPD at 17 months of age in male, but not female, mice (Extended Data Fig. 1o,p). At later time points (21 months), female CR mice also showed significantly elevated PUT and SPD levels in skeletal muscle (Extended Data Fig. 1q).

Furthermore, starvation elevated SPD and SPM levels uniformly in human U2OS osteosarcoma and H4 glioblastoma cells (Extended Data Fig. 1r,s). Accordingly, in nutrient-depleted U2OS cells, the expression of arginase (*ARG1*), ornithine decarboxylase (*ODC1)*, spermidine/spermine N1-acetyltransferase 1 (*SAT1*) and glycine *N*-methyltransferase (*GNMT*) increased compared with cells cultured in control medium, whereas the polyamine-associated transcription factors *MYC* and *YAP/TAZ*, as well as two recently identified polyamine transporters, ATPase cation transporting 13A2/13A3 (*ATP13A2/3*), were unaffected (Extended Data Fig. 1t).

In human volunteers, long-term therapeutic fasting with a daily caloric intake of approximately 250 kcal (Supplementary Table 1) under clinical supervision for 7–13 days, SPD levels (but not PUT nor SPM) significantly increased in the serum (Fig. 1f). This increase in SPD content was similarly found in men and women (Extended Data Fig. 1u), and was independent of age and body mass index (BMI) before the intervention or body weight loss (Extended Data Fig. 1v–x).

We analysed an independent cohort of volunteers fasting for variable periods (Supplementary Table 1; cohort 2). SPD levels increased by ~50% after 4–5 days and remained elevated during long-term fasting (Fig. 1g). In a third cohort (Supplementary Table 1), we analysed plasma SPD of individuals who voluntarily followed an IF routine (12-h eating periods followed by 36-h zero-calorie periods for several months). Again, we observed an elevation in SPD levels (Extended Data Fig. 1y). Finally, SPD increased in serum and peripheral blood mononuclear cells (PBMCs) of a separate, fourth cohort of volunteers during fasting and reverted to baseline levels after re-feeding (Fig. 1h). Notably, in these PBMCs, ODC1 protein levels decreased during fasting (Extended Data Fig. 1z), suggesting that elevated SPD levels either caused a feedback repression of ODC1 or, at least in these cells, might stem from increased uptake rather than intracellular synthesis.

In conclusion, nutrient starvation (yeast and human cell lines), overnight fasting (flies, mice and humans), long-term CR (mice) or long-term fasting (humans) induced SPD elevation.

### Polyamine synthesis is required for efficient metabolic remodelling and TORC1 inhibition during fasting

**Fig. 2:**
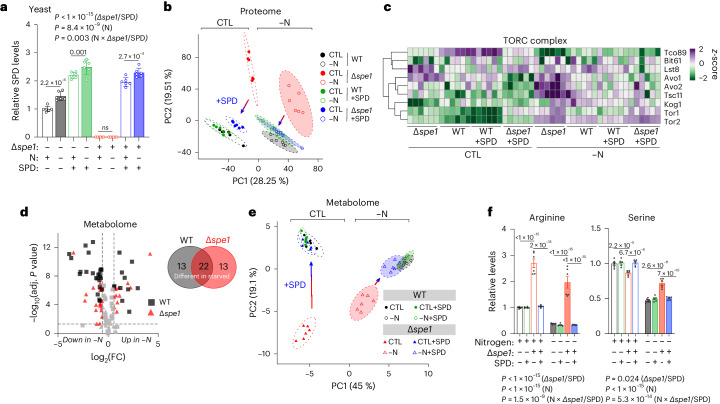
Spermidine synthesis is required for the cellular adaptation to starvation. **a**, Relative SPD levels of WT and ∆*spe1* yeast cells after SPD treatment (100 µM) and N starvation. *n* = 6 biologically independent samples (yeast cultures). **b**, PCA depicting the proteome change in WT and Δ*spe*1 cells under specified condition treatments following a 6-h culture in control or −N medium with or without 100 µM SPD. PCA was performed on a singular-value decomposition of centred and scaled protein groups (*n* = 4,684) displays a comparison between PC1 and PC2, along with the representation of a 95% confidence interval for each group. *n* = 6 biologically independent samples (yeast cultures). **c**, Differential expression (*z*-score) of proteins involved in the TORC complex, from the proteome analysis shown in **b**. *n* = 6 biologically independent samples (yeast cultures). **d**, Volcano plot showing significantly different intracellular metabolites in WT or ∆*spe1* after 6 h −N compared with control conditions. Venn diagram showing exclusive and overlapping significantly regulated metabolites. FDR-corrected *P* value < 0.05, FC > 1.5. *n* = 4 biologically independent samples (yeast cultures). **e**, Metabolomic disturbances in ∆*spe1* cells are rescued by SPD (100 µM) supplementation. The PCA displays a comparison between PC1 and PC2, along with the representation of a 95% confidence interval for each group. *n* = 6 biologically independent samples (yeast cultures). **f**, SPD (100 µM) corrects ∆*spe1*-associated amino acid disturbances. Relative arginine and serine levels from metabolomics analysis shown in **e**. *n* = 6 biologically independent samples (yeast cultures). Statistics were two-way ANOVA with Holm-Šídák’s multiple comparisons test (**a**,**f**) and two-tailed Student’s *t*-tests with FDR correction (**d**). Bar graphs show the mean ± s.e.m. Source numerical data are available in source data. Source data

Next, we investigated the cellular consequences of impaired polyamine anabolism on acute fasting responses. We generated yeast lacking the rate-limiting enzyme ornithine decarboxylase (ODC1; yeast Spe1), which are characterized by polyamine depletion (Supplementary Fig. 1a). This strain (∆*spe1*) showed no elevation of polyamines upon starvation, and SPD supplementation (100 µM) fully replenished the intracellular SPD pool (Fig. 2a). We subjected ∆*spe1* cells with and without SPD to proteomic analyses after 6 h nitrogen starvation (Supplementary Fig. 1b). Principal-component analysis (PCA) of the proteome revealed a clear distinction between the genotypes and that SPD could revert ∆*spe1*-associated global differences, whereas it did not affect the wild-type (WT) proteome (Fig. 2b). Mapping the identified proteins to Kyoto Encyclopedia of Genes and Genomes (KEGG) terms, we found several pathways that have been implicated in the starvation response dysregulated in ∆*spe1* (Supplementary Fig. 1c). This included, for example, the metabolism of several amino acids (including ARG), lipids and fatty acids, as well as energy-relevant pathways (tricarboxylic acid (TCA) cycle and oxidative phosphorylation). Notably, we also found a disturbed starvation response of the proteostasis-associated pathways autophagy and TORC1/2, the yeast homologues of mechanistic target of rapamycin complex 1/2 (mTORC1/2), in ∆*spe1* versus WT cells (Fig. 2c and Supplementary Fig. 1c).

The prominent dysregulation in metabolic pathways was supported by unbiased metabolomic profiling by nuclear magnetic resonance (NMR) spectroscopy, revealing substantial differences in the intracellular metabolomes after nitrogen deprivation (Supplementary Fig. 2). The metabolic disturbances affecting starved ∆*spe1* cells (Fig. 2d) confirmed findings from the proteome analysis, including increased citric acid and reduced levels of nicotinamide adenine dinucleotide (NAD^+^) and adenosine/guanosine-X-phosphate (AXP/GXP; where X stands for mono-, di- or triphosphate), suggesting a disrupted energy metabolism secondary to the loss of intracellular polyamine synthesis (Supplementary Fig. 2). Our analysis also indicated dysregulated amino acid homoeostasis, which is normally maintained by autophagy in starving yeast^32–34^.

**Extended Data Fig. 2:**
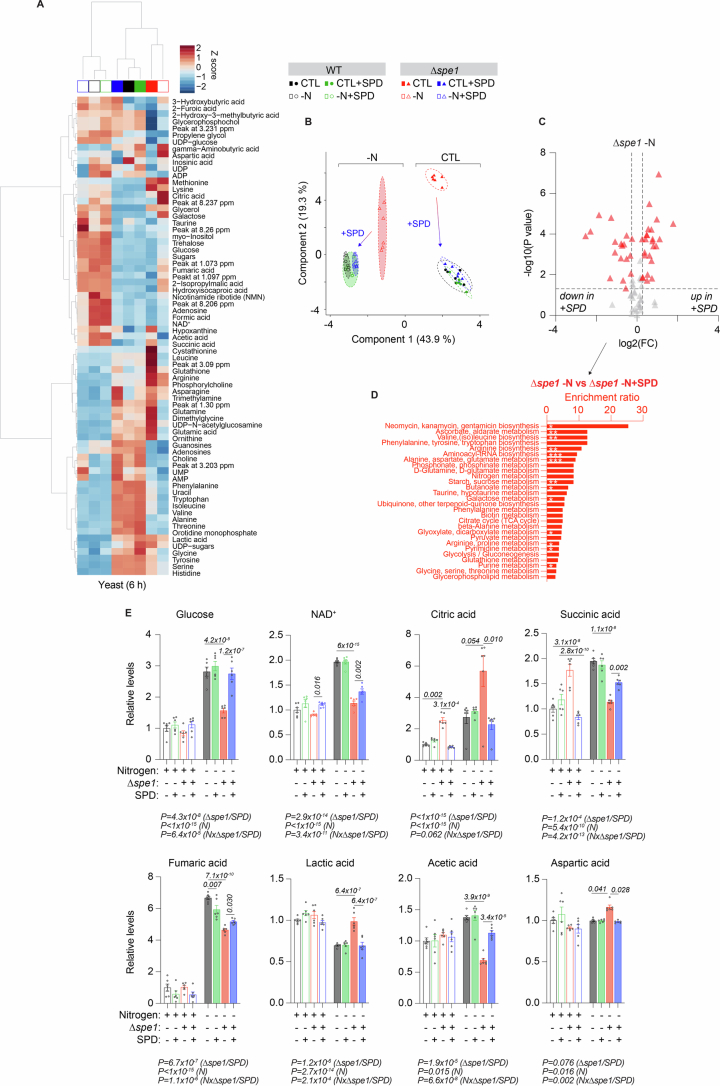
Spermidine supplementation corrects metabolome disturbances in ∆*spe1* yeast cells. **(a, b)** Heatmap (group means) and PCA of *S. cerevisiae* WT and ∆*spe1* metabolomes after 6 hours -N, with or without 100 µM SPD. Unassigned NMR signals are labelled according to their NMR chemical shift. N = 6 biologically independent samples (yeast cultures). **(c)** Volcano plot showing significantly different metabolites in ∆*spe1* after 6 hours -N, with or without 100 µM SPD. Two-tailed Student’s *t*-tests with FDR-corrected *P* values < 0.05, FC (fold change) >1.2. N = 6 biologically independent samples (yeast cultures). **(d)** Metabolite set enrichment analysis based on KEGG pathways of significantly different metabolites from [C] (raw *P*-values < 0.2). **(e)** Selected metabolites from [A], focusing on amino acid metabolism and the TCA cycle. N = 6 biologically independent samples (yeast cultures). Statistics: [E] Two-way ANOVA with Holm-Šídák’s multiple comparisons test. Bar graphs show the mean ± S.E.M. Asterisks indicate raw *P*-values. *<0.05, **<0.01, ***<0.001. Source numerical data are available in source data. Source data

Notably, exogenously supplemented SPD reversed the metabolic dysregulations in ∆*spe1* cells, both in control and nitrogen-starvation medium, whereas it hardly affected the general WT metabolomes (Fig. 2e and Extended Data Fig. 2a,b). This included normalized amino acid metabolism (Fig. 2f and Extended Data Fig. 2c,d), which has been critically linked to TOR and autophagy regulation (for example, for arginine^35^ and serine^33^) and metabolites central to energy metabolism (glucose, NAD^+^ and citric acid, among others) (Fig. 2f and Extended Data Fig. 2e). Overall, Spe1 was required for the metabolic switch from glycolysis to oxidative phosphorylation, a key event in the cellular adaptation to nitrogen starvation, which partly depends on functional autophagy^36^. For instance, glucose levels were less increased under −N, and the ratios of TCA cycle metabolites were heavily dysregulated (for example, citric acid to succinic acid) (Extended Data Fig. 2e).

**Fig. 3:**
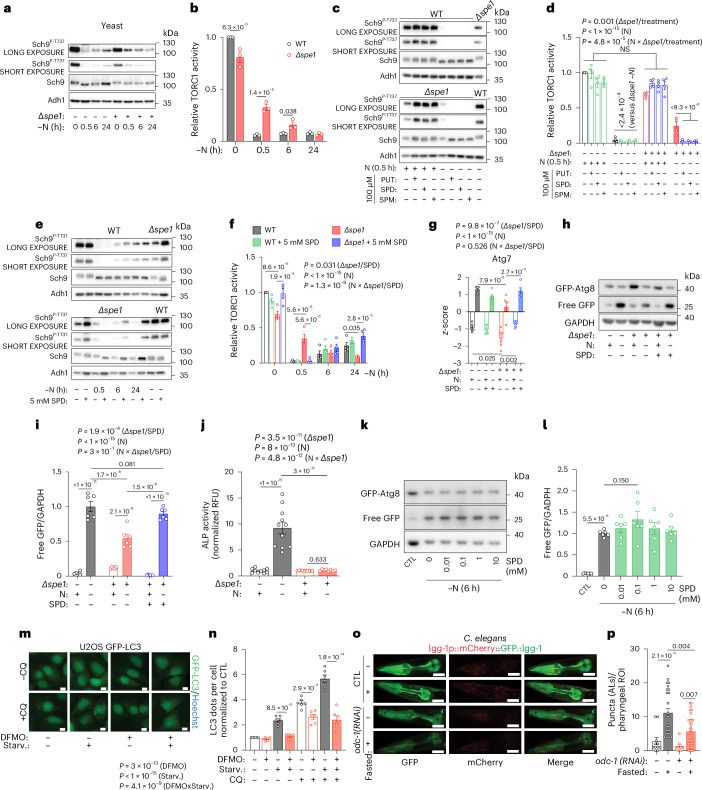
Autophagy induction is blunted by lack of polyamine synthesis. **a**, Decrease of TORC1 activity as inferred by Sch9-phosphorylation during -N in WT and ∆*spe1* cells. Representative immunoblot. **b**, Quantification of immunoblots as shown in **a**. *n* = 3 biologically independent samples (yeast cultures). **c**, Polyamine supplementation (100 µM) corrects the delayed decrease of TORC1 activity during −N in ∆*spe1* cells. Representative immunoblot. **d**, Quantification of immunoblots as shown in **c**. *n* = 4 biologically independent samples (yeast cultures). **e**, Supplementation of high SPD levels (5 mM) corrects the delayed decrease of TORC1 activity in ∆*spe1* cells but does not affect TORC1 activity in WT cells. Representative immunoblot. **f**, Quantification of immunoblots as shown in **e**. *n* = 4 biologically independent samples (yeast cultures). **g**, SPD supplementation (100 µM) corrects decreased Atg7 protein levels ∆*spe1* cells in both control and −N medium, as detected in proteome analysis shown in Fig. 2b. *n* = 6 biologically independent samples (yeast cultures). **h**, Representative immunoblots of yeast WT and ∆s*pe1* GFP-Atg8 cells after 6 h −N with and without 100 µM SPD, assessed for GFP and GAPDH. **i**, Quantifications of **h**. *n* = 6 biologically independent samples (yeast cultures). **j**, ALP activity (RFU per µg) from Pho8∆N60 assay normalized to each CTL group after 6 h −N. *n* = 10 (WT CTL, ∆*spe1* CTL), 11 (WT -N), 8 (∆*spe1* −N) biologically independent samples (yeast cultures). **k**, Representative immunoblots of yeast WT and ∆s*pe1* GFP-Atg8 after 6 h −N, with or without ascending concentrations of SPD, assessed for GFP and GAPDH. **l**, Quantifications of **k**. *n* = 6 biologically independent samples (yeast cultures). **m**, Representative images of human U2OS GFP-LC3 cells starved for 6 h in Hanks’ balanced salt solution (HBSS) (with or without chloroquine (CQ) for 3 h before fixation) after 3 days of 100 µM DFMO treatment. For quantifications see also Extended Data Fig. 5c. Scale bar, 10 µm. **n**, Quantification of cytosolic GFP-LC3 dots from **l**, normalized to the average number of GFP-LC3 dots in the control condition. *n* = 6 biologically independent experiments. **o**, Representative images of the head region of young control and *odc-1(RNAi) C.* *elegans* MAH215 (sqIs11 [lgg-1p::mCherry::GFP::lgg-1 + rol-6]) (LGG-1 is the *C.* *elegans* orthologue of LC3) fasted for two days. Autolysosomes (ALs) appear as mCherry-positive puncta. Autophagic activity is indicated by a shift to the red spectrum due to fluorescence quenching of the pH-sensitive-GFP by the acidic environment of the autolysosome. Scale bar, 50 μm. **p**, Quantification of ALs as depicted in **o**. Note that the statistics were performed together with additional groups as indicated in Extended Data Fig. 10c. *n* = 11 (CTL ad lib), 26 (CTL fasted), 8 (*Odc-1(RNAi)* ad lib), 30 (*Odc-1(RNAi)* fasted) worms. Statistics used were two-way ANOVA with Holm-Šídák’s multiple comparisons test (**b**,**d**,**f**,**g**,**h**,**j**,**n**), one-way ANOVA with Holm-Šídák’s multiple comparisons test (**l**) and Kruskal–Wallis test with FDR correction (two-stage step-up method by Benjamini, Krieger and Yekutieli, *Q* = 0.05) (**p**). Bar graphs show the mean ± s.e.m. Source numerical data and unprocessed blots are available in source data. NS, not significant. Source data

**Extended Data Fig. 3:**
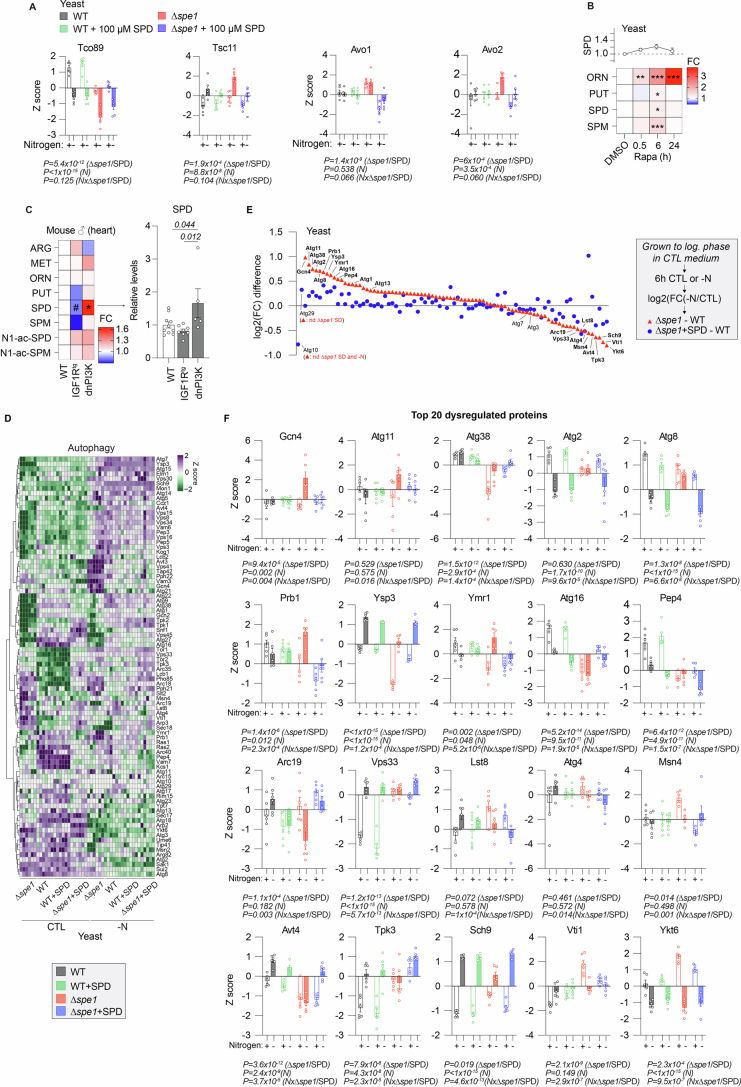
Spermidine is required for the correct shutdown of TORC1 and autophagy regulation in N-starving yeast. **(a)** Proteome change in *S. cerevisiae* WT BY4741 and Δ*spe*1 strains under specified condition treatments following a 6-hour culture in control or -N media with or without 100 µM SPD supplementation. Differential expression (Z-score) of proteins involved in the TORC complex, from the proteome analysis. N = 6 biologically independent samples (yeast cultures). **(b)** Polyamine levels of WT BY472 treated with rapamycin (40 nM) for the indicated times. Data normalized to the mean of the DMSO control group at every time point. N = 5 biologically independent samples (yeast cultures). **(c)** Polyamine and precursor levels in cardiac tissue of young, male control, transgenic IGF1^tg^ or dnPI3K mice. N = 5(WT), 8(IGF1R^tg^), 11(dnPI3K) mice. **(d)** Yeast WT and Δ*spe*1 strains under specified condition treatments following a 6-hour culture in control or -N media with or without 100 µM SPD. Differential expression (Z-Score) of proteins involved in autophagy, from the proteome analysis in Supplementary Fig. 1c. N = 6 biologically independent samples (yeast cultures). **(e)** Differential regulation of autophagy-relevant proteins in ∆*spe1* cells. The averaged log2-transformed fold change (FC) of individual proteins (-N compared to control medium) was calculated for all conditions. The graph depicts the differences of these log2(FC) between ∆*spe1* and WT cells (red triangles), as well as ∆*spe1* treated with 100 µM SPD and WT cells (blue dots). nd=not detected. N = 6 biologically independent samples (yeast cultures). **(f)** Z-scores of the top 20 dysregulated proteins, as identified in [E]. N = 6 biologically independent samples (yeast cultures). Statistics: [A,B,F] Two-way ANOVA with Holm-Šídák’s multiple comparisons test. [C] Two-way ANOVA with FDR correction (Two-stage step-up method by Benjamini, Krieger and Yekutieli, Q = 0.05). Heatmaps show means. Bar and line graphs show the mean ± s.e.m. * *P* < 0.05, ** *P* < 0.01, *** *P* < 0.001, # *P* < 0.2. Source numerical data are available in source data. Source data

Given the implication of TORC1 in the fasting response of ∆*spe1* yeast cells, we next asked whether ∆*spe1* cells would functionally alter the nitrogen deprivation-induced inhibition of TORC1. Sch9 (the yeast equivalent of mammalian p70^S6K^) dephosphorylation, was significantly delayed in *∆spe1* cells (Fig. 3a,b). Focusing on TORC-associated proteins in our proteome data, we found dysregulated TORC subunits, including Tco89, Avo1/2 and Tsc11, under both conditions in the ∆*spe1* strain (Extended Data Fig. 3a). Of note, low levels of polyamines (100 µM) completely reverted the delayed TORC1 inhibition in *∆spe1* cells (Fig. 3c,d). On the other hand, high levels of additional SPD (5 mM) did not affect TORC1 activity in the WT strain (Fig. 3e,f). Similar to −N, acute pharmacological inhibition of TORC1 with rapamycin led to a rapid increase of ORN (likely due to the known activation of arginase expression^37,38^) as well as elevated SPD and SPM levels (Extended Data Fig. 3b). Thus, the efficient shutdown of yeast TOR signalling upon −N, a key event for autophagy induction^39^, requires intact polyamine metabolism, whereas TOR inhibition promotes the anabolism of polyamines.

To translate these findings in vivo, we measured polyamines and precursors in hearts from young male mice overexpressing the human insulin-like growth factor 1 receptor (IGF1R^tg^)^40^ or carrying a dominant negative phosphoinositide 3-kinase mutant (dnPI3K)^41^ specifically in cardiomyocytes. IGF1R^tg^ causes increased IGF1R signalling, leading to elevated mTOR activity and autophagy inhibition, as well as age-associated heart failure, which can be overridden by SPD^42^. Conversely, the cardiomyocyte-specific dnPI3K mutation inhibits mTOR and enhances autophagic flux^42^. We observed a trend towards lower cardiac SPD levels in IGF1R^tg^ mice (*P* = *0.222*) and significantly elevated levels of SPD in dnPI3K mice (Extended Data Fig. 3c).

Collectively, blocking polyamine synthesis caused a defective cellular response to −N in yeast, thus compromising, inter alia, energy metabolism and amino acid homoeostasis, both of which interface with TOR signalling, an integral hub for sensing and relaying nutrient information to cellular responses and functional autophagy. SPD supplementation reversed the metabolic inflexibility of ∆s*pe1* cells.

### Spermidine is vital for autophagy induction during fasting

mTOR is a major repressor of autophagic flux and proteomics revealed a profound dysregulation of autophagy-relevant proteins upon *SPE1* loss (Extended Data Fig. 3d–f). SPD has been previously shown to induce *ATG7* expression in yeast^23^, and accordingly, *SPE1* knockout caused reduced Atg7 protein expression (Fig. 3g). Furthermore, several autophagy-relevant proteins were differently modulated in the ∆*spe1* strain upon −N (Extended Data Fig. 3d–f). This included the transcription factors Gcn4 (for amino acid biosynthesis), Msn4 (stress response), several autophagy-related proteins (Atg2/8/11/16/38), as well as vacuolar proteinases (Prb1, Ysp3 and Pep4) and proteins involved in intracellular vesicle trafficking (Vps33, Arc19, Vti1 and Ykt6), among others.

**Extended Data Fig. 4:**
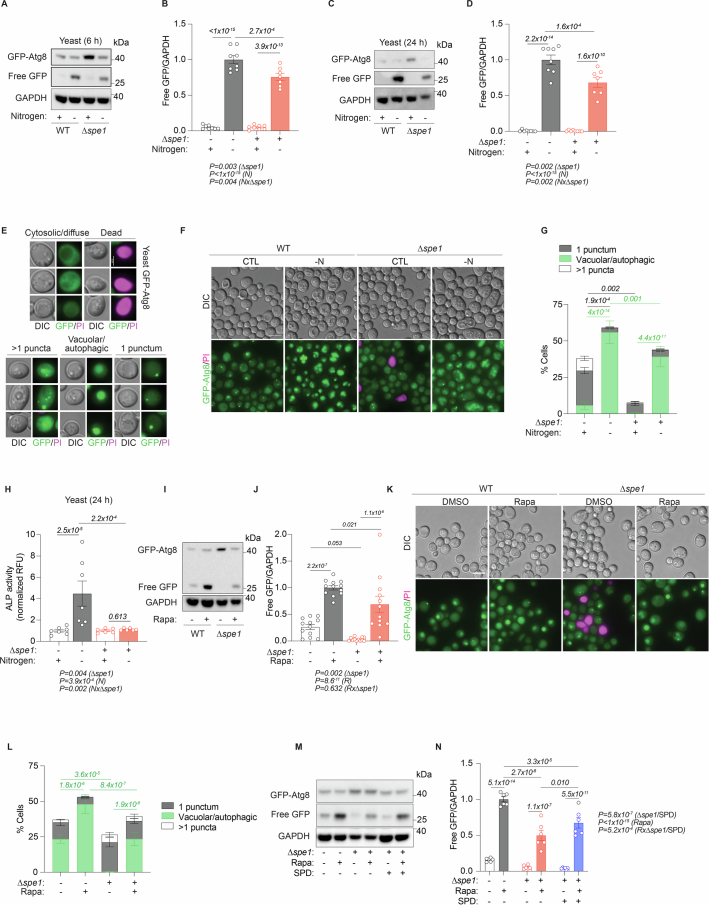
SPE1, the yeast ODC1 homologue, is required for starvation- and rapamycin-induced autophagy. **(a)** Representative immunoblots of yeast WT and ∆s*pe1* GFP-Atg8 after 6 hours -N, assessed for GFP and GAPDH. **(b)** Quantifications of [B]. N = 7(∆*spe1* -N), 8 (rest) biologically independent samples (yeast cultures). **(c)** Representative immunoblots of yeast WT and ∆s*pe1* GFP-Atg8 after 24 hours -N, assessed for GFP and GAPDH. **(d)** Quantifications of [C]. N = 7(∆*spe1* -N), 8(rest) biologically independent samples (yeast cultures). **(e)** Representative images of the categories used for categorization of GFP-Atg8 signals as shown and quantified in [F-G]. DIC = differential interference contrast, PI = propidium iodide staining for dead cells. **(f)** Representative images of yeast WT and ∆*spe1* GFP-Atg8 cells 6 hours after -N. Scale bar = 5 µm. **(g)** Blinded manual quantification of the autophagic status in microscopy images of WT and ∆*spe1* GFP-Atg8 cells 6 hours after -N. N = 6 biologically independent samples (yeast cultures). **(h)** ALP activity (RFU/µg) from Pho8∆N60 assay normalized to each CTL group after 24 hours -N. N = 7(WT), 6(∆*spe1*) biologically independent samples (yeast cultures). **(i)** Representative immunoblots of yeast WT and ∆s*pe1* GFP-Atg8 after 6 hours rapamycin treatment (40 nM), assessed for GFP and GAPDH. **(j)** Quantifications of [I]. N = 12 biologically independent samples (yeast cultures). **(k)** Representative images of yeast WT and ∆*spe1* GFP-Atg8 cells 6 hours after rapamycin (40 nM) treatment. Scale bar = 5 µm. **(l)** Blinded manual quantification of the autophagic status in microscopy images of WT and ∆*spe1* GFP-Atg8 cells 6 hours after rapamycin (40 nM) treatment. N = 6 biologically independent samples (yeast cultures). **(m)** Representative immunoblots of yeast WT BY4742 and ∆s*pe1* GFP-Atg8 after 6 hours rapamycin treatment (40 nM), with and without 100 µM SPD, assessed for GFP and GAPDH. **(n)** Quantifications of [M]. N = 6 biologically independent samples (yeast cultures). Statistics: Two-way ANOVA with Holm-Šídák’s multiple comparisons test. Bar graphs show the mean ± s.e.m. Source numerical data and unprocessed blots are available in source data. Source data

As a functional consequence, ∆*spe1* cells exhibited reduced autophagy induction in response to −N. We observed a diminished autophagy-dependent proteolytic liberation of green fluorescent protein (GFP) from GFP fused to autophagy-related protein 8 (GFP-Atg8, Atg8 being the yeast orthologue of the mammalian LC3 family) (Extended Data Fig. 4a–d), which was rescued by SPD supplementation (Fig. 3h,i). This was confirmed with additional autophagy assays, including the reduced redistribution of GFP-Atg8 towards autophagic vacuoles, compared with WT cells (Extended Data Fig. 4e–g) and the Pho8∆N60 assay^43^ (Fig. 3j and Extended Data Fig. 4h). However, supplementing SPD could not further elevate autophagic flux under −N in WT cells (Fig. 3k,l). Similarly, rapamycin-induced autophagy was significantly curtailed in ∆*spe1* cells (Extended Data Fig. 4i–l), which again could be partly rescued by SPD (Extended Data Fig. 4m,n).

**Extended Data Fig. 5:**
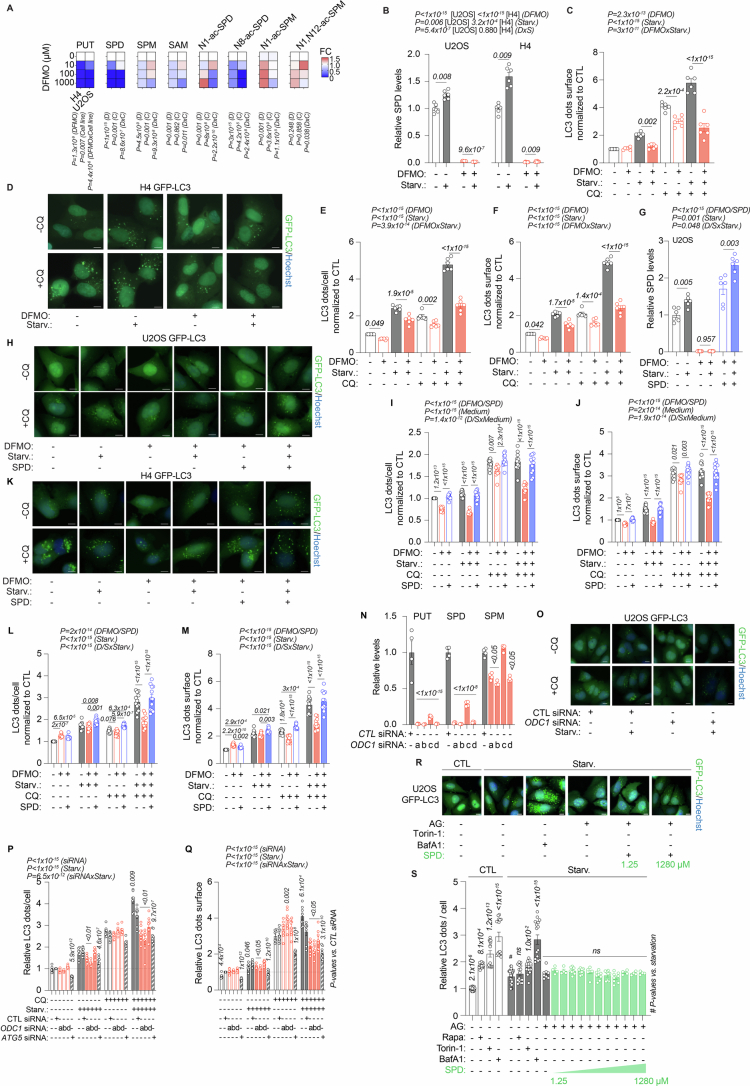
ODC1 is required for starvation-induced autophagy in H4 and U2OS cells. **(a)** DFMO treatment (48 hours, 100 µM) affects the polyamine profile of H4 and U2OS cells *in vitro*. Polyamine levels normalized to cell line-specific control (0 µM DFMO). N = 5(H4 0, 100 µM DFMO), 4(rest) biologically independent samples. **(b)** SPD levels in DFMO-treated (48 hours, 100 µM) U2OS and H4 cells after 6 hours starvation. N = 6 biologically independent samples. **(c)** Quantification of surface area covered by GFP-LC3 dots in 6 hours starved U2OS GFP-LC3 cells treated with or without 100 µM DFMO for 3 days, as depicted in Fig. 3m, normalized to the control condition. N = 6 biologically independent experiments. **(d)** Representative images of human H4 GFP-LC3 cells starved for 3 hours in HBSS (with or without chloroquine [CQ] for 1.5 hours before fixation) after three days of 100 µM DFMO treatment. Scale bar = 10 µm. **(e, f)** Quantification of cytosolic GFP-LC3 dots and surface area covered by GFP-LC3 dots from [D], normalized to the control condition. N = 6 biologically independent experiments. **(g)** SPD levels in U2OS cells treated with DFMO (100 µM) and SPD (10 µM) after 6 hours of starvation. Aminoguanidine (1 mM) was added to all conditions. N = 6 biologically independent samples. **(h-j)** Representative images and quantifications of human U2OS GFP-LC3 cells starved for 6 hours in HBSS (with or without chloroquine [CQ] for 3 hours before fixation) after three days of 100 µM DFMO treatment in combination with or without 10 µM SPD. Aminoguanidine (1 mM) was added to all conditions. Scale bar = 10 µm. N = 12 biologically independent experiments. **(k-m)** Representative images and quantifications of human H4 GFP-LC3 cells starved for 3 hours in HBSS (with or without CQ for 1.5 hours before fixation) after three days of 100 µM DFMO treatment in combination with or without 10 µM SPD. Aminoguanidine (1 mM) was added to all conditions. Bar = 10 µm. N = 12 biologically independent experiments. **(n)** Polyamine levels are depleted 48 hours after *ODC1* knockdown via siRNAs in U2OS cells. N = 4 biologically independent samples. **(o-q)** Representative images and quantifications of human U2OS GFP-LC3 cells starved for 6 hours in HBSS (with or without CQ for 3 hours before fixation) after three days of *ODC1* knockdown. Scale bar = 10 µm. N = 8 biologically independent experiments. **(r, s)** Representative images and quantifications of human U2OS GFP-LC3 cells starved for 6 hours in HBSS or treated with Rapamycin (10 µM) or Torin-1 (300 nM). SPD was added in ascending concentrations to test for synergistic effects with starvation. AG = aminoguanidine (1 mM). N = 4(Starv.+AG + 1.25, 2.5, 320, 640, 1280 µM SPD), 8(Starv.+AG; Starv+AG + 5-160 µM SPD), 16(Rapa, Torin-1, BafA1), 32(CTL) biologically independent samples. Statistics: [A-C,E-G,I-J,L-N,P-Q] Two-way ANOVA with Holm-Šídák’s multiple comparisons test. [S] One-way ANOVA with Holm-Šídák’s multiple comparisons test Heatmaps show means. Bar graphs show the mean ± s.e.m. Source numerical data are available in source data. Source data

The ODC1 inhibitor difluoromethylornithine (DFMO) depleted polyamines in human U2OS and H4 in nutrient-rich (Extended Data Fig. 5a) and starved (Extended Data Fig. 5b) conditions to <5% of control levels, hence excluding the possibility that *ODC1*-independent mechanisms (such as uptake of exogenous SPD) would account for the starvation-induced elevation of SPD. DFMO reduced the translocation of GFP–LC3 to autophagosomes and autolysosomes in response to starvation in the presence of the lysosomal inhibitor chloroquine (CQ), indicating that polyamine synthesis is required for autophagic flux (Fig. 3m,n and Extended Data Fig. 5c–f). These phenotypes could be rescued by co-treatment with 10 µM SPD (Extended Data Fig. 5g–m). Three siRNAs targeting *ODC1* phenocopied the effects of DFMO, hence depleting intracellular polyamines from U2OS cells (Extended Data Fig. 5n) and dampening starvation-induced autophagic flux similar to *ATG5* siRNA (Extended Data Fig. 5o–q). As found in yeast, SPD supplementation failed to further enhance autophagy in starved U2OS GFP–LC3 cells (Extended Data Fig. 5r,s). Moreover, mTORC1 inhibition by rapamycin or torin-1 induced DFMO-inhibitable autophagy in U2OS and H4 cells (Supplementary Fig. 3a–f), which was rescued by SPD (Supplementary Fig. 3g–l).

**Extended Data Fig. 6:**
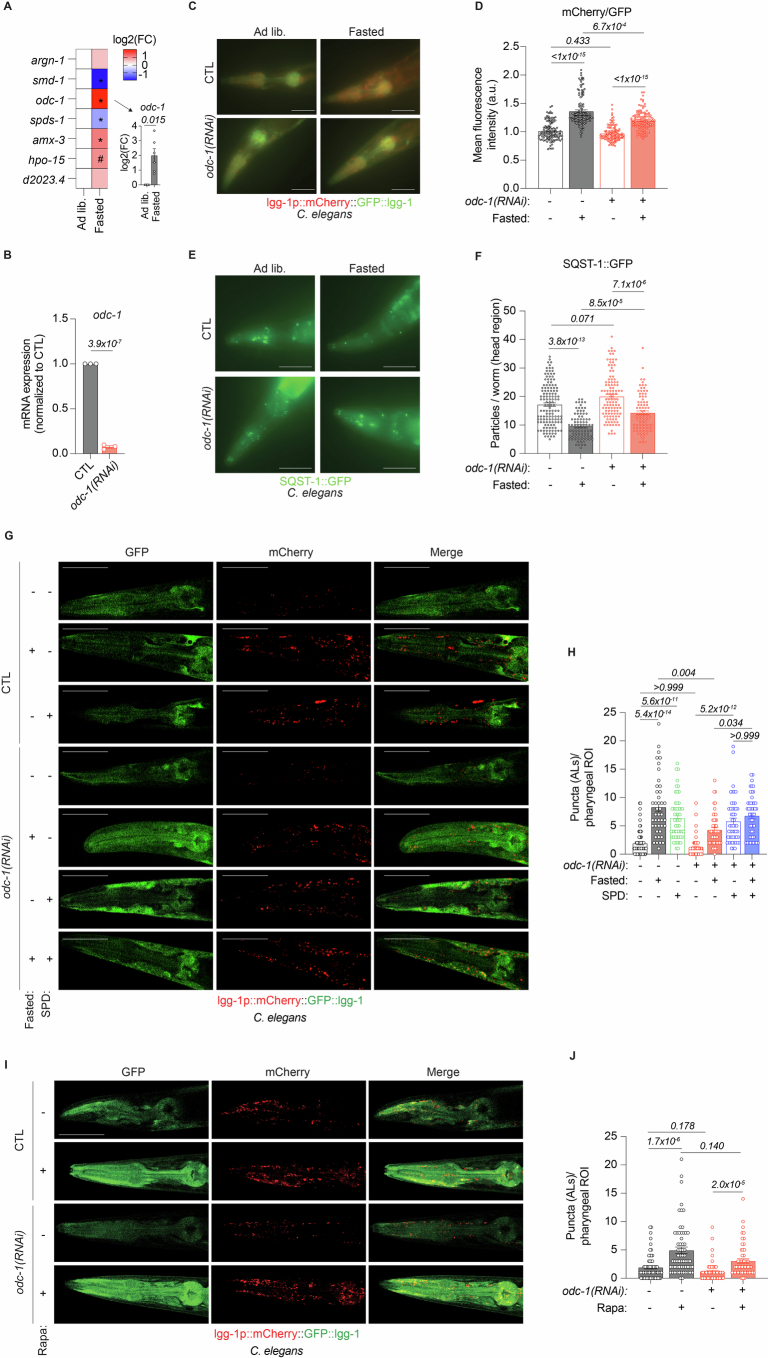
Fasting-induced autophagy in worms requires *odc-1.* **(a)** Relative mRNA expression levels of polyamine-relevant genes in 24 hours fasted *C. elegans*. N = 4(*hpo-15*), 5(*argn-1*, *smd-1*, *amx-3, d2023.4*), 6(*odc-1*, *spds-1*) biologically independent experiments. **(b)** Knockdown efficiency of *odc-1* mRNA by feeding bacteria expressing *odc-1(RNAi)* for 3 days in *C. elegans*. N = 3 biologically independent experiments. **(c)** Representative fluorescence images of the head region of young *C. elegans* MAH215 (sqIs11 [lgg-1p::mCherry::GFP::lgg-1 + rol-6]) fasted for two days and fed control or *odc-1(RNAi)* expressing bacteria. Autophagic activity is indicated by a shift to the red spectrum due to fluorescence quenching of the pH-sensitive-GFP by the acidic environment of the autolysosome. Scale bar = 50 μm. **(d)** Quantification of the ratio of the mean fluorescence intensity of mCherry/GFP signals, as depicted in [C]. Note that the experiment and statistics were performed together with the *dhps-1(RNAi)* groups in Extended Data Fig. 10e. N = 121(CTL ad lib), 127(CTL fasted), 113(*Odc-1(RNAi)* ad lib), 125(*Odc-1(RNAi)* fasted) worms. **(e)** Representative fluorescence images of the head region of young *C. elegans* SQST-1::GFP fasted for two days and fed control or *odc-1(RNAi)*. Autophagic activity is indicated by a decrease in the number of GFP-positive particles. Scale bar = 50 μm. **(f)** Quantification of the SQST-1::GFP particles in the head region, as depicted in [E]. Note that the experiment and statistics were performed together with the *dhps-1(RNAi)* groups in Extended Data Fig. 10g. N = 133(CTL ad lib), 86(CTL fasted), 109(*odc-1(RNAi)* ad lib), 86(*odc-1(RNAi)* fasted) worms. **(g)** Representative images of the head region of young *C. elegans* MAH215 (sqIs11 [lgg-1p::mCherry::GFP::lgg-1 + rol-6]) fasted for two days and fed control or *odc-1(RNAi)* with and without 0.2 mM SPD. Autolysosomes (ALs) appear as mCherry-positive puncta. Scale bar = 50 μm. **(h)** Quantification of ALs as depicted in [G]. N = 63(CTL), 42(CTL fasted), 52(CTL + SPD), 54(*odc-1*(RNAi)), 48(*odc-1*(RNAi) fasted), 50(*odc-1*(RNAi)+SPD, *odc-1*(RNAi)+SPD fasted) worms. **(i)** Representative images of the head region of young *C. elegans* MAH215 (sqIs11 [lgg-1p::mCherry::GFP::lgg-1 + rol-6]) with or without 50 µM rapamycin and fed control or *odc-1(RNAi)*. Autolysosomes (ALs) appear as mCherry-positive puncta. Scale bar = 50 μm. **(j)** Quantification of ALs as depicted in [I]. N = 63(CTL), 62(CTL+Rapa), 54(*odc-1*(RNAi)), 53(*odc-1*(RNAi)+Rapa) worms. Statistics: [A] Mann-Whitney with Holm-Šídák’s multiple comparisons test. [B] Two-tailed Student’s *t*-test. [D,F,H,J] Kruskal-Wallis test with Dunn’s multiple comparison test. Heatmaps show means. Bar graphs show the mean ± s.e.m. * *P* < 0.05, # *P* < 0.2. Source numerical data are available in source data. Source data

*C.* *elegans* subjected to acute fasting exhibited elevated messenger RNA levels of *odc-1*, *amx-3* and *hpo-15* (PAOX and SMOX orthologues), whereas spermidine synthase (*spds-1*) and the AMD1 orthologue *smd-1* decreased. Among the tested polyamine-relevant genes, *argn-1* (ARG1/2 orthologue) and *d2023.4* (SAT1/2) remained unaffected (Extended Data Fig. 6a). Knockdown of *odc-1* by RNA interference (Extended Data Fig. 6b) reduced fasting-induced autophagic flux assessed by a tandem-tagged (mCherry/GFP) LGG-1 fluorescent reporter, the worm equivalent of yeast Atg8 and human LC3 (Fig. 3o,p and Extended Data Fig. 6c,d). This was confirmed in a strain expressing an alternative autophagy-sensitive biosensor, GFP-fused SQST-1 (the worm orthologue of human sequestosome 1 (SQSTM1)/p62, an autophagy substrate), which was less degraded in fasted *odc-1* knockdown worms than in control nematodes (Extended Data Fig. 6e,f). Notably, SPD feeding reverted the autophagic deficit of *odc-1* worms (Extended Data Fig. 6g,h). Similar to yeast, *odc-1* knockdown also caused a trend (*P* = 0.140) towards reduced number of rapamycin-induced autolysosomes (Extended Data Fig. 6i,j).

Altogether, these findings indicate that ODC1 is required for optimal fasting-induced autophagy across species.

### Spermidine is required for fasting-mediated lifespan extension

**Fig. 4:**
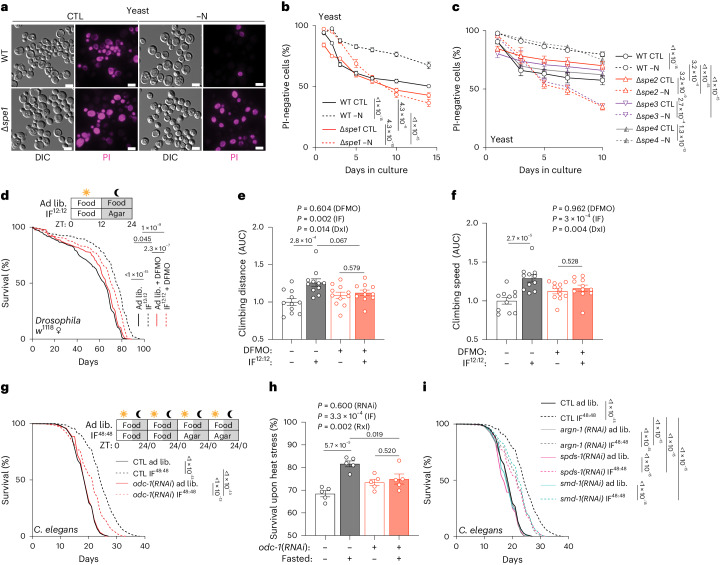
Intermittent fasting-mediated lifespan extension depends on SPD synthesis. **a**, Representative microscopy images on day 5 of chronological lifespan experiments of yeast WT and ∆*spe1* in control and −N medium, stained with propidium iodide (PI). Scale bar, 5 µm. **b**, PI-negative (live) cells during chronological aging of yeast WT and ∆*spe1* in control and −N medium. *n* = 36 (WT), 26 (∆*spe1* CTL), 24 (∆*spe1* −N) biologically independent samples (yeast cultures). **c**, PI-negative (live) cells during chronological aging yeast WT and ∆*spe2*, ∆*spe3* and ∆*spe4* cells grown to the log phase and shifted to CTL or −N. *n* = 8 biologically independent samples (yeast cultures). **d**, Lifespan of female *w*^*1118*^ flies fed standard food with or without 10 mM DFMO and subjected to IF^12:12^. *n* = 315 (ad lib), 313 (IF), 327 (ad lib + DFMO), 348 (IF + DFMO) flies. **e**, Flies from **d** were assessed for their climbing ability, measured as covered walking distance after a negative geotaxis stimulus, between days 53–60. *n* = 11 biologically independent samples. **f**, Flies from **d** were assessed for their climbing ability, measured as speed after a negative geotaxis stimulus, between days 53–60. *n* = 11 biologically independent samples. **g**, Lifespan of *C.* *elegans* N2 fed control (CTL) or *odc-1(RNAi)* expressing bacteria during IF^48:48^. The worms were transferred every other day. IF groups were transferred to agar plates without bacteria every second day. Note that the statistics and experiments were performed together with the groups depicted in **i** and Fig. 6p. *n* = 913 (CTL ad lib), 750 (CTL IF), 794 (*odc-1(RNAi)* ad lib), 779 (*odc-1(RNAi)* IF) worms. **h**, The susceptibility of worms to heat stress is reduced by IF^48:48^ in control, but not in worms fed *odc-1(RNAi)* expressing bacteria. Young worms (after the first round of fasting) were placed at 37 °C for 6 h. Survival was assessed after overnight recovery at 20 °C. *n* = 5 biologically independent experiments. **i**, Lifespan of *C.* *elegans* N2 fed bacteria expression control (CTL) or RNAi against *argn-1*, *spds-1* or *smd-1* during IF^48:48^. Note that the experiments and statistics were performed together with the groups depicted in **g** and Fig. 6p. *n* = 913 (CTL ad lib), 750 (CTL IF), 899 (*argn-1(RNAi)* ad lib), 797 (*argn-1(RNAi)* IF), 820 (*smd-1(RNAi)* ad lib), 803 (*smd-1(RNAi)* IF), 881 (*spds-1(RNAi)* ad lib), 746 (*spds-1(RNAi)* IF) worms. Statistics used were two-way ANOVA with Holm-Šídák’s multiple comparisons test (**b**,**c**,**e**,**f**,**h**) and log-rank test with Bonferroni correction (**d**,**g**,**i**). Bar graphs show the mean ± s.e.m. Source numerical data are available in source data. AUC, area under the curve. Source data

Nitrogen deprivation reduces the fraction of dead cells in chronological aging experiments performed on yeast, a model for post-mitotic aging^44^, in an autophagy-dependent manner^45^. This longevity-extending effect was abolished in ∆*spe1* cells, indicating that polyamine synthesis is required for longevity upon −N (Fig. 4a,b). The knockout of spermidine synthase (∆*spe3*) and that of SAM decarboxylase (∆*spe2*), which are both required for SPD generation, phenocopied ∆*spe1* with respect to the loss of the longevity during −N (Fig. 4c). In contrast, the knockout of spermine synthase (∆*spe4*) failed to affect survival under nitrogen-deprived conditions (Fig. 4c). Of note, survival deficits triggered by the loss of Spe1 could be fully rescued by the addition of PUT, SPD or SPM (*P* > 0.05 against each other under nitrogen starvation) (Supplementary Fig. 4b). However, increasing concentrations of SPD only improved the nitrogen starvation-prolonged chronological lifespan of WT cells on early time points (Supplementary Fig. 4c).

We next investigated the involvement of *SPE1* in other lifespan-increasing interventions in yeast. Inhibition of TOR with rapamycin extends yeast lifespan in an autophagy-dependent fashion^46,47^, and this effect was diminished in the ∆*spe1* strain (Supplementary Fig. 4d) but rescued by polyamine supplementation (Supplementary Fig. 4e). The extension of chronological lifespan by glucose restriction was partially compromised by the *spe1* knockout (Supplementary Fig. 4f). Replicative aging, which reflects the diminished replicative capacity of aging mother cells^44^, is especially responsive to glucose restriction. Knockout of *SPE1* diminished the survival, median (WT 23 versus ∆s*pe1* 19 days) and maximal replicative lifespan (68 versus 44 days) in low-glucose (0.05%) cultures but did not affect replicative lifespan when glucose concentrations were kept at standard levels (2%) (Supplementary Fig. 4g). In summary, *SPE1* and SPD are essential for longevity induction by nitrogen starvation, rapamycin and glucose depletion.

**Extended Data Fig. 7:**
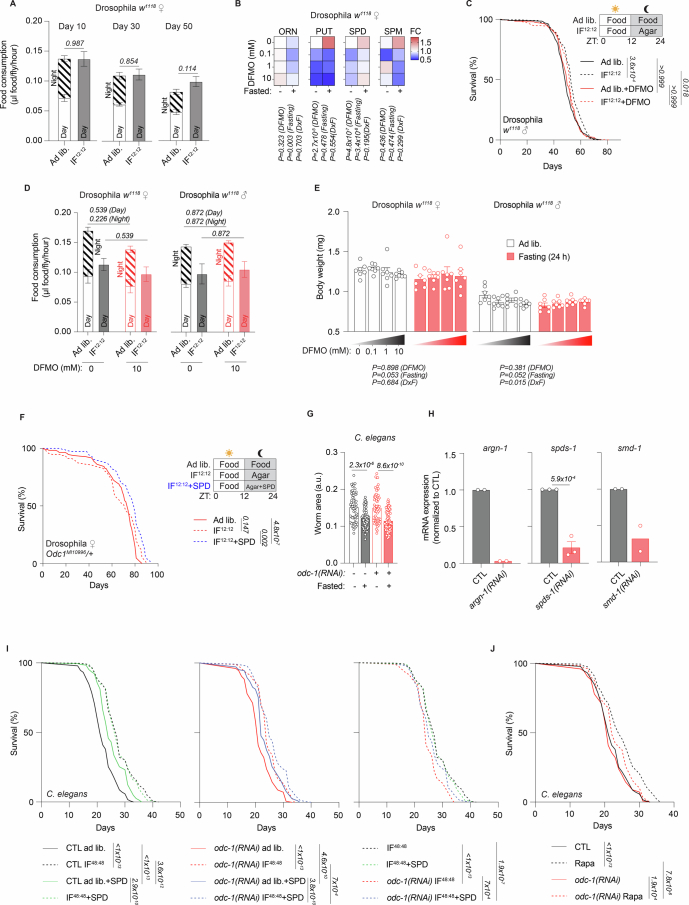
Polyamine synthesis is important for IF-induced benefits in flies and worms. **(a)** Daily and nightly food consumption of *D. melanogaster w*^*1118*^ after 10, 30 and 50 days of IF^12:12^. N = 11(Day 30 IF Day), 12(Day 30 Ad lib), 16(Day 50 Ad lib Night), 18(Day 10), 20(Day 50 IF Day), 22(Day 50 Ad lib Day) biologically independent samples (groups of flies). **(b)** DFMO feeding affects the polyamine profile of female *w*^*1118*^ flies during ad lib and 24 hours fasting. N = 6(Ad lib 10 µM DFMO; Fasted 0, 10 µM DFMO), 7(Ad lib 0, 0.1, 1 µM DFMO; Fasted 0.1, 1 µM DFMO) biologically independent samples (groups of flies). **(c)** Lifespan of male *w*^*1118*^ flies fed standard food with or without 10 mM DFMO and subjected to IF. N = 210(Ad lib), 206(IF), 199(Ad lib+DFMO), 212(IF + DFMO) flies. **(d)** Food consumption of 10-day old female and male *w*^*1118*^ flies, fed control or food containing 10 mM DFMO, during the first 7 cycles of IF. N = 8(0 mM Day IF), 9(rest) biologically independent samples (groups of 5 flies per N). **(e)** DFMO feeding does not influence body weight changes after 24 hours fasting. N = 7 biologically independent samples (groups of flies). **(f)** Lifespan of heterozygous *Odc1*^*MI10996*^*/+* flies subjected to IF. The IF + SPD group received 5 mM SPD via agar during the night. N = 119(*Odc1*^*MI10996*^*/+* ad lib), 117(*Odc1*^*MI10996*^*/+* IF), 113(*Odc1*^*MI10996*^*/+* IF + SPD) worms. **(g)** Knockdown of *odc-1* does not affect worm size under ad lib or IF^48:48^ conditions, compared to CTL RNAi. Note that the experiment and statistics were performed together with the *dhps-1(RNAi)* groups in Extended Data Fig. 10h. N = 56(CTL ad lib), 66(CTL IF), 63(*Odc-1(RNAi)* ad lib), 78(*Odc-1(RNAi)* IF) worms. **(h)** Knockdown efficiency of respective mRNAs by feeding bacteria expressing *argn-1*, *spds-1*, or *smd-1* RNAi in *C. elegans*. N = 2(*argn-1*, *smd-1*), 3(*spds-1*) biologically independent experiments. **(i)** Lifespan of *C. elegans* N2 fed control (CTL) or RNAi against *odc-1*, with or without continuous 0.2 mM SPD feeding during IF. Note that the experiments and statistics were performed together with the groups depicted in [J]. N = 429(CTL ad lib), 523(CTL IF), 437(CTL ad lib+SPD),466(IF + SPD), 330(*odc-1(RNAi)* ad lib), 575(*odc-1(RNAi)* IF), 497(*odc-1(RNAi)* ad lib+SPD), 605(*odc-1(RNAi*) IF + SPD) worms. **(j)** Lifespan under 50 µM rapamycin treatment of *C. elegans* N2 fed control (CTL) or RNAi against *odc-1*. Note that the experiments and statistics were performed together with the groups depicted in [I]. N = 429(CTL), 423(Rapa), 330(*odc-1(RNAi)*, 330(*odc-1(RNAi)* Rapa) worms. Statistics: [A,D] Two-tailed Student’s *t*-test (per time point). [B,E,G] Two-way ANOVA with Holm-Šídák’s multiple comparisons test. [F,I,J] Log-rank test with Bonferroni correction. [H] Two-tailed Student’s *t*-test. Heatmaps show means. Bar graphs show the mean ± s.e.m. Source numerical data are available in source data. Source data

Testing these findings’ relevance, we subjected fruit flies to an IF regime that improves healthspan and lifespan. We followed the survival of female and male *w*^*1118*^ flies under IF^12:12^, during which increased daytime food intake compensated for the nightly calorie loss after 10 cycles and at later time points (Extended Data Fig. 7a). DFMO lowered whole-body SPD levels (Extended Data Fig. 7b) and reduced the effects of IF^12:12^ on improved survival in both sexes (Fig. 4d, Extended Data Fig. 7c, Supplementary Table 2). Generally, IF^12:12^ seemed more effective in female flies, which we further tested for age-sensitive locomotor capacity with a modified negative geotaxis assay. DFMO prevented locomotion improvement by IF^12:12^ (Fig. 4e,f). Of note, DFMO did not affect food consumption during the first cycles of IF^12:12^ (Extended Data Fig. 7d) or body weight during acute fasting (Extended Data Fig. 7e) and generally seemed to be non-toxic for flies. Female flies lacking one functional copy of Odc1 (*Odc1*^*MI10996*^ mutant) were also unresponsive to IF^12:12^, and nightly SPD feeding could re-instate IF^12:12^-mediated longevity in such flies (Extended Data Fig. 7f and Supplementary Table 2).

In *C.* *elegans*, genetic inhibition of *odc-1* reduced the lifespan extension (Fig. 4g and Supplementary Table 2) and heat stress resistance (Fig. 4h) conferred by IF^48:48^, but did not affect the body size of fasted worms (Extended Data Fig. 7g). Knockdown of *spds-1* (spermidine synthase) *or smd-1* (adenosylmethionine decarboxylase 1), which are critical for SPD synthesis, as well as *argn-1* (Extended Data Fig. 7h) reduced the lifespan extension elicited by IF^48:48^ in worms (Fig. 4i and Supplementary Table 2). Notably, SPD significantly extended the lifespan of intermittently fasted *odc-1* knockdown worms towards that of WT controls (Extended Data Fig. 7i and Supplementary Table 2). However, SPD did not modulate the IF^48:48^ effect on the lifespan of WT worms and was less potent than IF alone when fed ad libitum (Extended Data Fig. 7i and Supplementary Table 2). Similarly, rapamycin-triggered longevity was blunted upon *odc-1* knockdown in worms (Extended Data Fig. 7j and Supplementary Table 2).

Altogether, these findings unravel a pathway in which endogenous polyamine biosynthesis mediates the lifespan-extending effects of nitrogen starvation in yeast and IF in flies and worms.

### Cardioprotective and antiarthritic effects of intermittent fasting are blunted by DFMO feeding in mice

We next determined whether endogenous SPD biosynthesis in mice was essential for IF-induced healthspan improvements, focusing on cardioprotection and suppression of inflammation, knowing that both are elicited by SPD supplementation^24,48^.

**Fig. 5:**
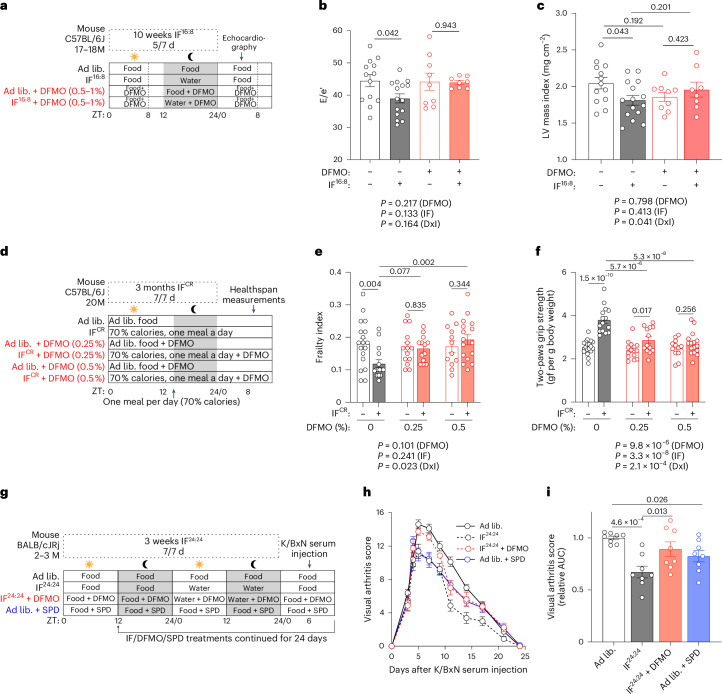
DFMO blunts the healthspan-promoting effects of IF in mice. **a**, Experimental layout to study the effects of DFMO feeding on IF^16:8^-mediated cardioprotection in 17–18-month-old male C57BL/6J mice. Mice were fasted from 15:00 to 7:00 (16 h daily, excluding weekends). A subset of aged mice received DFMO in the drinking water. After 10 weeks, cardiac function and structure were assessed by echocardiography. Data are shown in **b**,**c**, Extended Data Fig. 8a–h and Supplementary Table 3. **b**, Ratio of peak early Doppler transmitral flow velocity (E) to myocardial tissue Doppler velocity (e′), a measure of diastolic dysfunction, in aged male mice treated with IF^16:8^, with or without DFMO (*n* = 13, 15, 9 and 8 mice per group, respectively). **c**, LV mass normalized to body surface area (LV mass_i_). *n* = 13 (ad lib), 15 (IF), 9 (ad lib + DFMO), 8 (IF + DFMO) mice. **d**, Experimental layout to study the effects of DFMO feeding on IF^CR^-mediated healthspan improvements in 20-month-old male C57BL/6J mice. Mice were given a single meal per day (30% CR) shortly before the dark phase. A subset of mice received 0.25% or 0.5% DFMO in the drinking water. After 3 months, healthspan was assessed. Data are shown in **e**,**f** and Extended Data Fig. 8i–m. **e**, Visual frailty index of aged male mice treated with IF^CR^, with or without DFMO. *n* = 12 (ad lib 0.5%), 13 (IF 0.25%), 14 (ad lib 0.25%), 15 (IF 0%), 16 (IF 0.5%), 18 (ad lib 0%) mice. **f**, Grip strength of fore limbs in gram-force (gf) normalized to body weight of aged male mice treated with IF^CR^, with or without DFMO. *n* = 12 (ad lib 0.5%), 13 (IF 0.25%), 14 (ad lib 0.25%), 15 (IF 0%), 16 (IF 0.5%), 18 (ad lib 0%) mice. **g**, Experimental layout to study the effects of DFMO feeding on IF-mediated anti-inflammatory effects in young BALB/cJRj mice of both sexes. DFMO and 3 mM SPD were supplemented via drinking water. Mice were fasted every other day. IF^24:24^ and SPD treatments were started 3 weeks before serum transfer and continued until the end of the experiment. Data are shown in **h**,**i** and Extended Data Fig. 8n. **h**, Development of arthritis upon injection of serum from K/BxN mice in young male and female BALB/cJRj mice treated as outlined in **g**. *n* = 8 mice. **i**, AUC analysis of **h**. *n* = 8 mice. Statistics used were two-way ANOVA with Holm-Šídák’s multiple comparisons test (**b**,**c**,**e**,**f**), log-rank test with Bonferroni correction (**c**,**e**) and one-way ANOVA with Holm-Šídák’s multiple comparisons test (**i**). Bar graphs show the mean ± s.e.m. M, months of age. Source numerical data are available in source data. Source data

**Extended Data Fig. 8:**
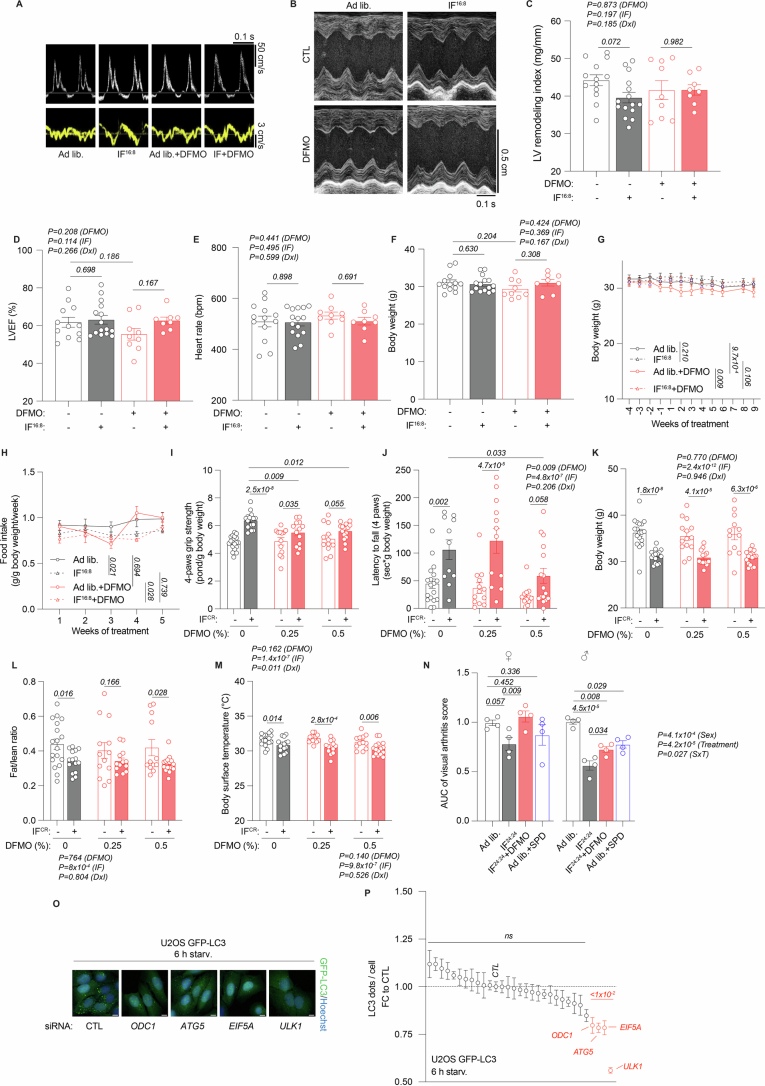
Cardiac profiling by echocardiography of aging mice during IF, with and without DFMO. **(a)** Representative echocardiography-derived mitral pulsed wave and tissue Doppler tracings in aged mice treated as outlined in Fig. 5a. **(b)** Representative echocardiography derived left ventricular (LV) M-mode tracings. **(c)** LV remodelling index. N = 13(Ad lib), 15(IF), 9(Ad lib + DFMO), 8(IF + DFMO) mice. **(d)** LV ejection fraction (LVEF). N = 12(Ad lib), 15(IF), 9(Ad lib + DFMO), 8(IF + DFMO) mice. **(e)** Heart rate. N = 13(Ad lib), 15(IF), 9(Ad lib + DFMO), 8(IF + DFMO) mice. **(f)** Body weight at the time of cardiac profiling. N = 13(Ad lib), 15(IF), 9(Ad lib + DFMO), 8(IF + DFMO) mice. **(g)** Body weight throughout the intervention. N = 15(Ad lib; Ad lib + DFMO),16(IF; IF + DFMO IF) mice (at week 0). **(h)** Food intake per mouse throughout the intervention. N = 3(Week 4 IF + DFMO), 4(rest) cages. **(i)** Grip strength (all limbs) normalized to body weight. N = 18(CTL ad lib), 15(CTL IF), 13(0.25% DFMO), 12(0.5% DFMO ad lib), 16(0.5% DFMO IF) mice. **(j)** Latency to fall in a 4-limb grid hanging test. N = 18(CTL ad lib), 11(CTL IF), 13(0.25% DFMO ad lib), 12(0.25% DFMO IF), 12(0.5% DFMO ad lib), 16(0.5% DFMO IF) mice. **(k)** Body weight. N = 18(CTL ad lib), 15(CTL IF), 14(0.25% DFMO ad lib), 13(0.25% DFMO IF), 12(0.5% DFMO ad lib), 16(0.5% DFMO IF) mice. **(l)** Fat-to-lean mass ratio. N = 18(CTL ad lib), 15(CTL IF), 13(0.25% DFMO), 12(0.5% DFMO ad lib), 16(0.5% DFMO IF) mice. **(m)** Abdominal surface temperature. N = 18(CTL ad lib), 15(CTL IF), 14(0.25% DFMO ad lib), 13(0.25% DFMO IF), 12(0.5% DFMO ad lib), 16(0.5% DFMO IF) mice. **(n)** Sex-stratified arthritis scoring of data presented in Fig. 5h-i upon injection of serum from K/BxN mice in young male and female BALB/cJRj mice treated as outlined in Fig. 5g. N = 4 mice. **(o)** Genes previously connected to the cellular effects of SPD were knocked out via siRNAs in U2OS GFP-LC3 cells and tested for starvation-induced autophagy. Selected representative images after 6 hours of starvation. **(p)** 48 hours after siRNA knockdown, U2OS GFP-LC3 cells were starved for 6 hours. The graph depicts the normalized individual FC to control conditions of GFP-LC3 dots for every gene knockdown or control condition (means ± S.E.M.). FC 1 represents the starvation-induced increase in GFP-LC3 dots in the control condition. Highlighted genes were significantly different from the control. N = 17(CTL), (Dharmafect control), 9(rest) biologically independent samples. Statistics: [C-N] Two-way ANOVA with Holm-Šídák’s multiple comparisons test. [P] One-way ANOVA with Holm-Šídák’s multiple comparisons test. Line graph shows the mean ± s.e.m. Bar and line graphs show the mean ± S.E.M. Source numerical data are available in source data. Source data

In aged male mice, DFMO abolished favourable cardiac effects of an IF^16:8^ protocol (daily 16 h fasting and 8 h ad libitum food access during the light phase) (Fig. 5a and Extended Data Fig. 8a,b). This concerned improvements in cardinal signs of cardiac aging^49^, including left ventricular (LV) diastolic dysfunction (E/e′) (Fig. 5b and Supplementary Table 3; *P* = 0.042, ad libitum versus IF), LV hypertrophy (Fig. 5c; *P* = 0.043) and LV remodelling index (*P* = 0.072) (Extended Data Fig. 8c and Supplementary Table 3), whereas other cardiac parameters remained unaffected by DFMO and IF^16:8^ (Extended Data Fig. 8d,e and Supplementary Table 3). DFMO did not alter the IF^16:8^-induced reduction of body weight and food intake (Extended Data Fig. 8f–h). In a second cohort of aged male mice, we tested the effects of DFMO on general and muscular healthspan improvements elicited by an IF + CR (30%) combination (IF^CR^; one meal a day, provided shortly before the dark phase) (Fig. 5d)^50^. DFMO prevented IF^CR^-mediated improvements in a visual frailty index^51^ (Fig. 5e), grip strength (Fig. 5f and Extended Data Fig. 8i) and wire hanging ability (Extended Data Fig. 8j), whereas it did not affect body weight (Extended Data Fig. 8k), body composition (Extended Data Fig. 8l) or body surface temperature (Extended Data Fig. 8m). Additionally, in young mice, both IF^24:24^ and oral SPD supplementation (Fig. 5g) ameliorated the progression of autoantibody-induced arthritis, and the antiarthritic effects of IF^24:24^ were blunted by parallel DFMO administration (Fig. 5h,i), with comparable results in female and male mice (Extended Data Fig. 8n).

Collectively, genetic or pharmacologic ODC1 inhibition attenuated or abolished the healthspan-promoting, cardioprotective and inflammation-regulating effects of IF regimens in multiple distantly related species. Therefore, SPD is probably a dominant pro-autophagic and anti-aging effector metabolite accounting for the beneficial effects of various forms of IF.

### Increased eIF5A hypusination is required for fasting-induced longevity

**Fig. 6:**
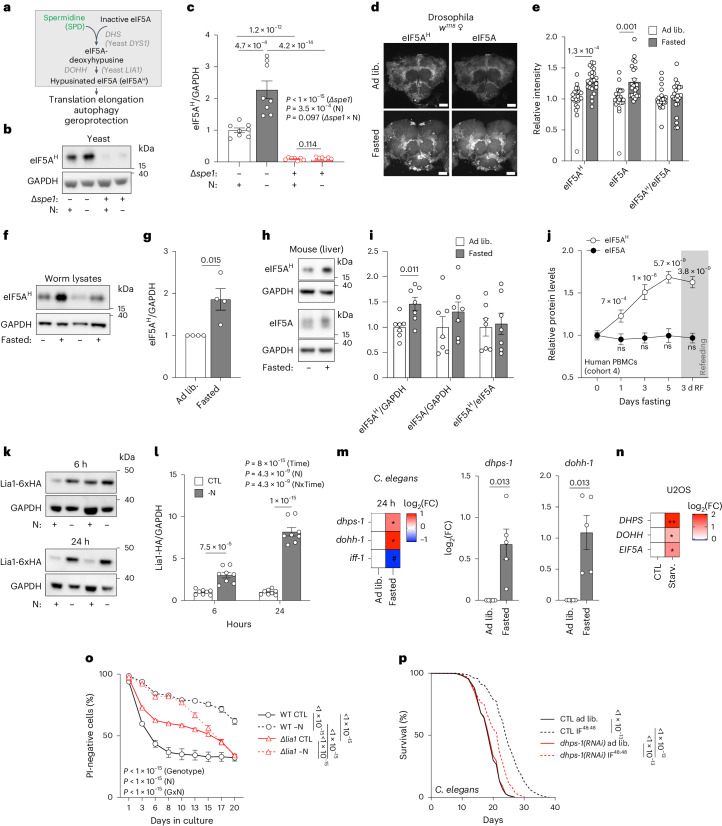
Hypusination of eIF5A acts downstream of spermidine to mediate IF-associated longevity. **a**, Scheme of the hypusination pathway. DHS, deoxyhypusine synthase; DOHH, deoxyhypusine hydroxylase; eIF5A, eukaryotic translation initiation factor 5A-1. **b**, Representative immunoblot of hypusinated eIF5A (eIF5A^H^) levels in yeast GFP-Atg8 WT and ∆*spe1* after 6 h of −N. **c**, Quantification of **b**. *n* = 8(WT), 7(∆*spe1*) biologically independent samples (yeast cultures). **d**, Representative maximum projection images of confocal microscopy images of female *w*^*1118*^ fly central brain regions probed for eIF5A and hypusine by immunofluorescence. Before dissection, the flies were fasted for 0 h (ad lib) or 12 h, starting at 20:00. Scale bar, 50 µm. **e**, Quantification of signal intensities in **d**. *n* = 22 (ad lib), 23 (fasted) fly brains. **f**, Immunoblots of 48 h fasted *C.* *elegans* assessed for hypusine and GAPDH. **g**, Quantification of **f**. *n* = 4 biologically independent samples (worm lysates). **h**, Representative immunoblot of eIF5A^H^, eIF5A and GAPDH signals of liver samples from ad lib and fasted (14–16 h) young, male C57BL/6 mice. **i**, Quantification of **h**. *n* = 7 mice. **j**, eIF5A^H^ and total eIF5A levels in human PBMCs after increasing fasting times, measured by capillary immunoblotting. RF, 3 days after re-introduction of food. *n* = 17 (days 0, 1 and 3), 15 (day 5), 16 (day 7 and RF) volunteers. **k**, Representative immunoblot of yeast Lia1-6xHA, assessed for HA-tags and GAPDH after 6 and 24 h −N. **l**, Quantification of Lia1-6xHA levels as depicted in **k**. *n* = 8 biologically independent samples (yeast cultures). **m**, Quantification of relative mRNA expression of *dhps-1*, *dohh-1* and *iff-1* in 24 h fasted *C.* *elegans*. *n* = 3 (*iff-1*), 5 (*dhps-1* and *dohh-1*) biologically independent experiments. **n**, Quantification of relative mRNA expression of *DHPS*, *DOHH* and *EIF5A* in 6 h starved U2OS cells. *n* = 3 (*DOHH)*, 4 (*DHPS* and *EIF5A*) biologically independent experiments. **o**, PI-negative (live) yeast cells during chronological lifespan analysis of WT and ∆*lia1* yeast in control and −N medium. *n* = 12 biologically independent samples (yeast cultures). **p**, Lifespan of *C.* *elegans* N2 fed control (CTL) or *dhps-1(RNAi)* (homologue of DHS) expressing bacteria during IF^48:48^. Note that the experiments and statistics were performed together with the groups depicted in Fig. 4g,i. *n* = 913 (CTL ad lib), 750 (CTL IF), 862 (*dhps-1(RNAi)* ad lib), 776 (*dhps-1(RNAi)* IF) worms. Statistics used were two-way ANOVA with Holm-Šídák’s multiple comparisons test (**c**,**j**,**l**,**o**), two-tailed Student’s *t*-tests with Holm-Šídák’s multiple comparisons test (**e**,**i**,**m**,**n**), two-tailed Student’s *t*-test (**h**) and log-rank test with Bonferroni correction (**p**). Bar and line graphs show the mean ± s.e.m. Source numerical data and unprocessed blots are available in source data. Source data

U2OS GFP–LC3 cells were screened for the effects of small interfering RNAs (siRNAs) targeting genes previously linked to SPD effects that hence might modulate starvation-induced autophagy. Besides the knockdown of *ODC1* and the autophagy-essential genes *ATG5* and *ULK1*, we found that depletion of *EIF5A* (eukaryotic translation initiation factor 5A) significantly reduced the number of GFP–LC3 dots after starvation (Extended Data Fig. 8o,p). SPD is required to hypusinate eIF5A via a conserved reaction involving deoxyhypusine synthase (DHS) and deoxyhypusine hydroxylase (DOHH)^52^. The resulting covalently modified and active hypusinated eIF5A (eIF5A^H^) is involved in the pro-autophagic and antiaging effects of SPD administration^53,54^ (Fig. 6a). We thus investigated whether the polyamine–eIF5A^H^ axis played a role in autophagy and lifespan regulation during −N and IF.

**Extended Data Fig. 9:**
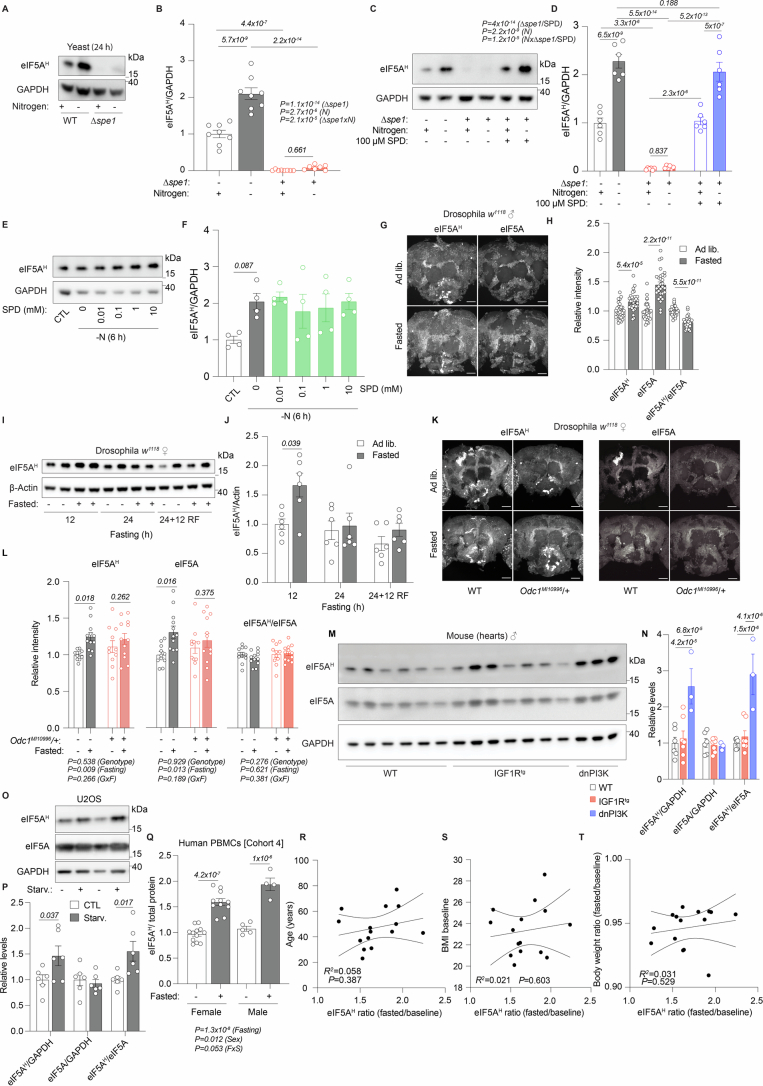
Fasting increases hypusination of eIF5A. **(a)** Hypusine levels are increased after 24 hours -N in yeast BY4741 GFP-Atg8 cells, but not in ∆*spe1*. Representative immunoblot. **(b)** Quantification of [A]. N = 7(∆*spe1* -N), 8(rest) biologically independent samples (yeast cultures). **(c)** Representative immunoblot of WT and ∆*spe1*, with and without 100 µM SPD after 6 hours -N, assessed for hypusine and GAPDH. **(d)** Quantification of [C]. N = 6 biologically independent samples (yeast cultures). **(e)** SPD supplementation does not further increase hypusination in BY4741 GFP-Atg8 yeast. Representative immunoblot. **(f)** Quantification of [E]. N = 4 biologically independent samples (yeast cultures). **(g)** Representative maximum projection images of confocal microscopy images of male *w*^*1118*^ fly central brain regions probed for eIF5A and hypusine by immunofluorescence. Prior to dissection, the flies were fasted for 0 (ad lib) or 12 hours, starting at 8 pm. Scale bar = 50 µm. **(h)** Quantification of signal intensities as shown in [G]. N = 30 fly brains. **(i)** Representative immunoblot of female Drosophila *w*^*1118*^ head extracts, assessed for hypusine and actin after 12 and 24 hours overnight fasting. RF = 12 hours refeeding. **(j)** Quantification of [I]. N = 6 biologically independent samples (fly head lysates). **(k)** Representative immunostaining microscopy images against hypusine after 12 hours fasting of female WT *w*^*1118*^ and *Odc1*^*MI10996*^/+ fly brains. Scale bar = 50 µm. **(l)** Quantification of [K]. N = 11(ad lib), 12(fasted) fly brains. **(m)** Immunoblots of cardiac tissue from young male transgenic IGF1R^tg^ or dnPI3K mice and their age-matched WT controls. Heart lysates were assessed for hypusine, total eIF5A and GAPDH. **(n)** Quantification of [M]. N = 6(WT), 7(IGF1R^tg^), 3(dnPI3K) mice. **(o)** Representative immunoblot of human U2OS cells starved for 6 hours and assessed for hypusine, total eIF5A and GAPDH. **(p)** Quantification of [O]. N = 6 biologically independent samples. **(q)** Hypusine levels after five days of fasting in isolated human PBMCs of cohort 4, stratified by sex. N = 12(female baseline), 11(female fasted), 5 (male baseline), 4(male fasted) volunteers. **(r-t)** eIF5A^H^ levels after five days of fasting in isolated human PBMCs of cohort 4 do not correlate with age, pre-fasting baseline BMI or body weight loss (body weight ratio). N = 15 volunteers. Statistics: [B,D,L,NP,Q] Two-way ANOVA with Holm-Šídák’s multiple comparisons test. [F] One-way ANOVA with Holm-Šídák’s multiple comparisons test. [H,J] Two-tailed Student’s *t*-test with Holm-Šídák’s multiple comparisons test. [R-T] Simple linear regression. Bar graphs show the mean ± s.e.m. Source numerical data and unprocessed blots are available in source data. Source data

Nitrogen deprivation of yeast enhanced eIF5A^H^ (Hyp2 in yeast) in a Spe1-dependent fashion (Fig. 6b,c and Extended Data Fig. 9a,b). The hypusination defect observed in Spe1-deficient cells was reversed by SPD (Extended Data Fig. 9c,d), which had no additional impact on hypusine levels in starved WT cells (Extended Data Fig. 9e,f).

Similarly, immunostaining of brains revealed increased eIF5A^H^ and total eIF5A in female and male flies after 12 h fasting (Fig. 6d,e and Extended Data Fig. 9g,h), as confirmed by immunoblotting of whole-head lysates from female flies (Extended Data Fig. 9i,j). Notably, female heterozygous *Odc1*^*MI10996*^ mutants, which failed to show lifespan extension upon IF^12:12^, also lost their eIF5A^H^ fasting response (Extended Data Fig. 9k,l).

We also found elevated eIF5A^H^ in lysates from fasted worms (Fig. 6f,g) and livers from fasted male mice (Fig. 6h,i). Of note, dnPI3K hearts, which had increased SPD levels, showed highly upregulated eIF5A^H^ levels, while the IGF1R^tg^ mutation caused no alterations in eIF5A^H^ or SPD levels (Extended Data Fig. 9m,n).

Increased eIF5A^H^, but not total eIF5A, levels were similarly detected in starved human U2OS cells (Extended Data Fig. 9o,p), as well as in PBMCs from healthy human volunteers over several days of fasting, which persisted after food re-introduction (Fig. 6j). Fasting enhanced eIF5A^H^ indistinguishably in male and female volunteers, and this effect was not affected by age, BMI or weight loss (Extended Data Fig. 9q–t).

We next explored the mechanisms of increased eIF5A^H^ levels. In yeast, protein levels of Dys1 (DHS) tendentially, but not significantly (*P* = 0.077), increased after 6 h and decreased after 24 h nitrogen deprivation, whereas Lia1 (yeast DOHH) was elevated significantly at both time points (Fig. 6k,l and Supplementary Fig. 5a,b), suggesting a superior role for DOHH in driving or maintaining the observed effects on eIF5A^H^. Compared with WT cells, ∆*spe1* cells exhibited increased Lia1 protein levels in control but not −N conditions, and this phenotype could be corrected by SPD (Supplementary Fig. 5c,d). In double knockout ∆*spe1*∆*lia1* cells, SPD failed to enhance eIF5A^H^ levels in control and −N medium (Supplementary Fig. 5e,f), indicating that Lia1 is indeed responsible for the hypusination response.

Similarly, increased mRNA transcript levels of *dohh-1* (*C.* *elegans* DOHH) and *dhps-1* (DHS), but not *iff-1* (*EIF5A*) were found in worms after 24 h fasting (Fig. 6m). Only DOHH levels stayed elevated after prolonged 48 h fasting (Supplementary Fig. 5g). In U2OS cells, 6 h starvation increased the abundance of mRNAs transcribed from all three genes (Fig. 6n).

Next, we compared the proteomic landscape during nitrogen deprivation in yeast cells treated with GC7, a specific pharmacological inhibitor of DHS/DYS1, and/or lacking *SPE1*. As previously observed, proteins involved in autophagy, TOR signalling, translation and amino acid metabolism were dysregulated when polyamine synthesis or hypusination were suppressed (Supplementary Fig. 5h–k), implying a multipronged effect of intracellular polyamine metabolism on these processes. Targeting hypusination directly with GC7 phenocopied many of the proteomic perturbations observed in the ∆*spe1* strain, supporting a role of eIF5A^H^ as a major downstream mediator of polyamine effects on energy and amino acid metabolism, as well as on TOR signalling and autophagy (Supplementary Fig. 5h–k). Additionally, ribosomal processes, which are known to require polyamines^55,56^, were strongly decreased when *SPE1* was lacking and/or eIF5A^H^ was inhibited by GC7 (Supplementary Fig. 5h,j). Accordingly, deleting the starvation-responsive *LIA1* gene significantly decreased the long-term survival benefits conferred by nitrogen deprivation (Fig. 6o). Similarly, a temperature-sensitive mutation of eIF5A (*hyp2-1*) entirely abolished the beneficial effect of nitrogen deprivation on chronological lifespan at the restrictive temperature (28 °C), when eIF5A^H^ was blocked (Supplementary Fig. 6a,b), but only partially at the permissive temperature (20 °C) (Supplementary Fig. 6c). Moreover, GC7 reduced eIF5A^H^ (Supplementary Fig. 6d,e) and chronological lifespan extension by nitrogen depletion (Supplementary Fig. 6f,g).

A heterozygous point mutation in eIF5A mutating lysine 51, which is the target of hypusination in flies (*eIF5A*^*K51R*^), has previously been shown to render SPD supplementation ineffective on the climbing ability of aging flies^53^. Likewise, IF^12:12^ conferred lifespan extension was lost in female and male *eIF5A*^*K51R*/+^ flies (Supplementary Fig. 6h,i), while not affecting food consumption during the first cycles of IF^12:12^ (Supplementary Fig. 6j). Similarly to SPD supplementation^53^, IF^12:12^ did not improve the climbing ability of aged female *eIF5A*^*K51R*/+^ flies (Supplementary Fig. 6k–m).

**Extended Data Fig. 10:**
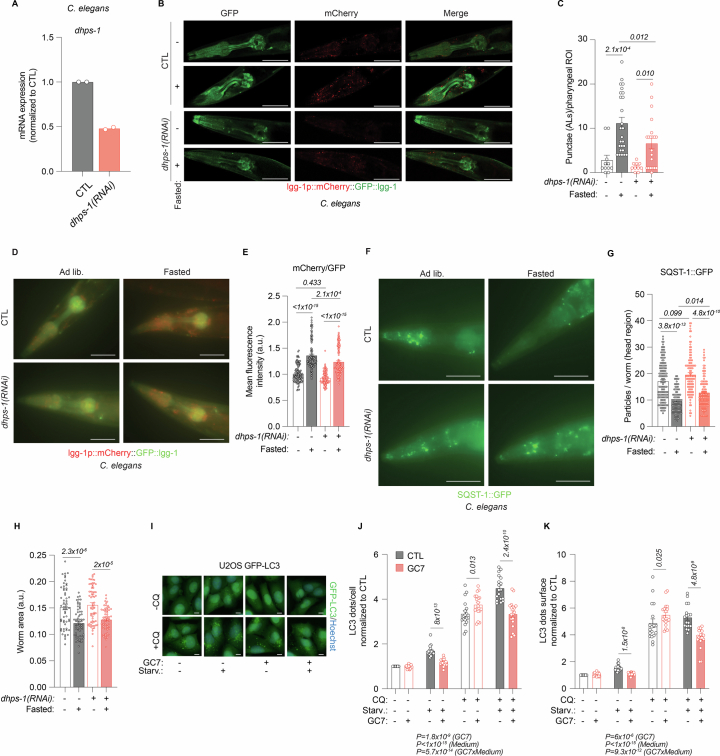
Hypusination is important for fasting-induced autophagy in worms and human cells. **(a)** Feeding of *dhps-1(RNAi)* expressing bacteria reduces *dhps-1* mRNA expression in *C. elegans* N2 worms. N = 2 biologically independent experiments. **(b)** Representative confocal images of the head region of young *C. elegans* MAH215 (sqIs11 [lgg-1p::mCherry::GFP::lgg-1 + rol-6]) fasted for two days and fed control or *dhps-1(RNAi)* expressing bacteria. Autolysosomes (ALs) appear as mCherry-positive puncta. Scale bar = 50 μm. **(c)** Quantification of ALs as depicted in [B]. Note that the experiment and statistics were performed together with the *odc-1(RNAi)* groups in Fig. 3p. N = 11(CTL ad lib), 26(CTL fasted), 10(*dhps-1(RNAi)* ad lib), 21(*dhps-1(RNAi)* fasted) worms. **(d)** Representative fluorescence images of the head region of young *C. elegans* MAH215 (sqIs11 [lgg-1p::mCherry::GFP::lgg-1 + rol-6]) (LGG-1 is the *C. elegans* ortholog of LGG-1/Atg8) fasted for two days and fed control or *dhps-1(RNAi)* expressing bacteria. Autophagic activity is indicated by a shift to the red spectrum due to fluorescence quenching of the pH-sensitive-GFP by the acidic environment of the autolysosome. Scale bar = 50 μm. **(e)** Quantification of the ratio of the mean fluorescence intensity of mCherry/GFP signals, as depicted in [D]. Note that the experiment and statistics were performed together with the *odc-1(RNAi)* groups in Extended Data Fig. 6d. N = 121(CTL ad lib), 127(CTL fasted), 107(*Dhps-1(RNAi)* ad lib), 143(*Dhps-1(RNAi)* fasted) worms. **(f)** Representative fluorescence images of the head region of young *C. elegans* SQST-1::GFP fasted for two days and fed control or *dhps-1(RNAi)* expressing bacteria. Autophagic activity is indicated by a decrease in the number of GFP-positive particles. Scale bar = 50 μm. **(g)** Quantification of the SQST-1::GFP particles in the head region, as depicted in [F]. Note that the experiment and statistics were performed together with the *odc-1(RNAi)* groups in Extended Data Fig. 6f. N = 133(CTL ad lib), 86(CTL fasted), 108(*dhps-1(RNAi)* ad lib), 100(*dhps-1(RNAi)* fasted) worms. **(h)** Knockdown of *dhps-1* does not affect worm size under ad lib or IF conditions. Note that the experiment and statistics were performed together with the *odc-1(RNAi)* groups in Extended Data Fig. 7g. N = 56(CTL ad lib), 66(CTL IF), 62(*dhps-1(RNAi)* ad lib), 59(*dhps-1(RNAi)* IF) worms. **(i)** Representative images of U2OS GFP-LC3 cells starved in HBSS with or without 100 µM GC7 for 6 hours. CQ was added for 3 hours before fixation. **(j-k)** Quantifications of [I]. N = 18 biologically independent samples. Statistics: [C,E,G] Kruskal-Wallis-test with Dunn’s correction. [H,J,K] Two-way ANOVA with Holm-Šídák’s multiple comparisons test. Bar graphs show the mean ± s.e.m. Source numerical data are available in source data. Source data

In *C.* *elegans*, the knockdown of *dhps-1* (Extended Data Fig. 10a) attenuated the induction of autophagy (Extended Data Fig. 10b–g) and strongly reduced the beneficial effect of IF^48:48^ on lifespan (Fig. 6p) without affecting body size (Extended Data Fig. 10h). Similarly, GC7 reduced autophagic flux in starved U2OS cells (Extended Data Fig. 10i–k).

In summary, fasting induced SPD-dependent increases in eIF5A^H^ in multiple species and this effect on eIF5A^H^ was required for the pro-longevity effects of nitrogen starvation or IF in yeast, worms and flies.

## Discussion

Acute fasting stimulates autophagy by inhibiting nutrient sensors (such as TORC1 and EP300) and activating signal transducers for nutrient scarcity (such as AMPK and SIRT1)^4,57^. Adult-onset IF with CR represents a translatable tool to improve age-associated diseases and systemic health of humans. Still, mechanistic details into the molecular and metabolic relay of nutritional information to lifespan regulation are missing but mandatory for successful clinical implementation. Here, we focused on closing a major gap in our understanding of the metabolic control of fasting-induced autophagy and longevity that apparently involves an increase in SPD-dependent hypusination of eIF5A.

Although fasting reduces the levels of amino acids, including ARG (together with its product ORN) and MET (and its product SAM), it also stimulates metabolic flux through the polyamine synthesis pathway, favouring an increase in SPD levels across different species, which requires ODC1. SPD is needed to hypusinate and activate eIF5A, a translation factor known to stimulate autophagy^58^. Indeed, we detected fasting-induced eIF5A hypusination across all analysed species, supporting the concept that SPD is universally implicated in the fasting response. Accordingly, genetic or pharmacological inhibition of SPD elevation or eIF5A hypusination curbed autophagy induction by fasting, and the longevity-promoting, cardioprotective and antiarthritic effects of IF across the phylogenetic spectrum.

Among the three ODC1-dependent polyamines, SPD seems to be crucial for mediating many fasting responses, mainly because it represents the sole co-factor for eIF5A hypusination. Nevertheless, supplementation with PUT or SPM also reversed ODC1 deficiencies, likely because they are interconvertible with SPD. However, PUT and SPM may partly function by other, yet-to-be-identified mechanisms.

We also found that autophagy induction by pharmacological mTOR inhibition partly depends on SPD synthesis in yeast and human cells, arguing in favour of a general role of SPD in autophagy stimulation. Notably, in yeast, nitrogen deprivation-induced inhibition of TORC1 was partially delayed in ∆*spe1* cells, indicating a reciprocal relationship between polyamines and TOR signalling. Reflecting a complex crosstalk between polyamines and mTOR, SPD treatment was previously shown to activate mTORC1 in the white adipose tissue (WAT) of young mice, but to inhibit mTORC1 in the WAT of aged mice fed a high-fat diet, and not to affect liver mTOR signalling^59^, suggesting organ- or cell type-specific circuitries that remain to be explored.

Of note, in long-lived *dilp2-3,5* mutant flies (which lack three of the seven insulin-like peptides), autophagy is induced as a result of enhanced levels of glycine *N*-methyltransferase (GNMT), which also results in enhanced synthesis of the pro-autophagic metabolite SPD^60^. GNMT overexpression is sufficient to increase lifespan in flies, and lifespan extension via dietary restriction partially depends on GNMT in flies^61^. Similarly, *ODC-1* is highly upregulated in the well-studied long-lived *C.* *elegans daf-2* mutant^62^, which encodes for the IGF1 receptor^63,64^, further suggesting that the polyamine and insulin signalling pathways are intertwined. Moreover, the liver-specific knockout of insulin receptor substrate 1 (IRS1) causes an increase in GNMT levels in mice^60^, pointing to a phylogenetically conserved pathway linking reduced trophic signalling to SPD elevation. However, it remains to be determined whether this pathway is active in response to fasting. Recently, the longevity effects of IF were linked to the circadian regulation of autophagy in flies^65^. As polyamines are subject to, and regulate, circadian rhythms^66^, this adds yet another possible intersection of lifespan regulation by SPD and hypusination in the context of fasting regimes.

The exact kinetics of the polyamine-eIF5A^H^ response to IF or CR remains unclear on a cell- and tissue-specific level. In future long-term studies, it will be important to weigh the impact of fasting length/periodicity versus the level of CR on polyamine metabolism. In rodents and humans, isocaloric IF studies have produced mixed results on health outcomes and lifespan and it remains to be studied how polyamines mechanistically integrate into this complexity. Given the observation that serum SPD levels were increased in mice after eight months of CR, it will be important to determine how pharmacological or genetic inhibition of polyamine metabolism affects the extension of healthspan and lifespan conferred by CR rather than by IF. While our work focused on the effects downstream of polyamine metabolism, additional studies should dissect the molecular relays connecting nutrient status to the transcriptional, translational and post-translational regulation of polyamine metabolism and spermidine-dependent eIF5A hypusination. Currently, the biochemical mechanism through which IF and CR stimulate polyamine synthesis and subsequent eIF5A hypusination remain elusive, limiting the novelty of our study. Thus, future work must elucidate the crosstalk between polyamines and nutrient-responsive factors, such as insulin/IGF1 and mTOR that inhibit autophagy and accelerate aging or AMPK that induces autophagy and favourably influences healthspan and lifespan. It will also be important to determine whether polyamines induced by IF always confer health benefits. While oral spermidine supplementation can elicit anticancer effects^67,68^, elevated polyamine levels are detected in many cancer types and may stimulate cellular proliferation^69^. Thus, the impact of IF on patients with cancer remains to be carefully evaluated.

Our study reveals that fasting-induced longevity and improved healthspan partially rely on SPD-dependent eIF5A hypusination and ensuing autophagy induction in multiple species.

## Methods

All experiments were conducted in compliance with relevant ethical regulations, as indicated in the respective sections.

### Yeast

#### Strains

BY4741 (*MATa his3*Δ*1 leu2*Δ*0 met15*Δ*0 ura3*Δ*0*) was used for all experiments, except for experiments with rapamycin in which we used BY4742 (*MATα his3*Δ*1 leu2*Δ*0 lys2*Δ*0 ura3*Δ*0*). The null mutants BY4741 ∆*spe2*::kanMX (Y01743), ∆*spe3*::kanMX (Y05488), ∆*spe4*::kanMX (Y06945) and the temperature-sensitive mutant *hyp2-1*:kanMX (TSA736) were obtained from Euroscarf. We generated the null mutants BY4741 ∆*spe1::*hphNT1, ∆*lia1*::natNT2 and ∆*spe1::*hphNT1∆*lia1*::natNT2 according to previously described methods^70^. In brief, we amplified a gene-specific hphNT1 or natNT2 knockout cassette by PCR using the template plasmids pFA6a-hphNT1 and pFA6a-natNT2 (ref. ^70^) and primers:

SPE1_pYM_S1: 5′-GTTCTACAACTTTTTCATAGTAATCAAAACCTTTGAATTTCAAACTTACTATGCGTACGCTGCAGGTCGAC-3′

SPE1_pYM_S2: 5′-CTTTTCCCACCCCCTCCGTCTCTCTTGCGAAAGTCGTGGTTAAATATATCCTTCAATCGATGAATTCGAGCTCG-3′

Forward control primer SPE1_Ctrl: 5′-TCATCAAGAGCCCCATCC-3′

Reverse control primer Control_S1: 5′-GTCGACCTGCAGCGTACG-3′

LIA1_pYM_S1: 5′-GTTAGGATAAACTGTAGTCCTTCTAACATACCACGCAAGAAAGAAAAAAAAAAACCGTACGCTGCAGGTCGAC-3′

LIA1_pYM_S2: 5′-CAAGATTATACAATGATTATTGTTACTATCATTATTGATGATGCTGATTCTTATCGATGAATTCGAGCTCG-3′

Forward control primer LIA1_Ctrl: 5′-GTTCCCAGGCGAAGAAAGAAC-3′

Reverse control primer natNT2_Ctrl: 5′-CGTGTCGTCAAGAGTGGTAC-3′

To determine the protein levels of Dys1 and Lia1, we generated genome-tagged C-terminal 6×HA fusions using the plasmid pYM16 (ref. ^70^) and the following primers:

For Dys1-6×HA:

DYS1_pYM_S3: 5′-CTGCTACCTTTGCCAGTGGTAAACCAATCAAAAAAGTTAAGAATCGTACGCTGCAGGTCGAC-3′

DYS1_pYM_S2: 5′-GAAATAGTACAGATTCATTTTTTTTTTTTTCATCTCAAAATTCTCTCATCAATCGATGAATTCGAGCTCG-3′

Forward control primer DYS1_Ctrl: 5′-GCGTGACCAAGGTATGAATCGTATT-3′

Reverse control primer Control_hphNT1: 5′-CATATCCACGCCCTCCTAC -3′

For Lia1-6×HA:

LIA1_pYM_S3: 5′-GATATGTATGATTACGAAAACAGCAACGAACTAGAATATGCTCCAACTGCTAATCGTACGCTGCAGGTCGAC-3′

LIA1_pYM_S2: 5′-CAAGATTATACAATGATTATTGTTACTATCATTATTGATGATGCTGATTCTTCTAATCGATGAATTCGAGCTCG-3′

Forward control primer LIA1_Ctrl: 5′-CTAGGTGACAAGGATTCGTTGGATG-3′

Reverse control primer Control_hphNT1: 5′- CATATCCACGCCCTCCTAC-3′

For the Pho8∆N60 assay to measure nonselective autophagy, we generated *PHO8 pho8∆N60-URA3* strains in BY4741 WT and ∆*spe1* background (in this case, the ∆*spe1* strain was obtained from Euroscarf*; spe1*::kanMX; Y05034) using the integrative pTN9-URA plasmid^71^. Pho8∆N60-specific alkaline phosphatase activity was determined as described previously^72^. Endogenous GFP–Atg8 fusions were generated as previously described^72^. In brief, we used the vector template pYM-pATG8 (ref. ^72^) to construct *NatNT2::P*_*ATG8*_*-yeGFP-ATG8* strains with primers:

Atg8_pYM_S1: 5′-CTAATAATTGTAAAGTTGAGAAAATCATAATAAAATAATTACTAGAGACATGCGTACGCTGCAGGTCGAC-3′

Atg8_pYM_S4: 5′-GACTCCGCCTTCCTTTTTTCAAATGGATATTCAGACTTAAATGTAGACTTCATCGATGAATTCTCTGTCG-3′

Forward control primer yeGFP_F: 5′-GGTGAAGAATTATTCACTGGTGTTG-3′

Reverse control primer ATG8_R: 5′-GAACAATAGATGGCTAATGAGTCC-3′

#### Medium and growth conditions

All experiments were performed in baffled 100-ml flasks filled with 10–15 ml growth medium, incubated at 28 °C and constant shaking (145 rpm), if not stated otherwise. For general experiments, we used standard dextrose medium (CTL) containing 0.17% yeast nitrogen base (BD Diagnostics), 0.5% (NH_4_)_2_SO_4_, 80 mg l^−1^ histidine, 200 mg l^−1^ leucine 320 mg l^−1^ uracil, 30 mg l^−1^ adenine and 30 mg l^−1^ all other amino acids, with 2% glucose, which we sterilized as separate 10× stocks by autoclaving. For experiments with BY4742 we additionally added 90 mg l^−1^ lysine after mixing the stocks.

All cultures were inoculated from cellular material from a YPD (1% yeast extract (BD), 2% bacto peptone (BD), 2% glucose, 2% agar) plate, which was incubated at 28 °C for 2–3 days, placed at 4 °C for at least 1 day and a maximum of 2 weeks. We used pre-cultures in CTL medium to inoculate main cultures to an OD_600_ of ~0.01–0.05. In general, main cultures were grown to mid-logarithmic phase (OD_600_ = 1), centrifuged at 3,000–4,000 rpm (1731–3077 rcf) for 3–5 min, washed once with sterile pre-warmed ddH_2_O, and re-suspended in fresh medium. For nitrogen deprivation (−N) we re-suspended the cells in fresh CTL or −N medium, containing 0.17% yeast nitrogen base and 2% glucose. For glucose restriction, we used CTL medium with either 0.5% or 0.05% glucose. For water starvation, we used sterile ddH_2_O.

#### Rapamycin and GC7 treatments

Rapamycin (LC Laboratories, R-5000) stock was prepared as 1.1 mM in dimethylsulfoxide (DMSO) (stored at −20 °C) and used at a final concentration of 40 nM. Analogous to the −N experiments, we initiated rapamycin treatment by adding DMSO as a control or rapamycin to the cultures during the mid-logarithmic phase (at OD_600_ = 1). The pharmacological DHS inhibitor N1-guanyl-1,7-diamine-heptane (GC7) was purchased from Merck (259545) and prepared as a sterile filtered 50 mM stock in ddH_2_O, stored at −20 °C. GC7 treatment was initiated during inoculation and repeated when the medium was changed for nitrogen deprivation.

#### Yeast polyamine supplementation

Generally, polyamines were supplemented from sterile stocks of polyamines starting with the main culture, except for synergy experiments as shown in Fig. 3k,l, Supplementary Fig. 4c and Extended Data Figs. 4m,n and 9e,f, where spermidine was added in the starvation medium only. For the other nitrogen starvation experiments, polyamine supplementation was renewed in the fresh medium. Cells were treated with either 100 µM putrescine (Sigma, D13208; from 3 M aqueous stock, pH 7.4), spermidine (Sigma, 85558; from 1 M aqueous stock, pH 7.4) or spermine (Sigma, 85590; from 0.9 M aqueous stock, pH 7.4) or 5 mM spermidine as indicated in the figure legends. In brief, the polyamine stocks were prepared on ice, with ddH_2_O and the pH was set with HCl, as previously described^23^. Stock solutions were stored at −20 °C for up to one month.

#### Yeast autophagy measurements

To monitor yeast autophagy, we employed established protocols using fluorescence microscopy, western blots and biochemical assays^73^. We used endogenously tagged eGFP–Atg8 fusion strains, as previously reported^72^.

GFP–Atg8 localization was assessed by collecting 100 µl from yeast cultures by centrifugation (1,000 rcf, 1 min) and re-suspension of the cell pellet in 50 µl propidium iodide (PI) staining solution (100 ng ml^−1^ PI in PBS, pH 7.4). Cells were pelleted again (500 rcf, 30 s), transferred to a glass microscopy slide, and covered with a cover slip. Imaging was performed with a Leica DM6B-Z fluorescence microscope using a ×100/1.40 HC Pl APO oil objective and a Leica-DFC9000GT-VSC09095 sCMOS camera. eGFP images were taken with a GFP filter (Ex_470_/Em_525_) filter and 2 s exposure. PI images were taken with a Texas Red filter (Ex_560_/Em_630_) and 75-ms exposure. Raw 16-bit images were exported and merged with constant contrast settings (eGFP 3,000–30,000; PI 200–50,000) in ImageJ^74^. Cells were classified manually in a blinded fashion using the ImageJ CellCounter tool.

Additionally, we performed Pho8∆N60 assays^43^, as previously reported^72^. In brief, we collected three OD_600_ equivalents at the indicated time points by full-speed centrifugation in a standard tabletop centrifuge for 2 min. After a washing step with ddH_2_O, we re-suspended the cell pellet in 200 µl cold assay buffer (250 mM Tris-HCl (pH 9), 10 mM MgSO_4_ and 10 μM ZnSO_4_) and transferred it to pre-cooled plastic reaction tubes filled with 100 µl acid-washed glass beads. The cells were homogenized in a BeadBeater (2 × 1 min with 1-min pause between the cycles) in a liquid nitrogen-cooled metal rack. After homogenization we centrifuged the samples at 10,000 rcf for 10 min at 4 °C and 100 µl supernatant was transferred into a fresh pre-cooled tube. We used a Bio-Rad protein assay (Bio-Rad, 5000006) to determine the protein concentration of the supernatant. Protein extract corresponding to 1.5 µg protein was transferred to a well of a 96-deep-well plate in duplicate and filled with assay buffer to 550 µl at room temperature (22–25 °C). The addition of 50 µl α-naphthyl phosphate solution (55 mM in assay buffer, pH 9) started the reaction. After mixing the plate by vortexing, it was incubated for 20 min at 30 °C. The reaction was stopped by adding 200 μl stopping buffer (2 M glycine/NaOH, pH 11) to each well, followed by vortexing. Then, 100 µl of each well was transferred to a 96-well plate (black bottom) in duplicate and measured in a TECAN plate reader (Ex_340_ and Em_485_). For the correction of background phosphatase activity, control strains (without the Pho8∆N60 mutation) were processed in parallel and subtracted.

#### Yeast immunoblotting

A culture volume corresponding to three OD_600_ units was collected at each time point by centrifugation at 3,000 rcf for 3 min. After a washing step with ddH_2_O, the cell pellet was frozen at −20 °C until further processing. Whole-cell extracts were generated by re-suspending the cell pellets in 300 µl lysis buffer (1.85 M NaOH and 7.5% 2-mercaptoethanol) and incubation on ice for 10 min. Proteins were precipitated by adding 300 µl 55% trichloroacetic acid (TCA), incubation on ice for 10 min, centrifugation at 10,000 rcf at 4 °C for 10 min and removal of the supernatant. After an additional centrifugation step (10,000 rcf at 4 °C for 2 min) residual supernatant was removed and pellets were solubilized in final sample buffer (62.5 mM Tris-HCl, pH 6.8, 2% SDS, 8.7% glycerol, 0.004% bromophenol blue and 120 mM dithiothreitol (DTT)) and 1 M Tris was added to neutralize residual TCA until the samples turned blue. In brief, before electrophoresis, samples were boiled at 95 °C for 5 min and centrifuged at maximum speed in a tabletop centrifuge for 15 s. For protein separation, 12–15 µl of the supernatant was loaded on 4–12% or 12% (for GFP–Atg8) NuPAGE Bis-Tris gels (Thermo Fisher Scientific). Electrophoresis was performed at 100–130 V with MOPS SDS running buffer (Thermo Fisher Scientific, NP000102). Proteins were wet-transferred to methanol-activated 0.45-µm PVDF membranes (Roth, T830.1) at 220 mA for 60–90 min using transfer buffer (10 mM CAPS/NaOH, pH 11 and 10% methanol). After blotting, membranes were blocked with blocking solution (1% dry milk powder in Tris-buffered saline (TBS), pH 7.4) for 1 h and then incubated with the primary antibodies overnight at 4 °C. After three washing steps in TBS + 0.1% Triton X-100 for 5 min, membranes were incubated with secondary, horse radish peroxidase (HRP)-linked antibodies for 1 h at room temperature. After three washing steps in TBS + 0.1% Triton X-100 for 5 min, signals were detected with a ChemiDoc detection system (Bio-Rad) and Clarity Western ECL Substrate (Bio-Rad) using the ‘optimal exposure’ setting. For re-probing membranes, Restore PLUS Western Blot Stripping Buffer (Thermo Fisher Scientific, 46430) was used according to the manufacturer’s protocol. Band intensities were quantified using ImageLab v.5.2 (Bio-Rad) using the rectangular volume tool with local background adjustment. Primary antibodies were anti-GFP (Roche, 1814460, 1:5,000 dilution in blocking solution), anti-hypusine (Merck, ABS1064-I, 1:1,000 dilution in blocking solution), anti-GAPDH clone GA1R (Thermo Fisher Scientific, MA5-15738, 1:10,000 dilution in blocking solution) and anti-HA (Sigma, H-9658, 1:10,000 dilution in blocking solution). Secondary antibodies were HRP-linked anti-mouse IgG (Sigma, A9044, 1:10,000 in blocking solution) or HRP-linked anti-rabbit IgG (Sigma, A0545, 1:10,000 dilution in blocking solution).

#### Yeast chronological lifespan

Chronological aging experiments were performed as previously described^75^. In brief, we used PI staining to identify dead cells using an LSR II Fortessa flow cytometer equipped with a high-throughput sampler (BD), using FACSDiva software (BD). For that purpose, 20-µl aliquots of the cultures were transferred to 96-well U-bottom plates at the indicated time points and stained with 150 µl PI staining solution (100 ng ml^−1^ in PBS), incubated in the dark for 15 min at room temperature and centrifuged at 2,500 rcf for 5 min. The staining solution was removed by tapping the plate and the cells were re-suspended in 150 µl fresh PBS. Then, 30,000 cells were measured per sample and PI-positive (dead)/negative (live) cells were identified and quantified by flow cytometry at Ex_488_/Em_670_.

At selected time points, we additionally performed live qualitative fluorescence microscopy, for which we took 100-µl aliquots of the cultures, centrifuged them for 1 min at room temperature in a standard tabletop centrifuge and re-suspended the pellet in 100 µl PI staining solution. After 2 min of incubation in the dark, we transferred a droplet of this suspension onto glass microscopy slides and covered them with coverslips. Images were taken with a Leica DM6B-Z microscope, using the Texas Red filter (Ex_560_/Em630, 300-ms exposure). For image processing, we used ImageJ^74^.

Of note, yeast chronological lifespan is affected by the medium pH^76^, which was measured with a Thermo Scientific Orion Star A221 pH meter and only minimally affected during nitrogen starvation by the *SPE1* knockout (Supplementary Fig. 4a).

#### Yeast replicative lifespan

Yeast replicative lifespan was performed as previously described^77,78^. To summarize, yeast was pulled from −80 °C freezer stocks onto YPD plates and were allowed to grow at 30 °C for 2 days. Single colonies from each sample were picked and patched for two consecutive days before being plated to RLS assay plates (YPD (1% yeast extract, 2% peptone and 2%, 0.5% or 0.05% glucose) unless otherwise stated). Virgin cells were selected as new mother cells and each generation of daughter cells were separated and counted via microdissection. Cell death is considered when mother cells are no longer capable of dividing. Mother cells that divided two or fewer times or were lost during the experiment were censored from the dataset.

#### Yeast TORC1 activity

In vivo TORC1 activity was assayed as previously described^79^. In brief, a 10-ml cell culture was mixed with TCA at a final concentration of 6%. After centrifugation, the pellet was washed with cold acetone and dried in a SpeedVac. The pellet was re-suspended in lysis buffer (50 mM Tris-HCl, pH 7.5, 5 mM EDTA, 6 M urea and 1% SDS), the amount being proportional to the OD_600_ of the original cell culture. Proteins were extracted by mechanical disruption in a Precellys machine after the addition of glass beads. Subsequently, an amount of 2× sample buffer (350 mM Tris-HCl (pH 6.8), 30% glycerol, 600 mM DTT, 10% SDS and BBF) was added to the whole-cell extract and the mix was boiled at 98 °C for 5 min. The analysis was carried out by SDS–PAGE by loading 15 µl extracts on 7.5% polyacrylamide gels. The transfer on nitrocellulose membranes was performed with a Trans-Blot Turbo Transfer System (Bio-Rad) with Bjerrum Schafer-Nielsen buffer (48 mM Tris-HCl (pH 9.2), 39 mM glycine and 20% methanol). Protein detection was carried out using custom phosphospecific rabbit anti-Sch9-pThr737 (1:10,000 dilution in TBS 1% milk) and goat anti-Sch9 (1:1,000 dilution in TBS 5% milk) antibodies and a commercial rabbit anti-Adh1 antibody (Calbiochem, 126745, 1:50,000 dilution in TBS 5% milk). Band intensities from independent biological replicates were quantified with ImageJ.

#### Yeast metabolomics

Cells (equivalent to 15 OD_600_, grown as described before) of BY4741 and BY4741 ∆*spe1* after 6 or 24 h of nitrogen deprivation were collected by centrifugation at 4,000 rpm (3,077 rcf) for 3 min at 4 °C, washed once with ice-cold ddH_2_O, frozen in liquid nitrogen and stored at −80 °C until further processing. The pellets were re-suspended in 400 µl ice-cold methanol (−20 °C) and 200 µl MilliQ H_2_O and transferred to a tube containing Precellys beads (1.4-mm zirconium oxide beads, Bertin Technologies, Villeurbanne, France) for homogenization on a Precellys 24 homogenizer for two cycles of 20 s with 5,000 rpm and 10 s breaks. Cell debris was pelleted by centrifugation at 13,000 rpm (15,871 rcf) for 30 min (4 °C). Supernatants were lyophilized and dissolved in 500 µl NMR buffer (0.08 M Na_2_HPO_4_, 5 mM 3-(trimethylsilyl) propionic acid-2,2,3,3-d_4_ sodium salt (TSP), 0.04% (*w*/*v*) NaN_3_ in D_2_O, pH adjusted to 7.4 with 8 M HCl and 5 M NaOH) for NMR measurements.

All NMR experiments were acquired at 310 K using a Bruker 600 MHz Avance Neo spectrometer equipped with a TXI probe head. The one-dimensional (1D) CPMG (Carr–Purcell–Meiboom–Gill) pulse sequence (‘cpmgpr1d’; 512 scans; size of free induction decay (FID) 73,728; 11,904.76 Hz spectral width; recycle delay 4 s), with water signal suppression using presaturation, was recorded for ^1^H 1D NMR experiments. In brief, and as described before^80^, data were processed in Bruker Topspin v.4.0.6 using one-dimensional exponential window multiplication of the FID, Fourier transformation and phase correction. The NMR data were then imported into Matlab2014b; TSP was used as the internal standard for chemical-shift referencing (set to 0 ppm); regions around the water, TSP and methanol signals were excluded; the NMR spectra were aligned; and a probabilistic quotient normalization was performed. The statistical significance of the determined differences was validated by the quality assessment statistic Q^2^. This measure provides information about cross-validation and is a qualitative measure of consistency between the predicted and original data with a maximum value of 1.

Metabolite identification was carried out using Chenomx NMR Suite v.8.4 (Chenomx) and reference compounds. Quantification of metabolites was carried out by signal integration of normalized spectra. For each metabolite, a representative peak with no overlapping signals was identified, the start and end points of the integration were chosen to revolve around that peak, and the area of the peak was integrated by summing up the value for each point. Peaks which could not be unambiguously identified are shown with their ppm value at maximal intensity.

We used MetaboAnalyst v.5.0 for data analysis and visualization^81^, using mean-centred normalization. For metabolite set enrichment analysis we used the integrated KEGG database. sPLS-DA analysis was performed with ten features for each component and heatmaps were generated using Ward’s method and Euclidian distance settings.

#### Yeast polyamine measurements

Polyamine extraction and quantification was performed as previously described^82^. In brief, yeast cells were grown as previously mentioned and at the indicated time points, one to three OD_600_ were collected by centrifugation, washed once with ddH_2_O and mixed with 5% TCA containing stable-isotope labelled standards of indicated polyamines at a final concentration of 100 ng ml^−1^ each, followed by incubation on ice for 60 min with vortexing every 15 min. After centrifugation at 25,000 rcf at 4 °C for 10 min, 150 µl supernatant were transferred to 1.5-ml LoBind reaction tubes and 37.5 µl ammonium formate (2 M), 800 µl of ultra-pure H_2_O and 125 µl of saturated Na_2_CO_3_ buffer were added.

For derivatization, 20 µl isobutyl chloroformate was added, followed by a 15-min incubation at 35 °C. After centrifugation for 1 min at 15,000 rpm/21,130 rcf, 800 µl supernatant was then transferred to LoBind 96-deep-well plates (VWR International, 737-2544) for storage at −80 °C. Polyamine derivatives were extracted offline by solid-phase extraction (SPE) (Strata-X, Polymeric Reversed Phase, 96-well plate). SPE was conditioned with 500 µl acetonitrile, equilibrated with 500 µl distilled water containing 0.2% acetic acid. Derivatized TCA extracts were loaded onto the SPE and after two washing steps with 500 µl 0.2% acetic acid, samples were eluted with 250 µl 80% acetonitrile containing 0.2% acetic acid. Eluted SPE extracts were subjected to liquid chromatography (LC)–MS/MS (mobile phase, isocratic 80% acetonitrile containing 0.2% acetic acid; flow rate 250 µl min^−1^; HPLC column: Kinetex 2.6 µm C18 100A 50 mm × 2.1 mm TSQ Quantum Access Max coupled to an Ultimate 3000). MS conditions were set as previously published^83^. LC–MS/MS data were acquired and processed using Xcalibur v.4.0 Software (Thermo Fisher Scientific). The final data were normalized to OD_600_ and the control conditions for each polyamine.

#### Yeast arginine flux metabolomics

The main cultures were grown to log phase in medium containing ^13^C_6_-labelled arginine (30 mg l^−1^; Eurisotop, CLM-2265) instead of non-labelled arginine. The control cells received fresh medium containing ^13^C_6_-labelled arginine, whereas the nitrogen-starved cells were cultivated in standard −N medium for another 6 h, before collecting by centrifugation as described above. Metabolites were measured following a previously described protocol^84^, but without internal standards in the extraction buffer. In brief, 50-µl samples were vortexed for 5 min with 500 µl ice-cold extraction mixture (methanol:water, 9:1, −20 °C) and then centrifuged (10 min at 15,000 rpm/21,130 rcf, 4 °C). Several fractions were then split to be analysed by LC–MS and gas chromatography (GC)–MS^84^. One fraction was analysed by the LC–MS Orbitrap q-Exactive Thermo in full profiling. Tracking of arginine and its isotopes was performed by post-acquisition treatment with the software Xcalibur Thermo (v.2.2). Polyamines analyses were performed by LC–MS/MS with a 1290 UHPLC (ultra-high performance liquid chromatography) (Agilent Technologies) coupled to a QQQ 6470 (Agilent Technologies) and were previously described^85^. The MRM method was adapted to track polyamines but also their isotopes. Data were processed by MassHunter Quantifier (Agilent) software (v.10.1).

#### Yeast proteomics

Yeast samples were processed in each sequence (experiment) in one MS run to reduce batch effects. For cell lysis 5% SDS, 50 mM ammonium bicarbonate (used in the GC7-pertubation experiment) or 8 M urea, 20 mM HEPES (used in the SPD-rescue experiment) and zirconia beads (1:1 *v*/*v*) were added to the frozen pellet and lysed in a BeadBeater in three cycles (2 min, 15 s rest, 1,500 rpm per cycle) (Spex Geno Grinder). Then, 150 µg protein of each sample were alkylated with 5 mM tris(2-carboxyethyl)phosphine (TCEP), reduced with 10 mM chloroacetamide (CAA) and digested with trypsin (1:50 *wt*/*wt*) as well as purified utilizing the S-Trap mini columns (Protifi), following the manufacturer’s instructions: S-Trap mini spin column digestion protocol. Peptides were lyophilized, re-suspended in 0.1% formic acid and diluted to 200 ng µl^−1^, whereby 1 µl was used per MS injection.

Samples were analysed on a timsTOF ion mobility mass spectrometer (with PASEF technology, Bruker Daltonics) in-line with UltiMate 3000 RSLCnano UHPLC system (Thermo Scientific). Peptides were separated on a reversed-phase C18 Aurora column (25 cm × 75 µm) with an integrated Captive Spray Emitter (IonOpticks). Mobile phases A 0.1% (*v*/*v*) formic acid in water and B 0.1% (*v*/*v*) formic acid in ACN with a flow rate of 300 nl min^−1^, respectively. Fraction B was linearly increased from 2% to 25% in the 90-min gradient, increased to 40% for 10 min, and a further increase to 80% for 10 min, followed by re-equilibration. The spectra were recorded in data-independent acquisition (DIA) mode as previously described^86^.

The DIA data were quantified with DIA-NN v.1.8.0 (ref. ^87^) by creating a synthetic fasta library using the reviewed Uni-Prot *S.* *cerevisiae* protein database (downloaded 24 September 2021). The MS proteomics raw data of both experiments together with the processing log files have been deposited to the ProteomeXchange Consortium using the PRIDE partner repository (https://www.ebi.ac.uk/pride/) with the dataset identifier PXD035909. In the SPD-rescue experiment, a total of 4,684 distinct protein groups were identified. Notably, 82% of these groups demonstrated consistent presence across all samples, indicating a robust and pervasive occurrence. Furthermore, close to 90% of the samples exhibited the presence of approximately 4,207 protein groups, corroborating their widespread and persistent detection within the experimental set. In the GC7-perturbation experiment, 4,019 unique protein groups were quantified. Among these, 45% were identified across every sample, with approximately 58% detected in at least 90% of the samples.

PCA was performed by singular-value decomposition of the centred and scaled protein groups (*n* = 4,684), whereby missing values were imputed with zero. Protein complex enrichment analyses were performed based on KEGG yeast pathways from ConsensusPathDB (downloaded 27 April 2022)^88^, as well as complex portal annotations of the TORC complex^89^. Aggregated *z*-values were calculated for each protein complex resulting from averaged absolute protein expression of each complex member. The *z*-scores for each protein were calculated as followed $z=\frac{\left(x-\mu \right)}{\sigma }$, where *x* is the expression, *μ* is the protein expression across all samples and σ is the s.d. of the protein expression across all samples.

Data were visualized using R^90^ by utilizing the packages prcomp^91,92^, ggplot^93^ and pheatmap^94^.

### *Drosophila*

#### Fly strains, husbandry and food

All fly strains had a *w*^*1118*^ genetic background. The heterozygous mutant strains were backcrossed to the laboratory-specific *w*^*1118*^ at least six times to ensure isogenicity. The heterozygous *odc1* mutant was purchased from Bloomington Drosophila Stock center (Indiana University, 56103). The heterozygous eIF5A mutant carrying a point mutation at the lysine site of hypusination was created as described previously^53^. Fly food was prepared according to the Bloomington medium recipe with minor modifications, which we refer to as ‘normal food’ (per litre: 4.2 g agar–agar, 85.3 g sugar beet syrup, 7.5 g baker’s yeast, 8.3 g soybean flour, 66.7 g cornmeal, 1.3 g methyl p-hydroxybenzoate dissolved in 4.2 ml ethanol and 5.25 ml propionic acid). Difluoromethylornithine (DFMO) (a kind gift from P. M. Woster, Medical University of South Carolina), freshly prepared as a sterile filtered 50 mM stock in ddH_2_O, was added to the food after cooling the freshly cooked food to ~40 °C. Agar-only food (0.6%) prepared with a 1:1 mixture of deionized and tap water was used as the fasting food. The flies were reared under standard laboratory conditions, as previously reported (25 °C, 70% humidity with a constant 12-h light–dark cycle)^53,95^. Flies used in all experiments were F1. WT flies and mutant flies were collected within 24–48 h after eclosion and considered as 1-day-old flies. The *w*^*1118*^ virgin females were used to cross with males from *w*^*1118*^ and mutant strains, respectively. Female and male flies were separated after 24 h of mating on fresh food by light CO_2_ anaesthesia and were flipped to fresh normal food every other day in portions of 20–25 flies per vial.

#### Fly intermittent fasting

IF regimes were initiated 1 day after sorting and maintained throughout the flies’ lifespan. We used a 12-h feeding–fasting cycle (IF^12:12^), providing food during daylight hours (8:00 to 20:00). Control groups were flipped in the same rhythm but had food access during the whole 24 h. We changed food vials every 2–3 days and kept vials at 4 °C when not used. Flies that escaped or died due to unnatural causes were censored. Dead flies were identified by their unresponsiveness to mechanical stimuli using a small brush.

#### Fly body weight measurement

*Drosophila* body weight was measured throughout a fasting cycle as wet weight of snap-frozen fly bodies. The start time of the experiments was 20:00, followed by measurement time points after 12 and 24 h of fasting, as well as after 12 h of re-feeding. The ad libitum-fed group had access to food for the whole time. Data were normalized to each ad libitum group.

#### Fly food consumption

Food consumption was monitored similarly to the previously described capillary feeding (CAFE) assay^96^: For each replicate, five flies were anaesthetized on ice and transferred to a chamber with two 5-µl glass capillaries (VWR International, 612-2401) inserted in the top caps. Capillaries were either filled with liquid food containing 5% (*w*/*v*) sucrose (Roth, 4661.4) and 5% (*w*/*v*) yeast extract (BD, 288620) (ad libitum) or tap water (fasting). Data were collected twice per day (8:00 and 20:00) for at least 3 days after a 24-h acclimatization period. IF flies had access to capillaries containing food from 8:00 to 20:00 Passive evaporation was accounted for with three empty chambers without flies. Data are presented as microlitres of food per fly per hour.

#### Fly polyamine measurement

Twenty flies (wet weight of snap-frozen fly bodies was estimated and used for normalization) were placed into 1.5-ml LoBind reaction tubes (Eppendorf, 0030108116), snap-frozen in liquid nitrogen and homogenized for 30 s on ice in 600 µl 5% TCA containing stable-isotope labelled standards of indicated polyamines at a final concentration of 100 ng ml^−1^ each using a Turrax homogenizer (IKA T10 basic). Further polyamine extraction and quantification was performed as previously described with adapted analyte concentrations in the calibration solutions^82^ (see also ‘Yeast’ section).

#### Fly immunoblotting

For fly protein extraction, ten snap-frozen fly heads were homogenized in lysis buffer (1× PBS, pH 7, 0.5% Triton X-100, 2% SDS and 1× complete protease inhibitor mix) for 30 s with a motorized pestle (5 µl lysis buffer per fly head) followed by an incubation for 60 min at 4 °C with continuous rotation. The homogenized samples were centrifuged for 10 min at 10,000 rcf at 4 °C. The final sample buffer (final concentration of 62.5 mM Tris-HCl, pH 6.8, 2% SDS, 8.7% glycerol, 0.004% bromophenol blue and 120 mM DTT) was added to the supernatant and samples were stored at −20 °C until further processing. In brief, before electrophoresis, samples were boiled at 95 °C for 5 min and centrifuged at 10,000 rcf for 1 min. Then, 12.5 µl supernatant (equivalent to two heads) were loaded onto 4–12% Bis-Tris gels and run at 90–120 V. Proteins were transferred to 0.45-μm PVDF membranes for 90 min at 220 mA using transfer buffer (10 mM CAPS pH 11 and 10% methanol). The membranes were then incubated in blocking solution (3% dry milk powder in TBS, pH 7.4) for 1 h and subsequently incubated with the primary antibody overnight at 4 °C. Membranes were washed three times with TBST (TBS + 0.1% Tween-20) for 5 min and subsequently incubated with the secondary antibody for 1 h at room temperature. After three washing steps in TBST for 5 min, signals were detected with a ChemiDoc detection system (Bio-Rad) and Clarity Western ECL Substrate (Bio-Rad) using the ‘optimal exposure’ setting. Band intensities were quantified using ImageLab v.5.2 (Bio-Rad) using the rectangular volume tool with local background adjustment. Primary antibody was anti-hypusine (Merck, ABS1064-I, 1:1,000 dilution in 1% dry milk powder in TBS, pH 7.4). The secondary antibody was HRP-linked anti-rabbit IgG (Sigma, A0545, 1:10,000 dilution in 1% dry milk powder in TBS pH 7.4). As a loading control, HRP anti-β actin monoclonal antibody (Abcam, ab197277, 1:2,000 dilution in TBS + 0.1% Tween +1% BSA) was used.

#### Fly locomotor function (climbing assay)

For assessing the locomotor function, 5–20 flies were placed in custom-made three-dimensional-printed climbing chambers with nine slots per chamber followed by a defined acclimation period of 30 min in the dark. The chambers were individually attached to a custom-built climbing platform to ensure a standardized impact force and tapped down four times with an interval of 30 s (four rounds). Flies climbing up the rack were recorded by video tracking (Canon EOS 700D). The videos were then analysed with a fly tracking program, which tracks flies based on previously described methods^97^. For the calculation of climbing parameters (height, average height reached by the flies in the given climbing duration; speed, speed of the flies during the climbing duration in mm s^−1^; and distance, the distance travelled by the flies during the climbing duration), flies were tracked over 10 s after the tapping impact. For analysis, the area under the curve (AUC) was calculated for each parameter and strain and normalized to the respective control. Every data point corresponds to the average of the flies assessed in one chamber.

#### Fly whole-mount immunostaining, confocal imaging and quantification

Flies at 6 days old were either fed ad libitum or fasted for 12 or 24 h. Then the fly brains were dissected for brain immunostaining. Adult brains were dissected in HL3 solution on ice and immediately fixed in cold 4% paraformaldehyde (*w*/*v*) for 30 min at room temperature. After fixation, samples were washed three times for 10 min each with 0.7% PBT (PBS containing 0.7% Triton X-100 *v*/*v*) and then blocked with 10% normal goat serum in PBT (*v*/*v*) for 2 h at room temperature. After blocking, samples were incubated in 0.7% PBT containing 5% normal goat serum and primary antibodies for 48 h at 4 °C. After primary antibody incubation, brains were washed in 0.7% PBT six times for 30 min each at room temperature and then incubated in 0.7% PBT with 5% normal goat serum containing the secondary antibodies overnight at 4 °C. Brains were washed six times for 30 min each with PBT at room temperature and mounted in Vectashield (Vector Labs). The following antibodies and dilutions were used in whole-mount adult brain staining: rabbit anti-hypusine antibody (Merck, ABS1064; 1:1,000 dilution), guinea pig anti-eIF5A antibody (customized; 1:200 dilution), goat anti-guinea pig Alexa 555 (Invitrogen; 1:200 dilution), goat anti-rabbit Cy5 (Invitrogen; 1:200 dilution).

Image stacks of specimens were imaged on a Leica TCS SP8 confocal microscope (Leica Microsystems) using a ×40, 1.3 NA oil objective for whole-brain imaging with a voxel size of 0.3788 × 0.3788 × 0.9997 micron^3^. Images were quantified using ImageJ software. In brief, the average intensity z-projection was performed with ~25 stacks of each brain from the beginning of the antennal lobe to the end of the antennal lobe and the mean grey value of the central brain was measured.

### *C. elegans*

#### *C. elegans* strains

We followed standard procedures for *C.* *elegans* maintenance and other genetic manipulations^98^. The nematode-rearing temperature was kept at 20 °C unless noted otherwise. The following strains, available at the *Caenorhabditis* Genetics Center, were used: N2: WT Bristol isolate, HZ589: bpIs151 (sqst-1p::sqst-1::GFP + unc-76(+)), MAH215: sqIs11 (lgg-1p::mCherry::GFP::lgg-1 + rol-6).

#### *C. elegans* lifespan assays

Lifespan assays were performed at 20 °C. Synchronous animal populations were generated by hypochlorite treatment of gravid adults to obtain tightly synchronized embryos that were allowed to develop into adulthood under appropriate, defined conditions.

For RNAi lifespan experiments, worms were placed on Nematode Growth Medium plates containing 2 mM IPTG and seeded with HT115 (DE3) bacteria transformed with either the pL4440 vector or the test RNAi construct. The progeny was grown at 20 °C unless noted otherwise, through the L4-young adult larval stage and then transferred to fresh plates in groups of around 30 worms per plate for a total of at least 155 individuals per experiment. Animals were transferred to fresh plates every 2 days thereafter and examined every day for touch-provoked movement and pharyngeal pumping until death. Worms that died owing to internally hatched eggs, an extruded gonad or desiccation due to crawling on the edge of the plates were censored and incorporated as such into the dataset. Each survival assay was repeated at least four times.

For IF (IF^48:48^), synchronized young adult worms raised on Nematode Growth Medium plates with live HT115 were transferred to plates containing floxuridine (120 μM), seeded with HT115 RNAi-transformed bacteria, which were UV-killed before animal transfer. On day 2 of adulthood, worms were divided into ad libitum and IF. Worms in the ad libitum groups were fed ultraviolet-killed HT115 throughout their lifespan. Worms in IF were placed on plates with UV-killed HT115 or without food (seeded bacteria) alternatively every other day. All worms were transferred to freshly seeded plates every 2 days^99^.

#### *C. elegans* spermidine and rapamycin treatments

Lifespan assays with spermidine and rapamycin supplementation were performed as previously described^23,100^. In brief, SPD (Sigma, S0266) was used at a final concentration of 0.2 mM. Sterilized water solution of SPD was applied to the top of the RNAi bacterial lawn after being killed by UV irradiation for 15 min (0.5 J) using a UV cross-linker (bio-Link BLX-E365;Vilber Lourmat). Rapamycin (LC Laboratories) was dissolved in DMSO and added to the agar plate to a final concentration of 50 μM. Control plates contained an appropriate concentration of DMSO (<0.1%). SPD and rapamycin experiments were performed simultaneously, thus sharing the same control. Drug administration was performed on late-L4 worms unless otherwise noted. RNAi, SPD or rapamycin treatments were continued throughout life. In autophagy experiments, identical concentrations of SPD (0.2 mM) and rapamycin (50 μΜ) were administered and autophagic events were assessed at the first round of IF.

#### *C. elegans* molecular cloning and RNA interference

RNAi was performed by feeding methods^101^ and was performed lifelong, starting from egg hatching. To generate the RNAi constructs, gene-specific fragments of interest were amplified by PCR directly from the *C.* *elegans* genomic DNA using appropriate primer sets. The PCR-generated fragments were initially inserted into the TOPO-pCRII vector and then sub-cloned in the pL4440 plasmid vector. The final constructs were transformed into HT115 (DE3) *Escherichia* *coli* bacteria.

For *argn-1(RNAi)*, a 1,409-bp fragment was amplified by using the primer set:

For_argn-1: 5′-ATGAAAAAGTCTACACAACTCGCCAGA-3′ and

Rev_argn-1: 5′-TCACATTGCTCTTGTAATTTTCTGAGATTG-3′.

For *dhps-1(RNAi)*, a 2,420-bp fragment was amplified by using the primer set:

For_dhps-1: 5′-ATGAGCACCAACGAAGCAGCAG-3′ and

Rev_dhps-1: 5′-ATGCTTGGCCGCCCAATACA-3′.

For *odc-1(RNAi)*, a 1,327-bp fragment was amplified by using the primer set:

odc-1_For: 5′-ATGATTTCTCAATTCGAAATTATTGGTGAC-3′ and

odc-1_Rev: 5′-ATCACATACATCGGCACAGGCTTC-3′.

For *smd-1(RNAi)*, we amplified a 1,724-bp fragment by using the primer set:

For_smd-1: 5′-ATGTCTGCCACGTCTGCCAC-3′ and

Rev_smd-1: 5′-TCCTCGTCGCTCGATGATGA-3′.

For *spds-1(RNAi)*, a 1,714-bp fragment was amplified by using the primer set:

For_spds-1: 5′-ATGAACAAGCTGCACAAGGGATG-3′ and

rev_spds-1: 5′-CTTGCGATGACAAAATTCCATCCTC-3′.

#### *C. elegans* mRNA quantification

Quantification of mRNA levels was performed as previously described^102^. Total mRNA was isolated from synchronized adults, lysed in 250 μl TRIzol by freeze-cracking (Invitrogen). For complementary DNA synthesis, mRNA was reverse transcribed using an iScriptTM cDNA Synthesis kit (Bio-Rad). Quantitative PCR was performed in triplicate using a Bio-Rad CFX96 Real-Time PCR system (Bio-Rad). The housekeeping gene *act-3* was used as an internal normalization control. In each experiment, three technical replicates were used per sample.

For *act-3* mRNA detection, the following set of primers was used:

act-3 RT Fw: 5′-ATCCGTAAGGACTTGTACGCCAAC-3′ and

act-3 RT Rev: 5′-CGATGATCTTGATCTTCATGGTTC-3′.

For *smd-1* mRNA detection, the following set of primers was used:

smd-1RT_For: 5′-CTCAAACAGGATAGATGGTACCTTTACACAT-3′ and

smd-1RT_Rev: 5′-TTGTCAATACCGGCTCGCATG-3′.

For *spds-1* mRNA detection, the following set of primers was used:

spds-1RT_For: 5′-GATGTGCTGGTTTTTGAGAGCACA-3′ and

spds-1RT_Rev: 5′-ATCTCCTCCACCAATGATAAGTACACGT-3′.

For *dhps-1* mRNA detection, the following set of primers was used:

dhps-1RT_For: 5′-GGTGACCAGTGGGCTTCGTGAGGTAC-3′ and

dhps-1RT_Rev: 5′-TTATCATTCGGAATGAGCACATTGC-3′.

For *dohh-1* mRNA detection, the following set of primers was used:

dohh-1_RTFor: 5′-ATTGCTGATCCTTCAGTCAAAGATGTCCTC-3′ and

dohh-1_RTRev: 5′-GTTGAAGCTGATGGTGTTGGATCAA-3′.

For *odc-1* mRNA detection, the following set of primers was used:

odc-1RT_For: 5′-CATCTGCCTACAGGAATGCTCTTCA-3′

odc-1RT_Rev: 5′-TCACGAATTGTCTCGGCAATCTTC-3′.

For *argn-1* mRNA detection, the following set of primers was used:

argn-1RT_For: 5′-AGTGCAATGTCAGGAGTTACACAGACAT-3′ and

argn-1RT_Rev: 5′-CATCAACCCAAATAAGCCCAATATCTC-3′.

For *amx-3* mRNA detection, the following set of primers was used:

amx-3RT_For: 5′-TCCAGTCCGGTAGGTGAGCTT-3′ and

amx-3RT_Rev: 5′-CAGTTCAATCTAATGTTTCCCGCAGGTAT-3′.

For *hpo-15* mRNA detection, the following set of primers was used:

hpo-15RT_For: 5′-ACATGATGAGAAGACTGCAAGACGCTA-3′ and

hpo-15RT_Rev: 5′-TCCACTCTTTTAGAACGTGAACACTCAGAGT-3′.

For *d2023.4* mRNA detection, the following set of primers was used:

d2023.4RT_For: 5′-TGCATATTCAACATGGGTTGGGCAGTAT-3′ and

d2023.4RT_Rev: 5′-TCAACAGTGTCGTACAGAGCAATCGCAT-3′.

For *iff-1* mRNA detection, the following set of primers was used:

iff-1RT_For: 5′-ATCGAAGACGGGTTCTGCTCGCT-3′ and

iff-1RT_Rev: 5′-CAGGCAGCAACGACTTGAACCAAG-3′.

#### *C. elegans* heat stress assay

Five-day-old N2 (WT) animals (after the first round of fasting) were placed at 37 °C for 6 h. After recovery overnight at 20 °C, survival was assessed. Animals that did not exhibit either touch-provoked movement or pharyngeal pumping were considered dead. Five independent experiments were performed.

#### *C. elegans* detection of autophagy

To measure autophagic flux, the *C.* *elegans* strain MAH215 (sqIs11 (lgg-1p::mCherry::GFP::lgg-1 + rol-6)) was used (LGG-1 is the *C.* *elegans* orthologue of LC3/Atg8). Adult animals after the first round of fasting were measured. We calculated the number of autolysosomes (ALs) (mCherry-positive puncta). The number of mCherry-only puncta was calculated (the total number of mCherry-positive puncta − the number of GFP-positive + mCherry-positive puncta) as previously shown^103^. Z-stack images were acquired using the LEICA TCS SP8 laser scanning confocal microscope unit. Measurements were performed in the terminal bulb and at the area where the pharyngeal lumen was best observed. Additionally, we calculated the ratio of mCherry:GFP signal by measuring mean fluorescence intensity in the head region of animals, as previously reported^104^. We also calculated SQST-1::GFP (the *C.* *elegans* orthologue of SQSTM1) positive particles using the HZ589: bpIs151 (sqst-1p::sqst-1::GFP + unc-76(+)) strain. Particles were measured in the head region of the animals. Images were acquired using the EVOS Cell Imaging Systems.

#### *C. elegans* size measurement

Five-day-old N2 (WT) animals (after the first round of fasting) were anaesthetized and observed with ×4 magnification. Images were acquired using the EVOS Cell Imaging Systems. Animal periphery was precisely circled manually using the ‘polygon selections’ tool in ImageJ and the contained area was automatically calculated.

#### *C. elegans* immunoblotting

For sample preparation, synchronous animal populations were collected after 48 h of fasting and washed in M9 buffer. After washing, two volumes of homogenization buffer (20 mM Tris, pH 7.4, 20 mM NaCl and 1 mM MgCl_2_) containing complete mini proteinase inhibitor cocktail (Roche) in a final concentration 1× was added. One volume of beads (0.5 mm zirconium oxide beads) was added and samples were placed in a Bullet Blender Homogenizer (Model BT24M, Next Advance) for 3 min at speed 10. We used Bio-Rad protein assay (Bio-Rad, 5000006) to determine the protein concentration of the supernatant. The final sample buffer (final concentration of 62.5 mM Tris-HCl, pH 6.8, 2% SDS, 8.7% glycerol, 0.004% bromophenol blue and 120 mM DTT) was added to the supernatant. In brief, before electrophoresis, samples were boiled at 95 °C for 5 min and centrifuged at 10,000 rcf for 1 min. Protein extract corresponding to 5 µg protein was loaded onto 4–12% Bis-Tris gels and electrophoresis was performed at 90–120 V. Proteins were wet-transferred to methanol-activated 0.45-µm PVDF membranes (Roth, T830.1) at 220 mA for 60–90 min using transfer buffer (10 mM CAPS/NaOH, pH 11 and 10% methanol). After blotting, membranes were blocked with blocking solution (1% dry milk powder in TBS, pH 7.4) for 1 h and then incubated with the primary antibodies overnight at 4 °C. After three washing steps in TBST for 5 min, membranes were incubated with secondary, HRP-linked antibodies for 1 h at room temperature. After three washing steps in TBST for 5 min, signals were detected with a ChemiDoc detection system (Bio-Rad) and Clarity Western ECL Substrate (Bio-Rad) using the ‘optimal exposure’ setting. Band intensities were quantified using ImageLab v.5.2 (Bio-Rad) using the rectangular volume tool with local background adjustment. Primary antibodies were anti-hypusine (Merck, ABS1064-I, 1:1,000 dilution in blocking solution) and anti-GAPDH clone GA1R (Thermo Fisher Scientific, MA5-15738, 1:10,000 dilution in blocking solution). Secondary antibodies were HRP-linked anti-mouse IgG (Sigma, A9044, 1:10,000 dilution in blocking solution) or HRP-linked anti-rabbit IgG (Sigma, A0545, 1:10,000 dilution in blocking solution).

### Mouse

#### Fasting and caloric restriction protocols in mice

##### Acute fasting for polyamine measurement

*C57BL/6J:Rj* male and female mice were purchased from Janvier Labs at the age of 3–4 months. Animals were housed in groups of four mice in individually ventilated cages (Tecniplast Type 2L) under specific-pathogen-free (SPF) conditions in a 14:10-h light–dark cycle with access to food (standard maintenance chow, Ssniff, cat. no. V1536) and autoclaved tap water ad libitum. Autoclaved nest material and paper houses served as cage enrichment for mice. Animals were randomly assigned to fasting or control groups. Fasting was performed overnight for 14–16 h by food withdrawal, starting between 16:00 and 17:00 (lights off at 20:00), with water ad libitum at an age of 5–6 months.

##### Protocol 1 (IF^16:8^)

Male *C57BL/6J:Rj* mice were purchased (from Janvier Labs) at the age of 16–17 months. Animals were housed in groups of four mice in individually ventilated cages under SPF conditions in a 12-h light–dark cycle with access to standard chow ad libitum until begin of the fasting training followed by intermittent fasting. Specifically, mice were subjected to 3 weeks of fasting training, thereby reducing the daily feeding time window every two or three days from 24 to 14, 12, 10 and finally 8 h, to ensure that the aged mice slowly adapted to the final intermittent fasting (IF^16:8^) regimen, with access to food from 7:00 to 15:00 during the light phase, except at weekends, when they had access to food ad libitum. During the feeding time period, the animals had unrestricted access to food. DFMO (a kind gift from P. M. Woster, Medical University of South Carolina) aqueous solution was passed through a sterile filter and prepared fresh every week in the drinking water at 1% or 0.5% *w*/*v*. Before the experiments, the animals were randomly divided into four groups: ad libitum, ad libitum + DFMO, IF and IF + DFMO. The treatments were initiated at the age of 17–18 months. DFMO was administered at the concentration of 1% *w*/*v* for 4 weeks and then reduced to 0.5% *w*/*v* for the remainder of the experiment.

##### Protocol 2 (IF^CR^)

*C57BL/6J:Rj* male mice were purchased from Janvier Labs, France at the age of 18–19 months. Animals were housed in groups of four mice in individually ventilated cages (Tecniplast Type 2L) under SPF conditions in a 14:10-h light–dark cycle with access to food (standard maintenance chow, Ssniff, cat. no. V1536) and autoclaved tap water ad libitum. Autoclaved nest material and paper houses served as cage enrichment for mice. Animals were randomly assigned to fasting or control groups. Baseline calorie intake was calculated across the whole cohort over 4 weeks (4.3 g average food intake per mouse and day). Intermittent fasting was performed as described previously^50^ starting at an age of ~20 month and the mice were randomly assigned to the groups. In brief, IF^CR^ groups were fed once daily, shortly before the dark phase, with a single meal providing 70% of the cohort’s baseline calorie intake. The mice quickly consumed the allocated food portions and fasted for the remainder of the day, as manually verified in regular early morning visits. DFMO, prepared as described above, was given ad libitum at 0.25 and 0.5% (*w*/*v*) in the drinking water.

##### Protocol 3 (IF^24:24^)

Male and female mice 8–12 weeks of age were randomly assigned into four groups using randomizer.org. The control group (ad lib) was given free access to food and water during the whole experiment and received no further treatment. The feeding regimen of the IF^24:24^ group involved restricting access to food for 24 h, followed by 24 h food ad libitum. The IF + DFMO group was, in addition to fasting, treated with 1% (*w*/*v*) DFMO, prepared as described above, in the drinking water. The SPD group was given free access to food and was treated with 3 mM SPD (Sigma, 85558; from 1 M aqueous stock, pH 7.4) in the drinking water, prepared as previously described^23^. DFMO and SPD were exchanged two and three times every week, respectively.

##### Protocol 4 (caloric restriction)

C57BL/6J mice were obtained from The Jackson Laboratory. Mice were bred at the National Institute on Aging (NIA) and fed chow diets (NIH-31 diet; T.9717.15, Envigo) ad libitum or were on a 30% CR, starting at 9 months of age, as described previously^31^. CR mice were fed between 6:30 and 8:30.

#### Transgenic mice

Male mice overexpressing human IGF1R specifically in cardiomyocytes (IGF1R^tg^ mice)^40^ and male mice expressing a dominant negative phosphoinositide 3-kinase (PI3K) p110α mutant with impaired catalytic activity restricted to cardiomyocytes (dnPI3K mice)^41^ were used. In brief, a truncated p110 mutant with p85 binding domains, but lacking the kinase domain (p110Δkinase), was generated, and the p110Δkinase gene, together with FLAG epitope tag, was cloned into the αMyHC promoter construct to produce dnPI3K transgenic mice^41^. Both transgenic mouse models and their WT littermates were generated on the FVB/N genetic background and were killed at 3–9 months of age for heart tissue analysis, which included immunoblotting and polyamine measurements.

#### Healthspan measurements in aged mice

Various healthspan measurements were performed on IF^CR^ mice, with adapted protocols as detailed previously^105^. The measurements were performed in the morning until noon and the operators were blinded to the group allocation. In brief, grip strength (two paws and four paws, on different days) was assessed with a grip strength meter (TSE), according to the manufacturer’s manual. The maximum grip strength of three repeats was counted and normalized to body weight of the animal on the test day. A visual frailty index was performed as previously described^106^, which assesses several phenotypical alterations occurring with age in mice. Body composition of physically restrained animals was analysed with an NMR MinspecAnalyzer (Bruker). A four-limb grid-hanging test was performed at a height of 35 cm above a cushioned cage and the time to fall was recorded manually. Body surface temperature was recorded in the lower abdomen with a handheld infra-red thermometer and the maximum of three recordings was counted.

#### Echocardiography in aged mice

Cardiac function and dimensions were evaluated in lightly anaesthetized mice (4–5% for induction; 1–1.5% isoflurane for maintenance) by transthoracic echocardiography using a high-resolution micro-imaging system Vevo3100 (Fujifilm VisualSonics). Animal temperature was kept at 37 °C using a temperature-controlled heating platform, on which mice were placed in a supine position with their limbs in direct contact with electrode pads for heart rate assessment. Pre-warmed ultrasound transmission gel was spread on a shaved chest to obtain cardiac tracings in the parasternal long axis using 55 MHz linear-array probe. As previously described^42^, M-mode tracings were used to evaluate cardiac walls thickness and internal left ventricular (LV) dimensions at the level of the papillary muscles during systole and diastole. The ratio between peak early filling velocity of transmitral flow (E) and the corresponding mitral valve annulus velocity (e′) was assessed using pulsed wave and tissue Doppler imaging, respectively. All measures were averaged from at least three cardiac cycles under stable conditions.

#### K/BxN serum transfer arthritis model

BALB/cJRj mice were purchased from Janvier Labs. Mice were kept in a temperature- and humidity-controlled facility with free access to water and a 12-h light–dark cycle. All experiments were performed using age- and sex-matched littermate controls.

After 3 weeks of intermittent fasting/treatment, 6 h after re-feeding, 100 µl serum from arthritic transgenic K/BxN mice (obtained from the animal facility of the University of Erlangen) were administered to mice via intraperitoneal injection. The severity of K/BxN serum transfer arthritis^107^ was then clinically assessed by visual evaluation of swelling and redness in each paw, including the tarsus and carpus joints as previously described^108^. In short, the following scoring system was used: 0, no erythema or swelling; 1, erythema and swelling in up to two joints; 2, erythema and swelling in more than two joints or slight swelling of the ankle; 3, marked swelling of the ankle; and 4, substantial swelling of the whole paw, including toes and fingers. Scores of all paws were added together and AUCs of arthritis courses were calculated. IF and DFMO treatments were continued until the end of the experiment 24 days after serum transfer.

#### Detection of polyamines

Tissues were dissected and snap-frozen in liquid nitrogen after killing animals by a final blood draw (from retro-orbital plexus) under deep isoflurane narcosis followed by cervical dislocation. Serum was obtained via 30 min incubation at room temperature, followed by centrifugation at 1,000–2,000 rcf for 10 min at 4 °C. Appropriate amounts of frozen tissue (10–50 mg) were homogenized in varying volumes of 5% TCA to yield a final SPD concentration of 50–1,000 ng ml^−1^ (corresponds to the validated linear range of detection) in the final extract. Polyamine extraction and measurement were performed as previously described, with adapted analyte concentrations in the calibration solutions^82^ (see also ‘Yeast’ section).

#### Immunoblotting

For protein extraction, tissue was homogenized in lysis buffer (150 mM NaCl, 20 mM Tris-HCl, pH 7.5, 1 mM EGTA, 1 mM EDTA, 1% Triton X-100, 2× Complete protease inhibitor cocktail (Roche) and 1× PhosSTOP (Roche); 400 µl lysis buffer per 30 mg of tissue) for 30 s with a Turrax (IKA T10 basic) followed by an incubation for 30 min at 4 °C with continuous rotation. The homogenized samples were centrifuged for 10 min at 20,000 rcf at 4 °C. We used a Bio-Rad protein assay (Bio-Rad 5000006) to determine the protein concentration of the supernatant. Final sample buffer (final concentration: 62.5 mM Tris-HCl, pH 6.8, 2% SDS, 8.7% glycerol, 0.004% bromophenol blue and 120 mM DTT) was added to the supernatant. In brief before electrophoresis, samples were boiled at 95 °C for 5 min and centrifuged at 10,000 rcf for 1 min. Protein extract corresponding to 20 µg protein was loaded onto 4–12% Bis-Tris gels and electrophoresis was performed at 90–120 V. Proteins were transferred to methanol-activated 0.45-μm PVDF membranes for 90 min at 220 mA using transfer buffer (10 mM CAPS pH 11 and 10% methanol). The membranes were then incubated in blocking solution (3% dry milk powder in TBS, pH 7.4) for 1 h and subsequently incubated with the primary antibody overnight at 4 °C. Membranes were washed three times with TBST for 5 min and subsequently incubated with the secondary antibody for 1 h at room temperature. After three washing steps in TBST for 5 min, signals were detected with a ChemiDoc detection system (Bio-Rad) and Clarity Western ECL Substrate (Bio-Rad) using the ‘optimal exposure’ setting. Band intensities were quantified using ImageLab v.5.2 (Bio-Rad) using the rectangular volume tool with local background adjustment. For re-probing membranes, Restore PLUS Western Blot Stripping Buffer (Thermo Fisher Scientific, 46430) was used according to the manufacturer’s protocol. Primary antibodies were anti-hypusine (Merck, ABS1064-I, 1:1,000 dilution), anti-eIF5a (BD Biosciences, 611977, 1:10,000 dilution) and anti-GAPDH clone GA1R (Thermo Fisher Scientific, MA5-15738, 1:10,000 dilution) in 1% dry milk powder in TBS, pH 7.4. Secondary antibodies were HRP-linked anti-mouse IgG (Sigma, A9044, 1:10,000 dilution in 1% dry milk powder in TBS, pH 7.4) and HRP-linked anti-rabbit IgG (Sigma, A0545, 1:10,000 dilution in 1% dry milk powder in TBS, pH 7.4).

#### Ethical regulations

All animal experiments were performed in accordance with national and European ethical regulation (Directive 2010/63/EU) and approved by the responsible government agencies (Bundesministerium für Wissenschaft, Forschung und Wirtschaft, BMWFW; BMWFW-66.007/0029-WF/V/3b/2017, GZ 2021-0.524.242, GZ 2022-0.137.213, BMWFW-66.010/0160-WF/V/3b/2014, BMWFW-66.010/0198-WF/V/3b/2017 and BMBWF-66.010/0042-V/3b/2018). Animal studies using protocol 3 (IF^24:24^) and the arthritis model were approved by the local ethical committee (District Government of Lower Franconia, 55.2-2532-2-1041-15). All experiments were conducted according to the guidelines of the Federation of European Laboratory Animal Science Associations. CR mice (protocol 4) were used in accordance with protocols approved by the Institutional Animal Care and Use Committee of the NIA.

### Human cells

#### Cell lines, media and compounds

All culture media and supplements for human cell culture were purchased from Gibco-Life Technologies. Plasticware was purchased from Greiner Bio-One. Cells used in this study were cultured in DMEM containing 10% FBS and non-essential amino acids (Gibco, 111400) at 37 °C under 5% CO_2_. Hanks’ balanced salt solution (HBSS; Gibco,14025092) was used for starvation experiments. DFMO, spermidine, rapamycin, torin-1, aminoguanidine, bafilomycin and chloroquine were obtained from Sigma-Aldrich. The pharmacological DHS inhibitor N1-guanyl-1,7-diamine-heptane (GC7) was purchased from Merck (259545). DFMO and GC7 were prepared as sterile filtered 50 mM stocks in ddH_2_O and stored in aliquots at −20 °C.

We used human glioblastoma H4 and human osteosarcoma U2OS cells (from ATCC), either in WT form or stably expressing GFP–LC3. Generally, cells were seeded in six-well plates at approximately 1 × 10^5^ cells per well. DFMO treatment was initiated after overnight attachment by exchanging the medium with freshly prepared DFMO-containing medium.

#### Cell line polyamine measurements

The plates were placed on ice and quickly washed with cold PBS (4 °C). Then, 500 µl of cold methanol:water (9:1 *v*/*v*, with internal standards) with 2% of sulfosalicylic acid were added to each well and the plate was stored overnight at −80 °C. Two wells at ~5 × 10^5^ cells per well were scrapped and pooled for one measurement in microcentrifuge tubes. For normalization purposes, we used reference wells for cell counting.

The suspension was vortexed for 30 s and centrifuged at 15,000 rpm (21,130 rcf) for 10 min at 4 °C. After the transfer of the whole supernatant to another set of microtubes, samples were evaporated. The dried samples were spiked with 200 µl of MilliQ water before injection in UHPLC–MS. Polyamine analysis was performed by LC–MS/MS with a 1290 UHPLC (Agilent Technologies) coupled to a QQQ 6470 (Agilent Technologies)^84^.

#### Cell line autophagy induction and microscopy

DFMO treatment (48–72 h) was continuously performed, whereas GC7 was used acutely during starvation. Trypsinized GFP–LC3-expressing cells were seeded in 384-well black microplates (~4,000 cells per well). Starvation with HBSS or treatment with rapamycin (10 µM; 2 mM DMSO stock stored in aliquots at −20 °C) or Torin-1 (300 nM; 60 µM DMSO stock stored in aliquots at −20 °C) was initiated after overnight attachment. Before starvation experiments, the cells were gently washed with warm PBS (37 °C). DMSO at a final concentration of 0.5% was used as the control treatment for rapamycin and torin-1. Fresh DMEM containing FBS and non-essential amino acids was used as the control treatment for starvation experiments. Chloroquine (50 µM in sterile filtered ddH_2_O) was used as an autophagic flux inhibitor and added to the wells after half of the treatment time, except for Extended Data Fig. 5r,s, where bafilomycin A1 (BafA1, 100 nM) was used.

Experiments involving spermidine supplementation (Sigma, 85558; from aqueous stocks, pH 7.4) were co-treated with aminoguanidine (from aqueous stocks, 1 mM, Sigma, 396494).

After treatment, cells were fixed with 3.7% paraformaldehyde (*w*/*v* in PBS containing 2 µg ml^−1^ Hoechst) for 20 min at room temperature. Microscopy images were acquired with an ImageXpress Micro Confocal microscope (Molecular Devices) equipped with a ×20 PlanApo objective (Nikon). Four view fields and six z-stacks were imaged per well. We used the Custom Module Editor functionality of the MetaXpress software (Molecular Devices) to analyse the images. First, using the nuclear Hoechst staining and cytoplasmic GFP signal, cells were segmented into nuclear and cytoplasmic regions. Then, cytoplasmic GFP–LC3 puncta were detected using automated thresholding, and their number and the cellular surface covered by GFP–LC3 puncta were analysed.

#### Cell line siRNA interference

U2OS cells (with or without stably expressing GFP–LC3) were seeded in 384-well imaging microplates at an approximate density of 1,000 cells per well (for autophagy measurements) or in six-well plates at an approximate density of 1 × 10^5^ cells per well cells per well (for polyamine measurements). Following 1 day of attachment, cells were transfected with siRNAs against *ODC1* or a cherry-picked siRNA library (Dharmacon) targeting selected genes associated with polyamine metabolism at a final concentration of 30 nM using the DharmaFECT transfection reagent (Dharmacon) according to the manufacturer’s instructions. Each gene was represented as four individual siRNAs and non-targeting siRNAs served as control (Supplementary Table 4). After 24 h, the medium was renewed and cells were left to adapt for an additional 24 h. Subsequently, cells were carefully washed with warm PBS and subjected to starvation with HBSS for 6 h.

Image acquisition was carried out as previously described. The obtained images were segmented and analysed with the freely available software R (https://www.r-project.org), integrated with the following packages: EBImage from the Bioconductor repository (https://www.bioconductor.org), MetaxpR (https://github.com/kroemerlab/MetaxpR), RBioFormats (https://github.com/aoles/RBioFormats), as well as MorphR (https://github.com/kroemerlab/MorphR). The primary region of interest was designated by a mask around the nucleus to enable cell enumeration, while a secondary cytoplasmic region of interest was employed for the segmentation of GFP–LC3 puncta. Following the exclusion of cellular debris and dead cells, the number of GFP–LC3 dots per cell was quantified and represented as fold change relative to the control.

#### Cell line RNA extraction and gene expression analysis

For RNA extraction, the RNeasy Plus Mini kit (cat. no. 74134, QIAGEN) was utilized. Cell pellets were stored at −80 °C before purifying RNA. Buffer RLT Plus was used to lyse the cell pellets. The lysate was centrifuged and subjected to further purification following the manufacturer’s instructions. All steps of the procedure were carried out at room temperature and all centrifugation steps were performed at 20–25 °C in a standard microcentrifuge. Approximately 1 μg total RNA was reverse transcribed using a Maxima First Strand cDNA Synthesis kit (cat. no. K1642, Thermo Fisher Scientific). Quantitative reverse transcription PCR (qRT–PCR) was conducted using PowerUp SYBR Green Master Mix (cat. no. A25776, Thermo Fisher Scientific) with a StepOnePlus Real-Time PCR System (Applied Biosystems, Thermo Fisher Scientific). The 2^−ΔΔCT^ method was applied for the analysis of real-time PCR data with the appropriate primers. All primers used for gene expression analysis are listed in Supplementary Table 5.

#### Cell lines immunoblotting

For immunoblotting, 2–3 wells from six-well plates were washed with PBS, then collected on ice with RIPA cell lysis buffer. Cells were scraped off and incubated for 30 min at 4 °C before centrifugation for 20 min at 12,000 rpm (13,523 rcf). The protein concentration in the supernatant was determined with a Bradford assay and the samples were stored at −20 °C until further processing. In brief before electrophoresis, samples were mixed with final sample buffer (final concentration of 62.5 mM Tris-HCl, pH 6.8, 2% SDS, 8.7% glycerol, 0.004% bromophenol blue and 120 mM DTT) and boiled at 95 °C for 5 min. Then, 10 µg total protein was loaded onto 4–12% Bis-Tris gels and run at 90–120 V. Proteins were transferred to 0.45-μm PVDF membranes for 90 min at 220 mA using transfer buffer (10 mM CAPS pH 11 and 10% methanol). The membranes were then incubated in blocking solution (3% dry milk powder in TBS, pH 7.4) for 1 h and subsequently incubated with the primary antibody overnight at 4 °C. Membranes were washed three times with TBST for 5 min and subsequently incubated with the secondary antibody for 1 h at room temperature. After three washing steps in TBST for 5 min, signals were detected with a ChemiDoc detection system (Bio-Rad) and Clarity Western ECL Substrate (Bio-Rad) using the ‘optimal exposure’ setting. For re-probing membranes, Restore PLUS Western Blot Stripping Buffer (Thermo Fisher Scientific, 46430) was used according to the manufacturer’s protocol. Band intensities were quantified using ImageLab v.5.2 (Bio-Rad) using the rectangular volume tool with local background adjustment. Primary antibodies were anti-hypusine (Merck, ABS1064-I, 1:1,000 dilution), anti-GAPDH clone GA1R (Thermo Fisher Scientific, MA5-15738, 1:10,000 dilution) and anti-eIF5A (611977, BD Biosciences, 1:10,000 dilution), diluted in 1% dry milk powder in TBST. Secondary antibodies were HRP-linked anti-mouse IgG (Sigma, A9044, 1:10,000 dilution in blocking solution) or HRP-linked anti-rabbit IgG (Sigma, A0545, 1:10,000 dilution in blocking solution).

### Human fasting trials

Written informed consent was obtained from all volunteers before they were enrolled in the studies, which were conducted in accordance with the principles of the Declaration of Helsinki.

#### Cohort 1

The study protocol was approved by the medical council of Baden-Württemberg under the application number F-2018-118 and registered in the German Clinical Trials Register (ID DRKS00016657). The 109 participants were recruited among the in-house patients of the Buchinger Wilhelmi clinic Überlingen as previously described^109–111^. All participants underwent a 10 ± 3-day fasting period under medical supervision. A daily caloric intake of around 250 kcal was provided by 250 ml organic juice, 250 ml vegetable soup and 20 g honey. Serum samples were collected before the start of the fast and at the end of the 10 ± 3-day fasting period. Tubes were stored at −70 °C until further analysis.

#### Cohort 2

The 1-year observational study was performed in the Buchinger Wilhelmi clinic Überlingen and described in detail previously^112–115^. The medical council of Baden-Württemberg and the Ethics Committee of the Charité-University Medical Center Berlin approved the study protocol under application no. EA4/054/15 that was registered in the German Clinical Trials Register (ID DRKS00010111). Blood samples before and at the end of fasting were available for a subgroup of 63 participants who fasted more than 4 days according to the Buchinger Wilhelmi fasting programme. Serum samples were stored at −70 °C.

#### Cohort 3

Cohort 3 was previously described^116,117^. For the purpose of this study, we took a subset of study participants with available plasma samples (23 of an initial 30 performing alternate day fasting) after 12 and 36 h of fasting, stored them at −70 °C and performed targeted MS of spermidine as described below, which was previously not quantified.

#### Cohort 4

Clinical data were collected during an exploratory clinical trial. Healthy participants were included by written informed consent when presenting no manifest chronic diseases nor pregnancy or lactation. Recruitment and intervention were performed on an outpatient basis at the Immanuel Hospital Berlin, where the outpatient department of the Institute of Social Medicine, Epidemiology and Health Economics of the Charité – Universitätsmedizin Berlin is seated. The study protocol was approved by the institutional review board of Charité Universitätsmedizin Berlin (ID EA1/263/20) and is registered with ClinicalTrials.gov under NCT04739852.

Recruitment for healthy volunteers took place in August 2020. All measurements and the intervention were conducted in September 2020. Participants underwent a 5-day prolonged fasting period with a dietary energy intake of 200–350 kcal per day with vegetable broths and juices. These were followed by three days of gradual re-introduction of solid food intake, the meals containing mainly complex carbohydrates. PBMCs and blood were collected at baseline, after 1, 3 and 5 days of the fasting intervention, as well as on days 3 and 7 after re-introduction of food.

The blood of healthy volunteers was collected in EDTA-sampling tubes (Sarstedt, 02.1066.001) via venipuncture, carefully loaded on Biocoll solution (BioChrom, L6113) in Lecosep tubes (Z642843, Greiner) and centrifuged at 684 rcf for 30 min (brakeless running down). PBMCs were enriched by selecting the interphase of the Biocoll gradient. PBMCs of the interphase were washed twice with ice-cold PBS. Pellets were stored at −80 °C until they were further processed. This is the first publication of data from this cohort and the primary end points will be published elsewhere.

#### Detection of polyamines and other metabolites in clinical samples

##### Cohort 1

Polyamine quantification from serum was performed as previously described^82^.

##### Cohorts 2 and 3

Serum and plasma samples were treated following a previously described protocol^84^. In brief, 50-µl samples were vortexed for 5 min with 500 µl ice-cold extraction mixture (methanol:water, 9:1, −20 °C, with a cocktail of internal standards) and then centrifuged (10 min at 15,000 rpm (21,130 rcf), 4 °C). Several fractions were then split to be analysed by LC–MS and GC–MS^84^. Polyamine analyses were performed by LC–MS/MS with a 1290 UHPLC (Agilent Technologies) coupled to a QQQ 6470 (Agilent Technologies) as previously described^85^.

All targeted treated data were merged and cleaned with a dedicated R (v.4.0) package (@Github/Kroemerlab/GRMeta).

##### Cohort 4 serum

For the extraction of polar metabolites from human serum samples, a mixture of methyl tert-butyl ether (Sigma-Aldrich, 650560), methanol (Biosolve, 136841) and water (Biosolve, 232141) (all LC–MS grade) was utilized in a 50:30:20 (*v*:*v*:*v*) ratio as the sample extraction buffer. The sample extraction buffer included the following internal standards: U-^13^C^15^N-labelled amino acids at a final concentration of 0.25 µM (2.5 mM in 0.1 N HCl, Cambridge Isotope Laboratories, MSK-A2-1.2), citric acid d_4_ at 0.02 µg ml^−1^ (100 µg ml^−1^ in H_2_O, Sigma-Aldrich, 485438-1 G), ATP ^13^C_10_ at 0.1 µg ml^−1^ (1 mg ml^−1^ in 5 mM Tris-HCl, Sigma-Aldrich, 710695), AMP ^13^C_10_, ^15^N_5_ at 0.1 µg ml^−1^ (1 mg ml^−1^ in 5 mM Tris-HCl, Sigma-Aldrich, 650676), ADP ^15^N_5_ at 0.1 µg ml^−1^ (1 mg ml^−1^ in 5 mM Tris-HCl, Sigma-Aldrich, 741167) and EquiSPLASH LIPIDOMIX at 0.02 µg ml^−1^ (100 µg ml^−1^, Avanti Polar Lipids, 30731). The sample extraction buffer was freshly prepared and cooled to −20 °C before a volume of 1 ml of the chilled sample extraction buffer was added to 50 µl human serum. This mixture was incubated at 4 °C for 30 min with shaking at 1,500 rpm in a thermomixer (VWR, Thermal Shake lite). Post-incubation, the samples were centrifuged (Eppendorf, Centrifuge 5425R) at 4 °C for 10 min at 21,000 rcf and the cleared supernatant was transferred to a 2-ml tube. To this supernatant, 200 µl methyl tert-butyl ether and 150 µl water were added, followed by incubation at 15 °C for 10 min with shaking at 1,500 rpm in a thermomixer. A subsequent centrifugation at 15 °C for 10 min at 16,000 rcf facilitated phase separation. Approximately 650 µl of the upper lipid-containing phase was transferred to a 1.5-ml tube (not included in this study). The remaining polar metabolite extract, approximately 600 µl, was left in the 2-ml tube after removal of the residual lipid phase. The polar metabolite extract was then distributed in two 1.5-ml tubes and dried in a SpeedVac concentrator (LaboGene, Scan Speed 40) at 20 °C at 1,000 rpm. The resulting dried metabolite pellets were stored at −80 °C until further analysis.

##### Cohort 4 PBMCs

For the extraction of polar metabolites from human PBMCs, a mixture of acetonitrile (Biosolve, 012041), methanol and water (all LC–MS-grade) was utilized in a 40:40:20 (*v*:*v*:*v*) ratio as the sample extraction buffer. The sample extraction buffer included the following internal standards: U-^13^C^15^N-labelled amino acids at a final concentration of 0.25 µM (2.5 mM in 0.1 N HCl, Cambridge Isotope Laboratories, MSK-A2-1.2), citric acid d_4_ at 0.02 µg ml^−1^ (100 µg ml^−1^ in H_2_O, Sigma-Aldrich, 485438-1 G), ATP ^13^C_10_ at 0.1 µg ml^−1^ (1 mg ml^−1^ in 5 mM Tris-HCl, Sigma-Aldrich, 710695), AMP ^13^C_10_, ^15^N_5_ at 0.1 µg ml^−1^ (1 mg ml^−1^ in 5 mM Tris-HCl, Sigma-Aldrich, 650676), and ADP ^15^N_5_ at 0.1 µg ml^−1^ (1 mg ml^−1^ in 5 mM Tris-HCl, Sigma-Aldrich, 741167). The sample extraction buffer was freshly prepared and cooled to −20 °C before a volume of 1 ml of the chilled sample extraction buffer was added to the individual PBMC pellets. To dissolve the pellets, samples were subjected to 3–5 pulses of sonication using a tip sonicator (Bandelin, Sonopuls). This mixture was then incubated at 4 °C for 30 min with shaking at 1,500 rpm in a thermomixer. Post-incubation, the samples were centrifuged at 4 °C for 10 min at 21,000 rcf and the cleared supernatant was transferred to a 1.5-ml tube and the pellet (protein pellet) was set aside. The polar metabolite extract was then dried in a SpeedVac concentrator at 20 °C at 1,000 rpm. The resulting dried metabolite pellets were stored at −80 °C until further analysis. The residual liquid was removed from the protein pellet and the pellet was left to air dry at room temperature for protein quantification using the Pierce 660 nm BCA protein quantification kit (Thermo Fisher Scientific, 22660) according to the manufacturer’s instructions. For protein extraction, pellets were re-suspended in a protein extraction buffer (50 mM Tris-HCl, pH 8.5, 1% SDS and 140 mM NaCl), homogenized with metal balls (3.5 mm) using a mixer mill (Retsch, MM 400) and diluted in an appropriate volume of protein extraction buffer before assay measurement.

##### Cohort 4: semi-targeted LC–high-resolution MS-based analysis of amine-containing metabolites

The LC–high-resolution MS analysis of amine-containing compounds was performed using a QE-Plus high-resolution mass spectrometer coupled to a Vanquish UHPLC chromatography system (Thermo Fisher Scientific). In brief, dried sample extracts were dissolved in 150 µl LC–MS-grade water for 10 min at 4 °C in a shaker at 1,500 rpm. After centrifugation, 50 µl of the extracts were mixed with 25 µl of 100 mM sodium carbonate (Sigma-Aldrich), followed by the addition of 25 µl 2% (*v*/*v*) benzoylchloride (Sigma-Aldrich) in acetonitrile (ULC/MS grade, Biosolve). The derivatized samples were thoroughly mixed and kept at a temperature of 20 °C until analysis.

For the LC–high-resolution MS analysis, 1 µl of the derivatized sample was injected onto a 100 × 2.1-mm HSS T3 UPLC column (Waters). The flow rate was set to 400 µl min^−1^ using a binary buffer system consisting of buffer A (10 mM ammonium formate (Sigma-Aldrich) and 0.15% (*v*/*v*) formic acid (Sigma-Aldrich) in ULC/MS-grade water (Biosolve)). Buffer B consisted of acetonitrile (ULC/MS-grade, Biosolve). The column temperature was set to 40 °C, while the LC gradient was: 0% B at 0 min, 0–15% B 0–4.1 min; 15–17% B 4.1–4.5 min; 17–55% B 4.5–11 min; 55–70% B 11–11.5 min, 70–100% B 11.5–13 min; B 100% 13–14 min; 100–0% B 14–14.1 min; 0% B 14.1–19 min; 0% B. The mass spectrometer (Q-Exactive Plus) was operating in positive ionization mode recording the mass range m/z 100–1,000. The heated electrospray ionisation (ESI) source settings of the mass spectrometer were spray voltage 3.5 kV, capillary temperature 300 °C, sheath gas flow 60 a.u., aux gas flow 20 a.u. at 330 °C and the sweep gas was set to 2 a.u. The RF lens was set to a value of 60.

LC–MS data analysis was performed using the open-source software El Maven^118^ (v.0.12.0). For this purpose, Thermo raw mass spectra files were converted to mzML format using MSConvert^119^ (v.3.0.22060, Proteowizard). The identity of each compound was validated by authentic reference compounds, which were measured at the beginning and the end of the sequence. For data analysis, the area of the protonated (M + nBz + H)^+^ (where, nBz indicates the number of benzoyl moieties attached to each compound) mass peaks of every required compound were extracted and integrated using a mass accuracy of <5 ppm and a retention time tolerance of <0.05 min compared with the independently measured reference compounds. Peak areas were then normalized to internal standards and to the protein content (PBMCs) or to the median of the total ion count (serum). For cohort 4, data were further normalized across the average values of the time points per participant.

#### Protein extraction and capillary immunoblotting (cohort 4)

Protein extracts from PBMCs were obtained by lysing cells (approx. 1–2 × 10^6^ cells) in 80 µl T-PER extraction buffer (Thermo Scientific, 78510), supplemented with protease (Sigma, P2714) and phosphatase (Roche, 04906837001) inhibitor cocktail. Protein extracts (1–2 µg) were separated by capillary electrophoresis on Jess (ProteinSimple) using 12–230 kDa cartridges (ProteinSimple, SM-W004) and the following primary antibodies for detection: anti-hypusine (1:50 dilution, ABS1064-I, Merck), anti-eIF5a (1:50 dilution, 611977, BD Biosciences) and anti-ODC1 (1:50 dilution, PA5-21362, Thermo Scientific). The signals were normalized to total protein using the protein normalization module (DM-PN02, ProteinSimple).

### Statistics and reproducibility

All data were checked for normality using QQ plots and histograms. In the case of nonparametric data distribution and feasibility, data were log_2_-transformed and tested again for normality. In the case of persistent nonparametric data distribution, appropriate statistical nonparametric tests were used. A post hoc Holm-Šídák’s test (false discovery rate 5%) or Dunn’s test were used to account for multiple testing within a dataset. For statistical analysis of the survival curves, we used log-rank tests with post hoc Bonferroni corrections for multiple testing. Outlier analysis using the ROUT method (Q = 0.1–0.5%) and outlier exclusion were performed on the metabolite measurements of human biological samples. We used GraphPad Prism 10.1.0 (www.graphpad.com) for general data visualization and statistics. All applied statistical tests are listed in the figure legends. For specific details of the OMICS data analyses see the respective Methods sub-sections. Sample size corresponds to either individual measurements of animals or human participants, or groups of cells (yeast and human cell lines) or flies. All data points shown are independent samples and all samples on immunoblots were biologically independent. Immunoblots used for quantification are in the source data. All experimental replications yielded comparable outcomes.

### Reporting summary

Further information on research design is available in the Nature Portfolio Reporting Summary linked to this article.

## Online content

Any methods, additional references, Nature Portfolio reporting summaries, source data, extended data, supplementary information, acknowledgements, peer review information; details of author contributions and competing interests; and statements of data and code availability are available at 10.1038/s41556-024-01468-x.

## Supplementary information

Supplementary Information Supplementary Figs. 1–6 and uncropped immunoblots for Supplementary Figs. 5 and 6.

Reporting Summary

Supplementary Table 1 Supplementary Tables 1–5.

Supplementary Data 2 Numerical Source Data Supplementary Table 3.

Supplementary Data 1 Numerical Source Data for Supplementary Fig. 1.

Supplementary Data 2 Numerical Source for Supplementary Data Fig. 2.

Supplementary Data 3 Numerical Source Data for Supplementary Fig. 3.

Supplementary Data 4 Numerical Source Data for Supplementary Fig. 4.

Supplementary Data 5 Numerical Source Data for Supplementary Fig. 5.

Supplementary Data 6 Numerical Source Data for Supplementary Fig. 6.

## Source data

Source Data Fig. 1 Numerical Source Data.

Source Data Fig. 2 Numerical Source Data.

Source Data Fig. 3 Numerical Source Data.

Source Data Fig. 3 Uncropped western blots.

Source Data Fig. 4 Numerical Source Data.

Source Data Fig. 5 Numerical Source Data.

Source Data Fig. 6 Numerical Source Data.

Source Data Fig. 6 Uncropped western blots.

Source Data Extended Data Fig. 1 Numerical Source Data.

Source Data Extended Data Fig. 2 Numerical Source Data.

Source Data Extended Data Fig. 3 Numerical Source Data.

Source Data Extended Data Fig. 4 Numerical Source Data.

Source Data Extended Data Fig. 4 Uncropped western blots.

Source Data Extended Data Fig. 5 Numerical Source Data.

Source Data Extended Data Fig. 6 Numerical Source Data.

Source Data Extended Data Fig. 7 Numerical Source Data

Source Data Extended Data Fig. 8 Numerical Source Data.

Source Data Extended Data Fig. 9 Numerical Source Data.

Source Data Extended Data Fig. 9 Uncropped western blots.

Source Data Extended Data Fig. 10 Numerical Source Data.

## Data Availability

The MS yeast proteomics raw data together with the processing log files have been deposited to the ProteomeXchange Consortium via the PRIDE (http://www.ebi.ac.uk/pride) partner repository with the dataset identifier PXD035909. The metabolomics and all other data are included in Source Data. All other data supporting the findings of this study are available from the corresponding author on reasonable request. Source data are provided with this paper.
