# Supplementary material for: Spermidine is essential for fasting-mediated autophagy and longevity
Source: Nat Cell Biol. 2024 Aug 8;26(9):1571–84. doi: 10.1038/s41556-024-01468-x (PMC11392816; doi:10.1038/s41556-024-01468-x)
Supplement: Supplementary file 1 — Supplementary Figs. 1–6 and uncropped immunoblots for Supplementary Figs. 5 and 6. [file 41556_2024_1468_MOESM1_ESM.pdf]

nature cell biology

Supplementary information

https://doi.org/10.1038/s41556-024-01468-x

Spermidine is essential for fasting-mediated autophagy and longevity

In the format provided by the authors and unedited

Supplementary Material

Table of Contents

- Figures S1-S6
- Uncropped Immunoblots for S5 and S6

Supplementary Figures

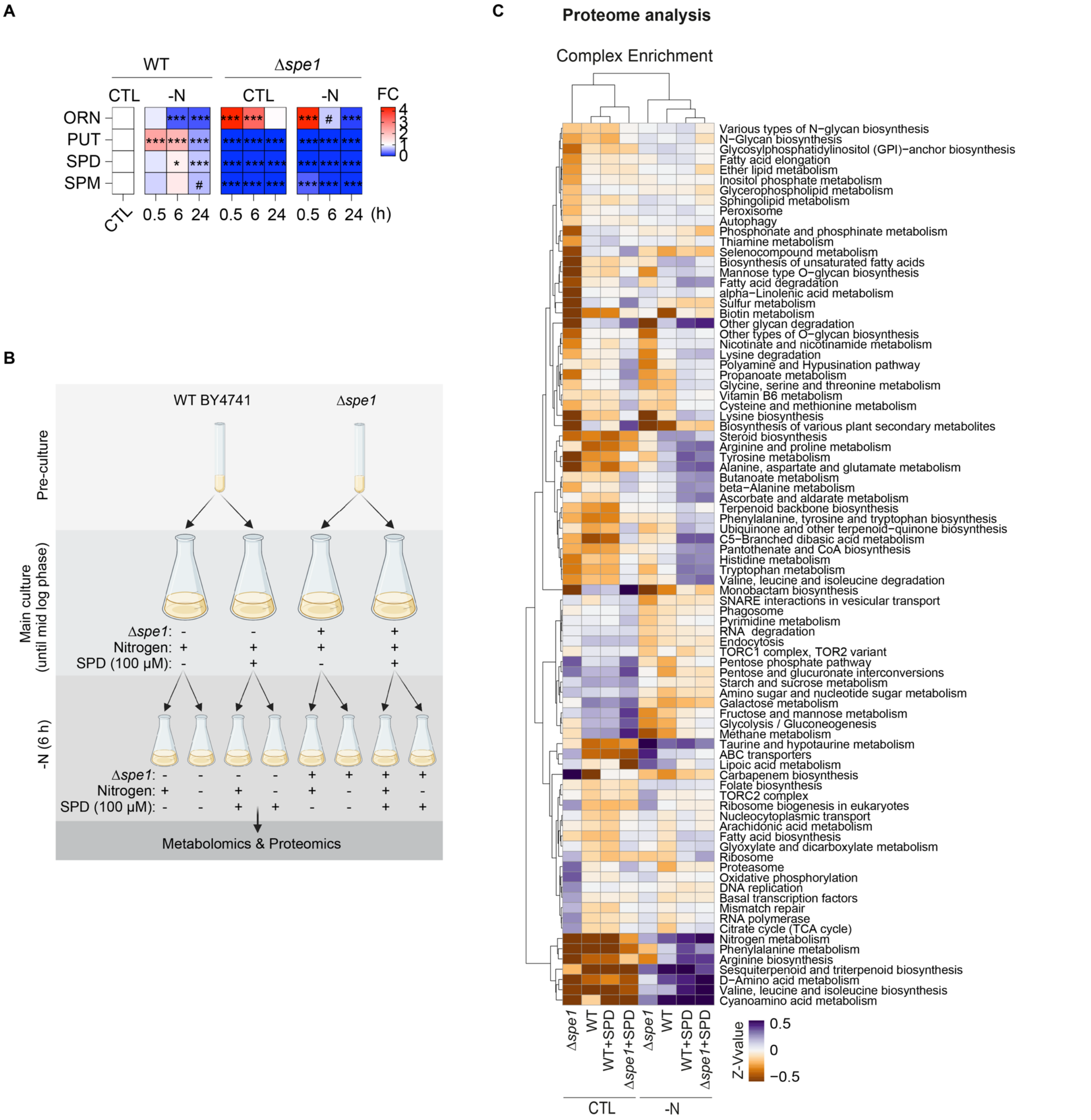

Figure S1: Spermidine is required for correct proteomes during nitrogen starvation in yeast. (A) Polyamine levels of BY4741 WT and Δ*spe1* yeast shifted to -N for the indicated times. Data normalized to the mean of the control (CTL) condition at every time point. N=6 biologically independent samples (yeast cultures). Two-way ANOVA with Holm-Šídák's multiple comparisons test. Heatmap shows means. * P<0.05, ** P<0.01, *** P<0.001, # P<0.2. **(B)** Experimental layout for the analysis of yeast metabolomes and proteomes. Figure created with Biorender.com (**C**) Protein complex enrichment analyses of yeast proteomes using all annotated yeast KEGG pathways as well as complex portal annotations of the TORC complex (CPX-1715, CPX-1716, CPX-1717) visualized with their referring Z-Values, resulting from absolute differential protein expression of the referring complexes. Complexes with a Z-Value >0.2/<-0.1 displayed in a heatmap including a hierarchical clustering employing the Euclidean distance metric. N=6 biologically independent samples (yeast cultures). Source numerical data are available in source data.

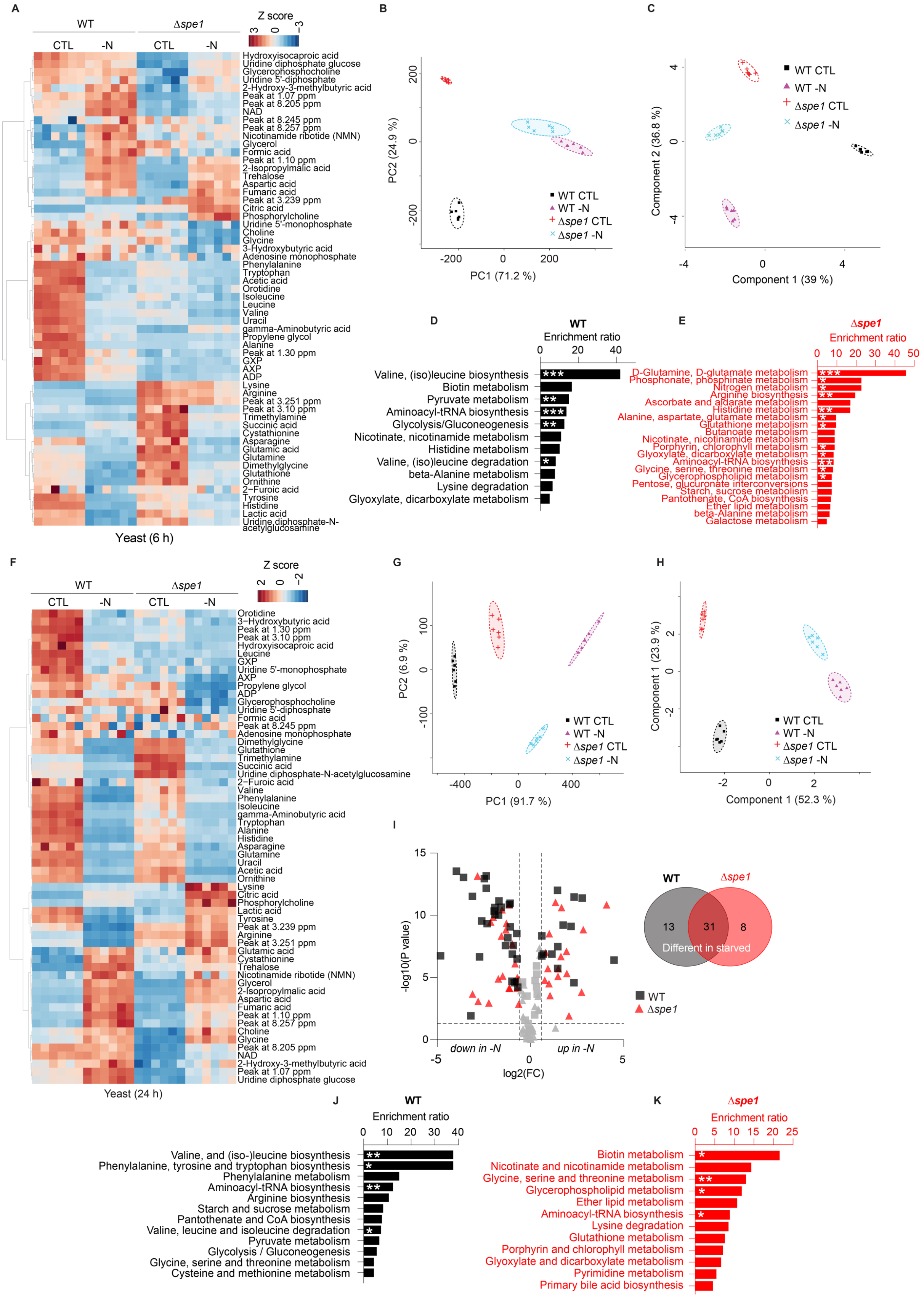

Figure S2: Metabolomic analysis by NMR spectroscopy reveals prominent differences between WT and Δ*spe1* yeast strains after 6 and 24 hours of nitrogen deprivation. (A-B) Heatmap and principal component (PC) analysis of yeast WT and

Δ*spe1* metabolomes after 6 hours -N. Unassigned NMR signals are labeled according to their NMR chemical shift. N=4 biologically independent samples (yeast cultures). **(C)** Sparse Partial Least Squares Discriminant Analysis (sPLS-DA) of yeast WT and Δ*spe1* metabolomes after 6 hours -N. N=4 biologically independent samples (yeast cultures). **(D-E)** Metabolite set enrichment analysis based on KEGG pathways of exclusive metabolites from [Fig. 2D] (raw *P*-values <0.2). **(F-G)** Heatmap and principal component (PC) analysis of *S. cerevisiae* WT and Δ*spe1* metabolomes after 24 hours -N. Unassigned NMR signals are labeled according to their NMR chemical shift. N=4 biologically independent samples (yeast cultures). **(H)** Sparse Partial Least Squares Discriminant Analysis (sPLS-DA) of *S. cerevisiae* WT and Δ*spe1* metabolomes after 24 hours -N. N=4 biologically independent samples (yeast cultures). **(I)** Volcano plot showing significantly different metabolites in WT or Δ*spe1* after 24 hours -N compared to the control medium. Venn diagram showing exclusive and overlapping significantly regulated metabolites. Two-tailed Student's *t*-tests with FDR-corrected *P*-values <0.05, FC (fold change) >1.5. N=4 biologically independent samples (yeast cultures). **(J-K)** Metabolite set enrichment analysis based on KEGG pathways of exclusive metabolites from [D] (raw *P*-values <0.2). <u>Statistics:</u> Asterisks indicate raw *P*-values. *<0.05, **<0.01, ***<0.001. Source numerical data are available in source data.

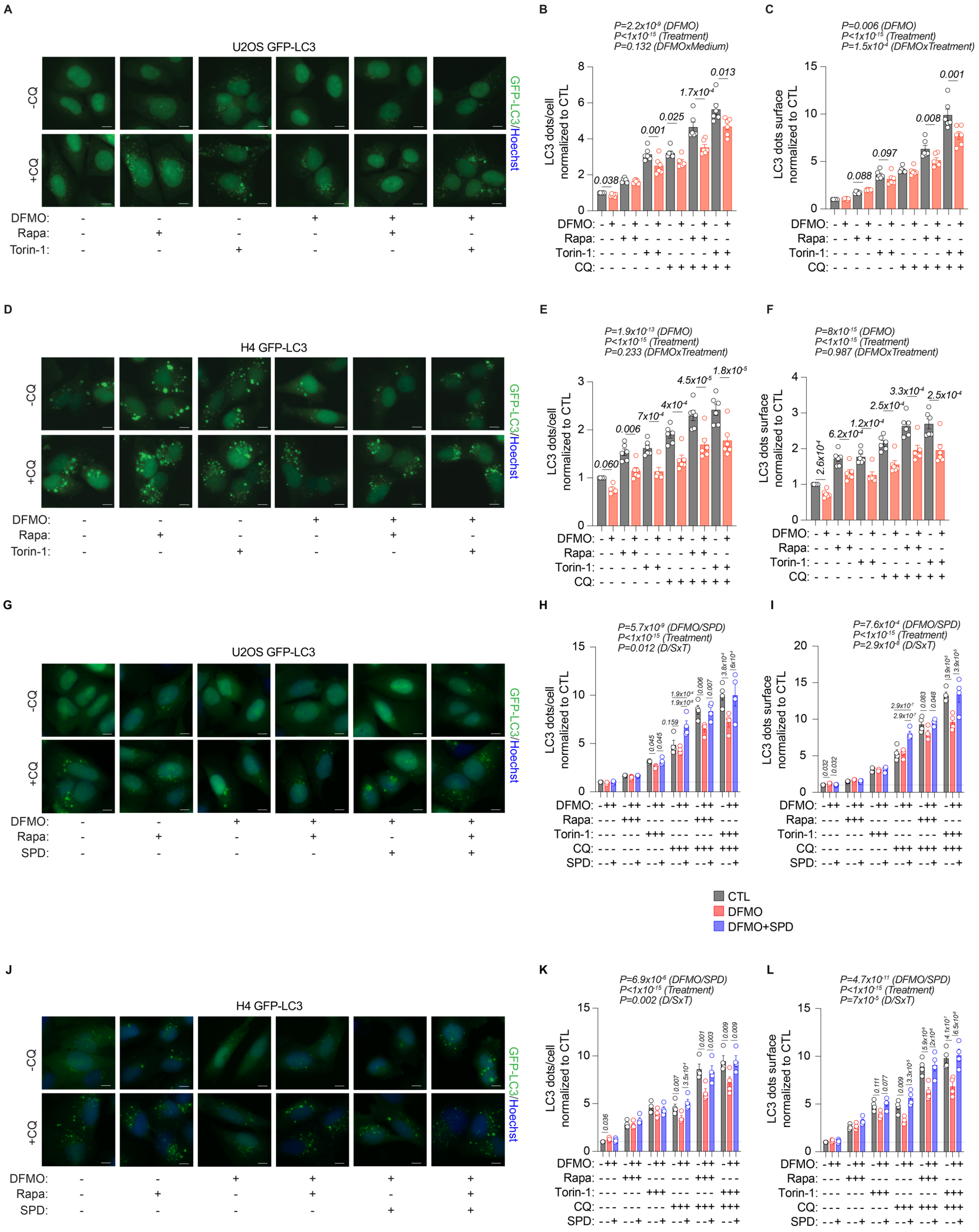

Figure S3: Spermidine is required for rapamycin-induced autophagy in human cell lines. (A) Representative images of U2OS GFP-LC3 cells treated with rapamycin (10 μM) or Torin-1 (300 nM) for 6 hours (with or without chloroquine [CQ] for 3 hours before fixation) after 3 days of 100 μM DFMO treatment. **(B-C)** Quantifications of [A]. N=6 biologically independent experiments. **(D)** Representative images of H4 GFP-LC3 cells treated with rapamycin (10 μM) or Torin-1 (300 nM) for 6 hours (with or without chloroquine [CQ] for 3 hours before fixation) after three days of 100 μM DFMO treatment.

Scale bar = 10 µm. **(E-F)** Quantifications of [D]. N=6 biologically independent experiments. **(G-I)** Representative images and quantifications of U2OS GFP-LC3 cells treated as in [A-C], combined with 10 µM SPD. 1 mM aminoguanidine was added to all conditions. N=4 biologically independent experiments. **(J-L)** Representative images and quantifications of H4 GFP-LC3 cells treated as in [A-C], combined with 10 µM SPD. 1 mM aminoguanidine was added to all conditions. N=4 biologically independent experiments. Statistics: Two-way ANOVA with Holm-Šídák's multiple comparisons test. Bar graphs show the mean ± S.E.M. Source numerical data are available in source data.

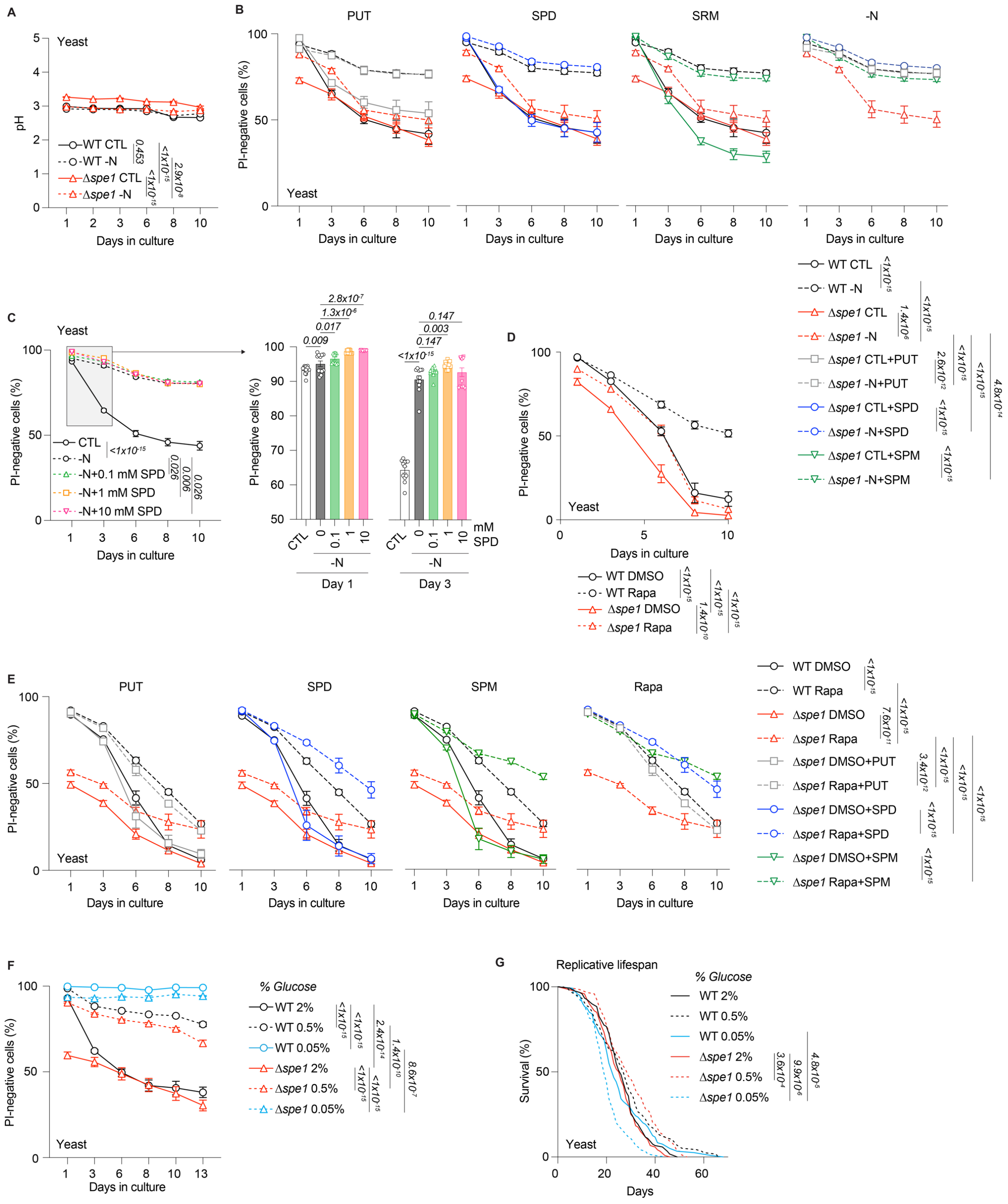

Figure S4: Spermidine is required for lifespan extension in yeast. (A) pH of the culture during chronological aging of WT BY4741 and Δ*spe1* yeast in control and -N media. N=8 biologically independent samples (yeast cultures). **(B)** Polyamine (100 μM) supplementation reinstates lifespan extension by -N in BY4741 Δ*spe1* yeast cells. N=8(WT -N; Δ*spe1* -N; Δ*spe1*+PUT, Δ*spe1*+SPD -N; Δ*spe1*+SPM -N), 9(WT, Δ*spe1*, Δ*spe1*+PUT -N; Δ*spe1*+SPD; Δ*spe1*+SPM) biologically independent samples (yeast cultures). **(C)** PI-negative (live) cells during chronological aging of yeast BY4741 WT and Δ*spe1* in control or -N media in combination with ascending SPD concentrations. N=12 biologically independent samples (yeast cultures). **(D)** Propidium iodide (PI)-negative

(live) cells during chronological aging of yeast BY4742 WT and Δ*spe1* treated with DMSO or rapamycin (40 nM) in the logarithmic growth phase. N=11 biologically independent samples (yeast cultures). **(E)** Polyamine supplementation (100 μM) normalizes rapamycin-induced (40 nM) lifespan extension in yeast BY4742 Δ*spe1*. N=9 biologically independent samples (yeast cultures). **(F)** PI-negative (live) cells during chronological aging of yeast BY4741 WT and Δ*spe1* under glucose-restricted conditions (0.5 and 0.05 %) compared to control conditions (2 % glucose). N=10 biologically independent samples (yeast cultures). **(G)** Replicative lifespan of BY4741 WT and Δ*spe1* under glucose-restricted conditions (0.5 and 0.05 %) compared to control conditions (2 % glucose). N=120 yeast cells. Statistics: [A-F] Two-way ANOVA with Holm-Šídák's multiple comparisons test. [C – single day analyses] One-way ANOVA with Holm-Šídák's multiple comparisons test. [G] Log-rank test with Bonferroni correction. Bar and line graphs show the mean ± S.E.M. Source numerical data are available in source data.

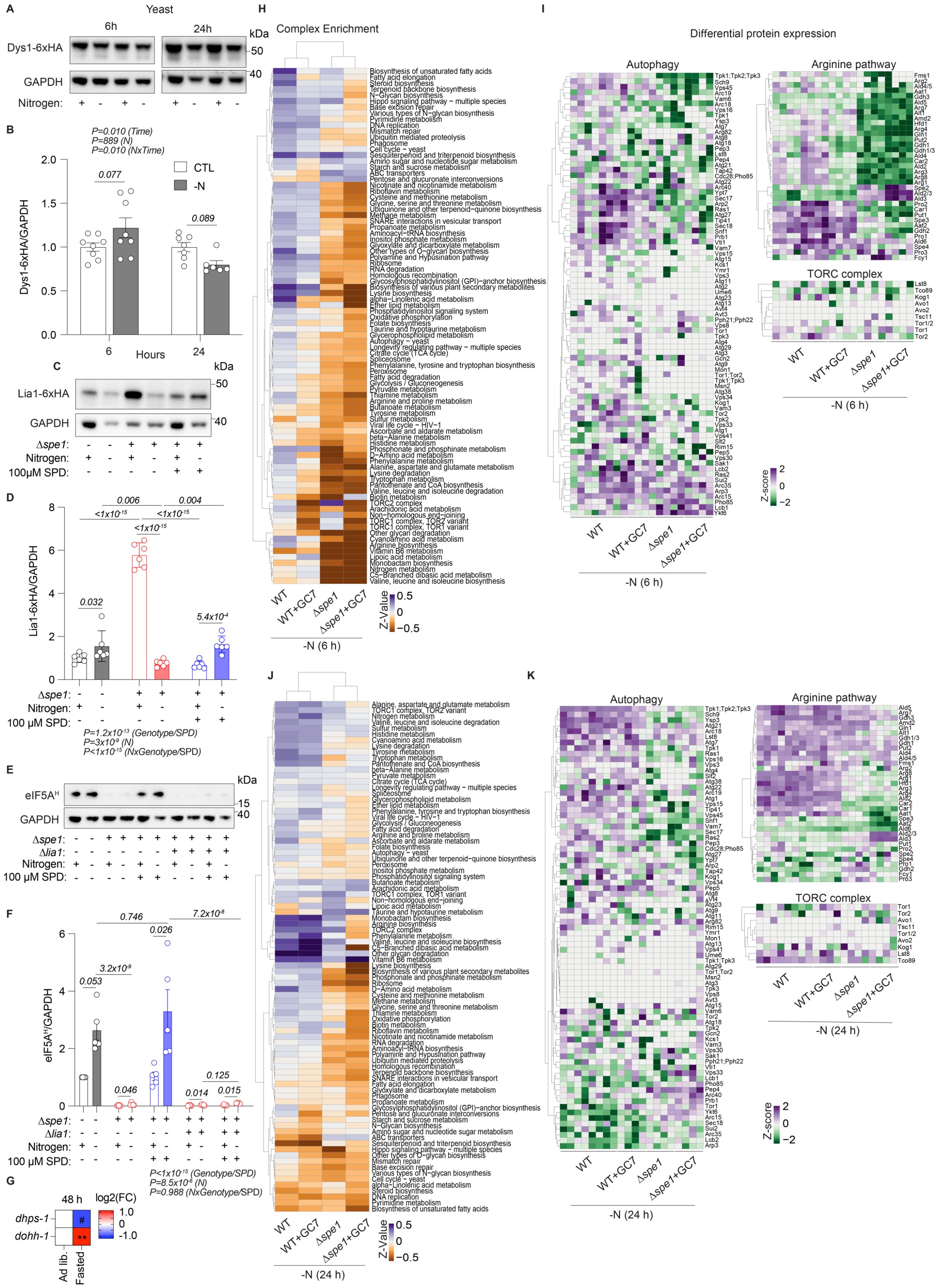

Figure S5: Fasting increases hypusination enzymes, which are required for correct TOR signaling, autophagy, and translation processes during nitrogen deprivation in yeast. (A) Representative immunoblot of yeast Dys-6xHA, assessed for HA-tags and GAPDH after 6 and 24 hours -N. **(B)** Quantification of Dys-6xHA levels as depicted in [A].

N 8(6 hours), 7(24 hours CTL), 6 (24 hours -N) biologically independent samples (yeast cultures). **(C)** Representative immunoblot of yeast Lia1-6xHA, assessed for HA-tags and GAPDH after 6 -N in WT and Δ*spe1* cells with and without 100 µM SPD. **(D)** Quantification of Lia1-6xHA levels as depicted in [C]. N=6 biologically independent samples (yeast cultures). **(E)** Representative immunoblot of yeast WT, Δ*spe1* and Δ*spe1*Δ*lia1*, assessed for hypusine and GAPDH after 6 hours -N in WT and Δ*spe1* cells with and without 100 µM SPD. **(F)** Quantification of eIF5A^{H} levels as depicted in [E]. N=5 biologically independent samples (yeast cultures). **(G)** Quantification of relative mRNA expression of *dhps-1* and *dohh-1* in 48 hours fasted *C. elegans*. N=4(*dohh-1*), 5(*dhps-1*) biologically independent experiments. **(H-K)** Proteome changes in *S. cerevisiae* WT and Δ*spe1* strains under indicated conditions following a **(H+I)** 6 hour or **(J+K)** 24-hour treatment in -N. N=8(WT), 4(rest) biologically independent samples (yeast cultures). Protein complex analyses using all annotated yeast KEGG pathways as well as complex portal annotations of the TORC complex (CPX-1715, CPX-1716, CPX-1717) visualized in a heatmap with their referring Z-Value at **(H)** 6 hours and **(J)** 24 hours. Complexes with a Z-Value >0.2/<-0.2 are shown in the heatmap, including a hierarchical clustering employing the Euclidean distance metric. Differential expression (Z score) of proteins at **(I)** 6 hours and **(K)** 24 hours involved in autophagy, arginine pathway, and TORC complex are displayed. <u>Statistics</u>: [B,D,F] Two-way ANOVA with Holm-Šídák's multiple comparisons test. [G] Two-tailed Student's *t*-test with Holm-Šídák's multiple comparisons test. Heatmaps show means. Bar graphs show the mean ± S.E.M. * *P*<0.05, ** *P*<0.01, # *P*<0.2. Source numerical data and unprocessed blots are available in source data. Proteome source numerical data are available in the PRIDE repository.

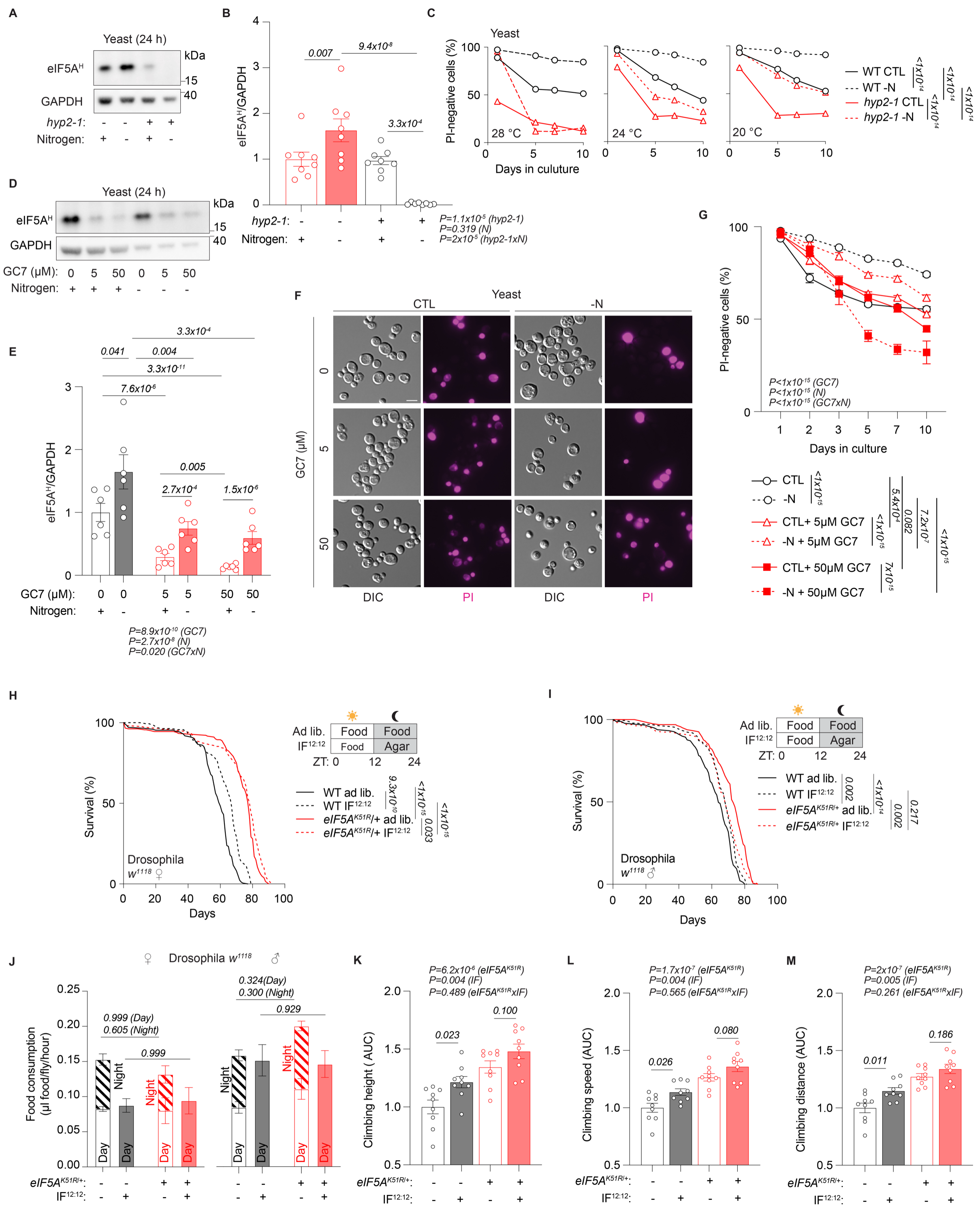

Figure S6: Genetic or pharmacological inhibition of hypusination in yeast or Drosophila curtails survival under nitrogen deprivation or IF. (A) Representative immunoblot of yeast WT and *hyp2-1*, assessed for hypusine and GAPDH after 24 hours -N. **(B)** Quantification of hypusine levels as depicted in [A]. N=8 biologically independent samples (yeast cultures). **(C)** Chronological aging of yeast WT and *hyp2-1* (temperature-sensitive mutant, hyp2 = eIF5A homolog) in CTL and -N medium at different temperatures. N=8 biologically independent samples (yeast cultures). **(D)** Representative immunoblot of yeast cells treated with 5 or 50 µM GC7, assessed for

hypusine and GAPDH after 24 hours -N. **(E)** Quantification of hypusine levels as depicted in [D]. N=6 biologically independent samples (yeast cultures). **(F)** Representative images on day 5 of chronological aging experiments of yeast WT cells treated with 5 or 50 µM GC7, stained with PI and quantified in [G]. Scale bar = 5 µm. **(G)** GC7 treatment reduces survival, measured as PI-negative cells (live), of yeast WT cells in -N. N = 8(day 10), 19(CTL+50µM d5/d7, 18(-N d7), 20(rest). **(H-I)** Lifespan of isogenic female and male w^{1118} flies and heterozygous *eIF5A*^{K51R}/+ flies during IF$^{12:12}$. **(H)** Lifespan analysis of female flies. N=173(WT ad lib.), 168(WT IF), 183(eIF5A$^{K51R/+}$ ad lib.), 175(eIF5A$^{K51R/+}$ IF) flies. **(I)** Lifespan analysis of male flies. N=180(WT ad lib.), 191(WT IF), 168(eIF5A$^{K51R/+}$ ad lib.), 166 (eIF5A$^{K51R/+}$ IF) flies. **(J)** Food consumption of 10-day old female and male flies during the first 7 cycles of IF. N=9(WT, *eIF5A*^{K51R}/+ IF Day), 6(*eIF5A*^{K51R}/+ ad lib. Day, *eIF5A*^{K51R}/+ ad lib. Night) biologically independent samples (groups of 5 flies per N). **(K-M)** Flies from [H] were assessed for their climbing ability, measured as reached height, speed, and covered walking distance after a negative geotaxis stimulus, on day 35 of the IF$^{12:12}$ aging experiments. N=9 biologically independent samples (groups of flies). Statistics: [B-E,G,K-M] Two-way ANOVA with Holm-Šídák's multiple comparisons test. [H,I] Log-rank test with Bonferroni correction. [J] Two-tailed unpaired Student's *t*-test. Bar and line graphs show the mean ± S.E.M. Source numerical data and unprocessed blots are available in source data.

Uncropped Immunoblots for Supplementary Figures

Figure S5A

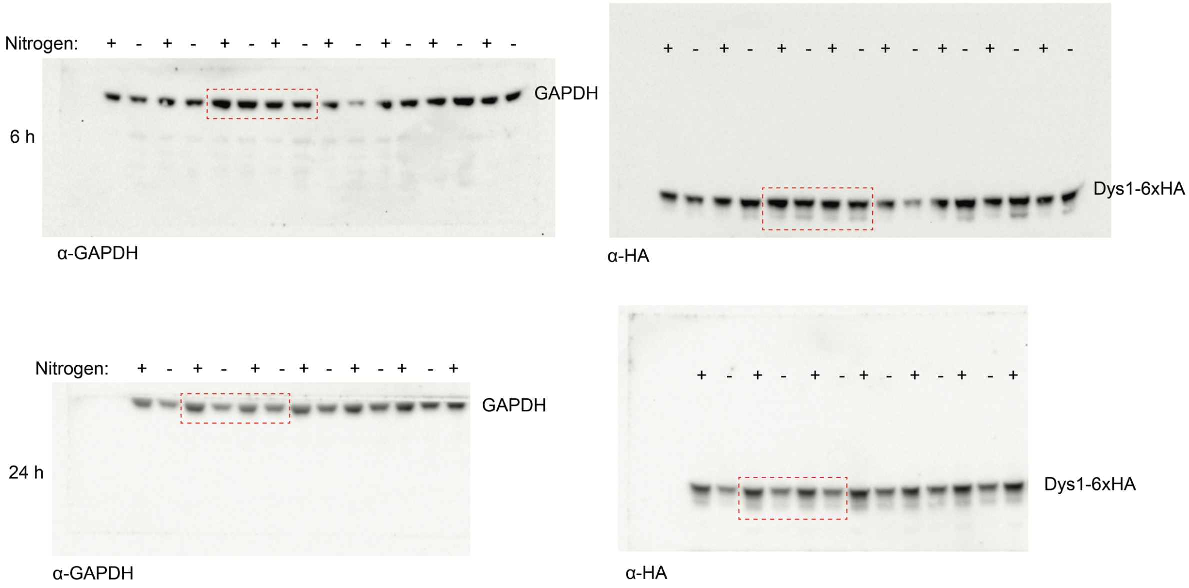

Figure S5C

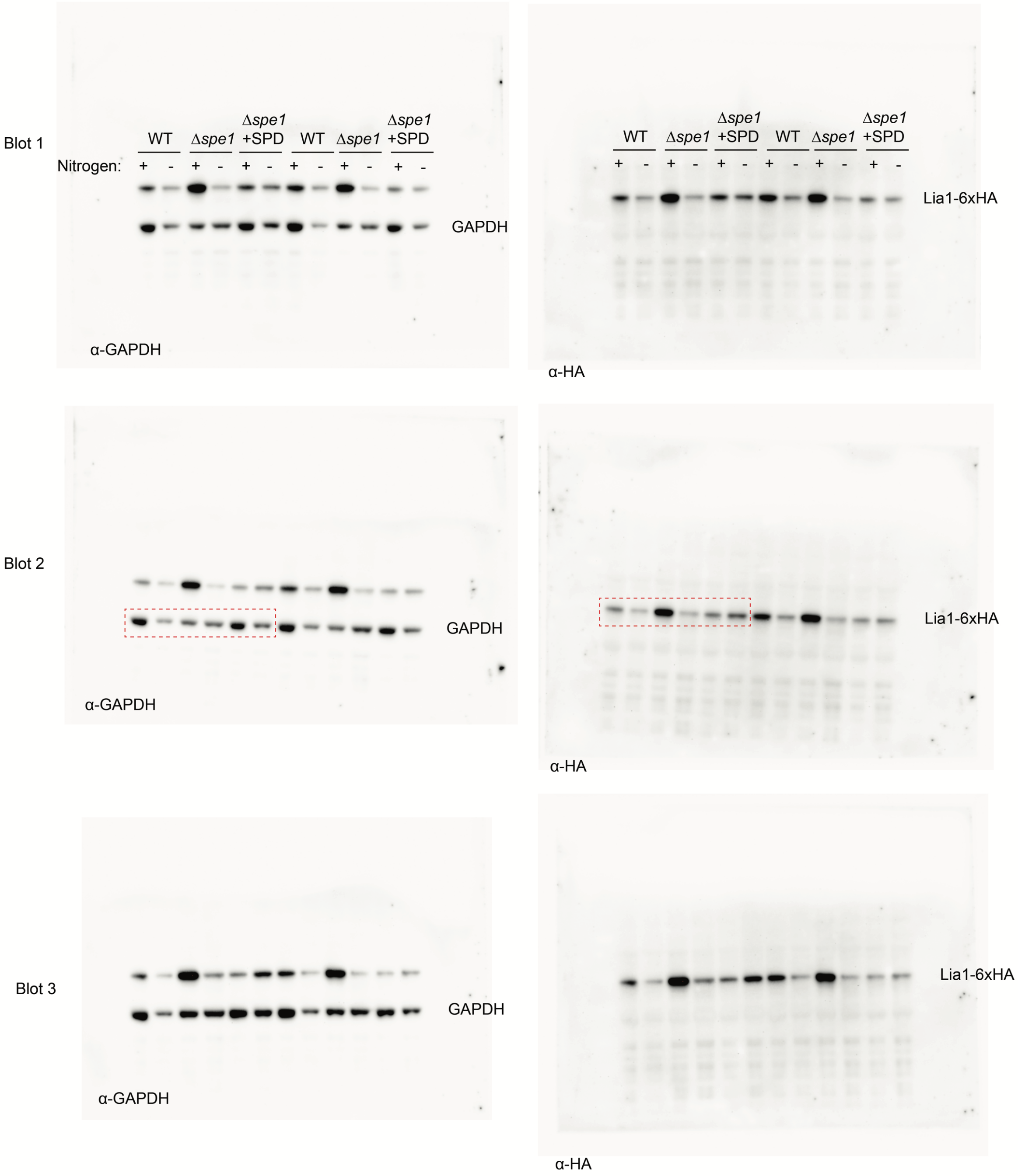
Blot 1
WT
Δspe1
Δspe1 +SPD
WT
Δspe1
Δspe1 +SPD
Nitrogen: + - + - + - + - + - + -
GAPDH
α-GAPDH
WT
Δspe1
Δspe1 +SPD
WT
Δspe1
Δspe1 +SPD
+ - + - + - + - + - + -
Lia1-6xHA
α-HA
Blot 2
GAPDH
α-GAPDH
Lia1-6xHA
α-HA
Blot 3
GAPDH
α-GAPDH
Lia1-6xHA
α-HA

Figure S5E

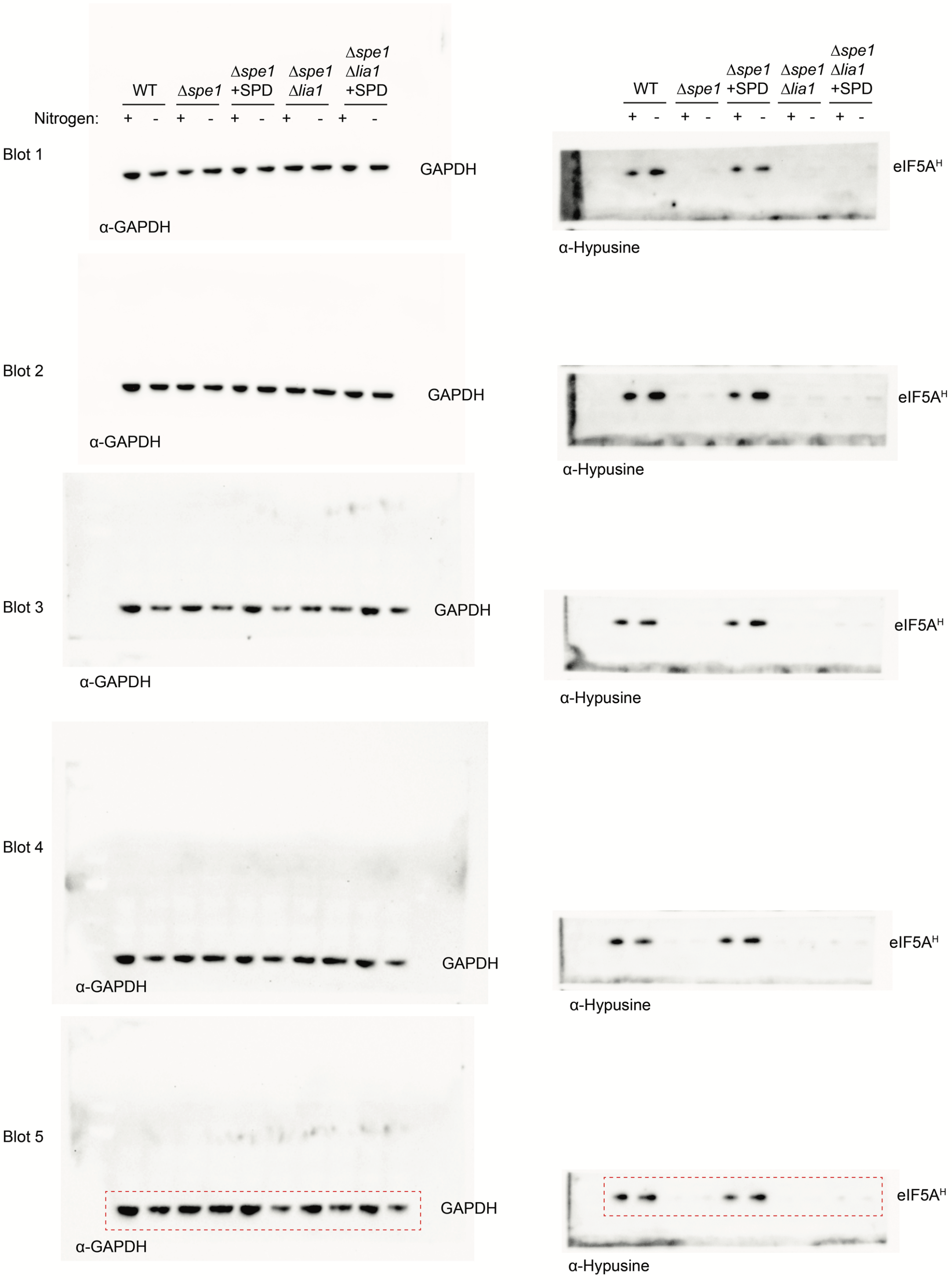
WT
Δspe1
Δspe1 +SPD
Δspe1 Δlia1
Δspe1 Δlia1 +SPD
Nitrogen:
+ - + - + - + - + -
Blot 1
GAPDH
α-GAPDH
eIF5A^{H}
α-Hypusine
Blot 2
GAPDH
α-GAPDH
eIF5A^{H}
α-Hypusine
Blot 3
GAPDH
α-GAPDH
eIF5A^{H}
α-Hypusine
Blot 4
GAPDH
α-GAPDH
eIF5A^{H}
α-Hypusine
Blot 5
GAPDH
α-GAPDH
eIF5A^{H}
α-Hypusine

Figure S6A

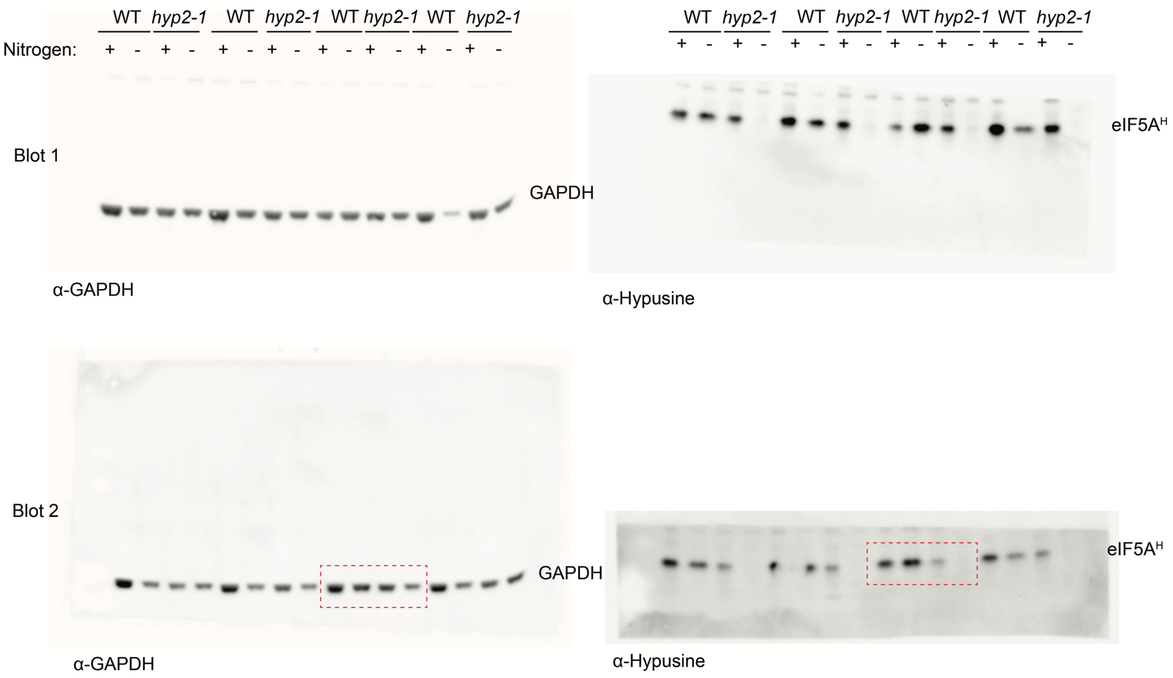
WT hyp2-1 WT hyp2-1 WT hyp2-1 WT hyp2-1
Nitrogen: + - + - + - + - + - + - + - + -
Blot 1
GAPDH
α-GAPDH
eIF5AH
α-Hypusine
Blot 2
GAPDH
α-GAPDH
eIF5AH
α-Hypusine

Figure S6D

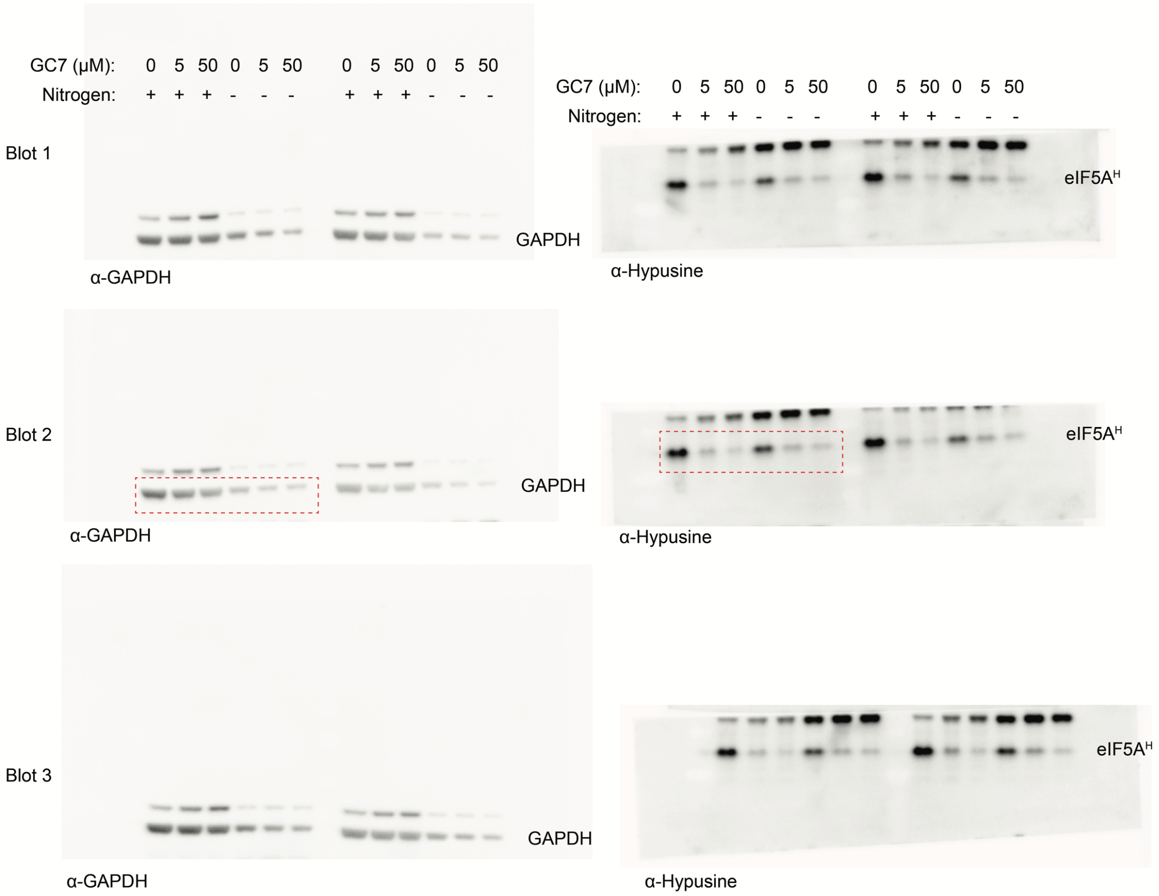
GC7 (μM): 0 5 50 0 5 50 0 5 50 0 5 50
Nitrogen: + + + - - - + + + - - -
Blot 1
GAPDH
α-GAPDH
eIF5A^H
α-Hypusine
Blot 2
GAPDH
α-GAPDH
eIF5A^H
α-Hypusine
Blot 3
GAPDH
α-GAPDH
eIF5A^H
α-Hypusine
