# Supplementary material for: Spermidine is essential for fasting-mediated autophagy and longevity
Source: Nat Cell Biol. 2024 Aug 8;26(9):1571–84. doi: 10.1038/s41556-024-01468-x (PMC11392816; doi:10.1038/s41556-024-01468-x)
Supplement: Supplementary file 2 — Reporting Summary [file 41556_2024_1468_MOESM2_ESM.pdf]

nature portfolio

Corresponding author(s): Frank Madeo

Last updated by author(s): Jun 13, 2024

Reporting Summary

Nature Portfolio wishes to improve the reproducibility of the work that we publish. This form provides structure for consistency and transparency in reporting. For further information on Nature Portfolio policies, see our Editorial Policies and the Editorial Policy Checklist.

Statistics

For all statistical analyses, confirm that the following items are present in the figure legend, table legend, main text, or Methods section.

n/a	Confirmed	
☐	☒	The exact sample size (*n*) for each experimental group/condition, given as a discrete number and unit of measurement
☐	☒	A statement on whether measurements were taken from distinct samples or whether the same sample was measured repeatedly
☐	☒	The statistical test(s) used AND whether they are one- or two-sided<br>*Only common tests should be described solely by name; describe more complex techniques in the Methods section.*
☒	☐	A description of all covariates tested
☐	☒	A description of any assumptions or corrections, such as tests of normality and adjustment for multiple comparisons
☐	☒	A full description of the statistical parameters including central tendency (e.g. means) or other basic estimates (e.g. regression coefficient) AND variation (e.g. standard deviation) or associated estimates of uncertainty (e.g. confidence intervals)
☐	☒	For null hypothesis testing, the test statistic (e.g. *F*, *t*, *r*) with confidence intervals, effect sizes, degrees of freedom and *P* value noted<br>*Give P values as exact values whenever suitable.*
☒	☐	For Bayesian analysis, information on the choice of priors and Markov chain Monte Carlo settings
☒	☐	For hierarchical and complex designs, identification of the appropriate level for tests and full reporting of outcomes
☒	☐	Estimates of effect sizes (e.g. Cohen's *d*, Pearson's *r*), indicating how they were calculated

Our web collection on statistics for biologists contains articles on many of the points above.

Software and code

Policy information about availability of computer code

Data collection	MetaXpress (Molecular Devices), Image Lab (BD), FACSDiva (BD),
Data analysis	MetaXpress (Molecular Devices), ImageJ (2.3.0), Graphpad Prism 10.1.0, Metaboanalyst 5.0, Chenomx NMR Suite 8.4 (Chenomx Inc.), Matlab 2014b, Microsoft Excel for Mac and Windows (Version 16.84), ImageLab 5.2 (Bio-Rad), DIA-NN (v1.8.0), Xcalibur v4.0 and v2.2 Software (Thermo Fisher Scientific).

For manuscripts utilizing custom algorithms or software that are central to the research but not yet described in published literature, software must be made available to editors and reviewers. We strongly encourage code deposition in a community repository (e.g. GitHub). See the Nature Portfolio guidelines for submitting code & software for further information.

Data

Policy information about availability of data

All manuscripts must include a data availability statement. This statement should provide the following information, where applicable:

- Accession codes, unique identifiers, or web links for publicly available datasets
- A description of any restrictions on data availability
- For clinical datasets or third party data, please ensure that the statement adheres to our policy

The mass spectrometry yeast proteomics data have been deposited to the ProteomeXchange Consortium via the PRIDE (Http://www.ebi.ac.uk/pride) partner

repository with the dataset identifier PXD035909. The biological material and datasets, including NMR and MS metabolomics data, generated and/or analyzed during the current study are in the source data or available from the corresponding authors on reasonable request.

Research involving human participants, their data, or biological material

Policy information about studies with human participants or human data. See also policy information about sex, gender (identity/presentation), and sexual orientation and race, ethnicity and racism.

Reporting on sex and gender	The main findings of increased spermidine levels and hypusine upon fasting in humans occur irrespective of the participants' sex. Sex was determined by self-reporting and gender data was not collected. Due to the implications of these analyses, we did not perform sex-stratified analyses for the rest of the analyses.
Reporting on race, ethnicity, or other socially relevant groupings	We only used self-reported sex as a category. We did not use other categories or factors in our analyses of human samples. All other details are listed in Table S1 and the respective publications.
Population characteristics	Population characteristics for each cohort are listed in Table S1 and the respective publications.
Recruitment	Recruitment strategies are listed in the specific methods section of each cohort and/or in the respective publications. Selection bias is possible due to the participant's self-interest in fasting interventions.
Ethics oversight	As stated in the Materials & Methods section. Medical council of Baden-Württemberg, Germany; Ethics Committee of the Charité-University Medical Center Berlin, Germany; Ethics committee of the Medical University of Graz, Austria; Institutional review board of Charité Universitätsmedizin Berlin, Germany

Note that full information on the approval of the study protocol must also be provided in the manuscript.

Field-specific reporting

Please select the one below that is the best fit for your research. If you are not sure, read the appropriate sections before making your selection.

☒ Life sciences ☐ Behavioural & social sciences ☐ Ecological, evolutionary & environmental sciences

For a reference copy of the document with all sections, see nature.com/documents/nr-reporting-summary-flat.pdf

Life sciences study design

All studies must disclose on these points even when the disclosure is negative.

Sample size	Sample sizes were based on common and feasible sample sizes used in the respective field of research, without performing power analysis. Metabolite measurements of human samples relied on pre-established cohorts, that were collected for primary endpoints as described in the respective publication, study protocol or clinical registry entry (see Table S1).
Data exclusions	Outlier analysis by the ROUT method using Graphpad Prism 10.1.0 and outlier exclusion were performed exclusively on the metabolite measurements of human biological samples. Data exclusion criteria were not pre-established.
Replication	Appropriate numbers of independent biological samples and independent experimental repeats were used in all experiments, varying based on the model organism and technical requirements. All data points shown are independent samples. All experimental replications yielded comparable outcomes.
Randomization	Allocation to the experimental groups was random. Randomization procedures for human participants are described in detail in the respective publications and study protocols (see Table S1).
Blinding	Worms, flies and mice were randomly assigned to the experimental groups, but the experimenters were not blinded, except for the measurement of mouse healthspan (echocardiography, frailty index, body composition, wire hanging capability, grip strength, body surface temperature, etc.). During continuous (intermittent) fasting experiments it is not possible to blind the person handling the experiment due to obvious reasons. Also, the manual assessment of yeast GFP-Atg8 microscopy was performed in a blinded fashion. All other experiments were conducted in a non-blinded manner.

Reporting for specific materials, systems and methods

We require information from authors about some types of materials, experimental systems and methods used in many studies. Here, indicate whether each material, system or method listed is relevant to your study. If you are not sure if a list item applies to your research, read the appropriate section before selecting a response.

Materials & experimental systems

n/a	Involved in the study
☐	☒ Antibodies
☐	☒ Eukaryotic cell lines
☒	☐ Palaeontology and archaeology
☐	☒ Animals and other organisms
☐	☒ Clinical data
☒	☐ Dual use research of concern
☒	☐ Plants

Methods

n/a	Involved in the study
☒	☐ ChIP-seq
☒	☐ Flow cytometry
☒	☐ MRI-based neuroimaging

Antibodies

Antibodies used	anti-GFP (Roche #1814460), anti-hypusine (Merck #ABS1064-I), anti-eIF5A (BD #611977), anti-GAPDH clone GA1R (ThermoFisher Scientific #MA5-15738), anti-HA (Sigma #H 9658), anti-mouse IgG (Sigma #A9044), anti-rabbit IgG (Sigma #A0545), Sch9-pThr737 (custom), anti-Sch9 (custom), anti-Adh1 (Calbiochem, #126745), HRP-linked anti-rabbit IgG (Sigma #A0545), anti-beta actin (abcam #ab197277), anti-rabbit Cy5 (Invitrogen), goat anti-guinea pig Alexa 555 (Invitrogen)
Validation	Antibodies were validated by the manufacturers or internally by protein-lowering strategies (e.g. pharmacological inhibition of hypusination for anti-hypusine)

Eukaryotic cell lines

Policy information about cell lines and Sex and Gender in Research

Cell line source(s)	human glioblastoma H4 (ATCC), human osteosarcoma U2OS (ATCC)
Authentication	Cell lines were not authenticated.
Mycoplasma contamination	Cell lines were mycoplasma-free.
Commonly misidentified lines (See ICLAC register)	No commonly misidentified lines were used in this study.

Animals and other research organisms

Policy information about studies involving animals; ARRIVE guidelines recommended for reporting animal research, and Sex and Gender in Research

Laboratory animals	male and female 8-12 weeks old BALB/CjRj mice (Janvier Labs); 4, 9, 16-17 and 20 months old C57BL/6J:Rj mice (Janiver Labs); transgenic male IGF1Rtg mice and male dnPI3K mice up to 9 months of age; serum of transgenic K/BxN mice; male and female w1118 Drosophila melanogaster at varying ages across their lifespan; male and female heterozygous Odc1MI10996/+ loss-of-function mutant flies (BDSC #56103) at varying ages across their lifespan; male and female heterozygous eIF5AK51R/+ flies at varying ages across their lifespan;
Wild animals	The study did not involve wild animals.
Reporting on sex	Since sex did not have an impact on spermidine levels during fasting in humans, we did not consider sex in the pre-clinical study design for all analyses. The sex of the animals was explicitly stated in the figure legends, Materials & Methods section and the main text. We used male and female mice for metabolomics (separately). The investigation of the cardioprotective and healthspan-promoting effects of intermittent fasting were performed on male mice only. We used a 1:1 mixed-sex cohort for the investigation of intermittent fasting on an arthritis model, analysed together and per sex.
Field-collected samples	The study did not involve samples collected from the field.
Ethics oversight	All animal experiments were performed in accordance with national and European ethical regulation (Directive 2010/63/EU) and approved by the responsible government agencies (Bundesministerium für Wissenschaft, Forschung und Wirtschaft, BMWFW; BMWFW-66.007/0029-WF/V/3b/2017, GZ 2021-0.524.242, GZ 2022-0.137.213, BMWFW-66.010/0160-WF/V/3b/2014, BMWFW-66.010/0198-WF/V/3b/2017, BMBWF-66.010/0042-V/3b/2018). Animal studies using protocol 3 (IF24:24) and the arthritis model were approved by the local ethical committee (District Government of Lower Franconia, #55.2-2532-2-1041-15). All experiments were conducted according to the guidelines of the Federation of European Laboratory Animal Science Associations (FELASA). Calorie-restricted mice (protocol 4) were used in accordance with protocols approved by the Institutional Animal Care and Use Committee of the NIA.

Note that full information on the approval of the study protocol must also be provided in the manuscript.

Clinical data

Policy information about clinical studies

All manuscripts should comply with the ICMJE guidelines for publication of clinical research and a completed CONSORT checklist must be included with all submissions.

Clinical trial registration	DRKS00016657, DRKS00010111 (German Clinical Trials Register), NCT02673515, NCT 04739852 (Clinicaltrials.gov)
Study protocol	Published study protocols are listed in Table S1, where available. For the rest we refer to the clinical trial registries and previous publications.
Data collection	Details of recruitment strategies and data collection are described in the referenced publications and clinical trial registry entries.
Outcomes	None of the herein described metabolite measurements are part of primary endpoints in the respective trials. Primary endpoints of these trials have been published elsewhere (Table S1 summarizes previous publications).

Plants

Seed stocks	*Report on the source of all seed stocks or other plant material used. If applicable, state the seed stock centre and catalogue number. If plant specimens were collected from the field, describe the collection location, date and sampling procedures.*
Novel plant genotypes	*Describe the methods by which all novel plant genotypes were produced. This includes those generated by transgenic approaches, gene editing, chemical/radiation-based mutagenesis and hybridization. For transgenic lines, describe the transformation method, the number of independent lines analyzed and the generation upon which experiments were performed. For gene-edited lines, describe the editor used, the endogenous sequence targeted for editing, the targeting guide RNA sequence (if applicable) and how the editor was applied.*
Authentication	*Describe any authentication procedures for each seed stock used or novel genotype generated. Describe any experiments used to assess the effect of a mutation and, where applicable, how potential secondary effects (e.g. second site T-DNA insertions, mosiacism, off-target gene editing) were examined.*
