# Supplementary material for: Spermidine is essential for fasting-mediated autophagy and longevity
Source: Nat Cell Biol. 2024 Aug 8;26(9):1571–84. doi: 10.1038/s41556-024-01468-x (PMC11392816; doi:10.1038/s41556-024-01468-x)
Supplement: Supplementary file 14 — Uncropped western blots. [file 41556_2024_1468_MOESM14_ESM.pdf]

Figure 3A

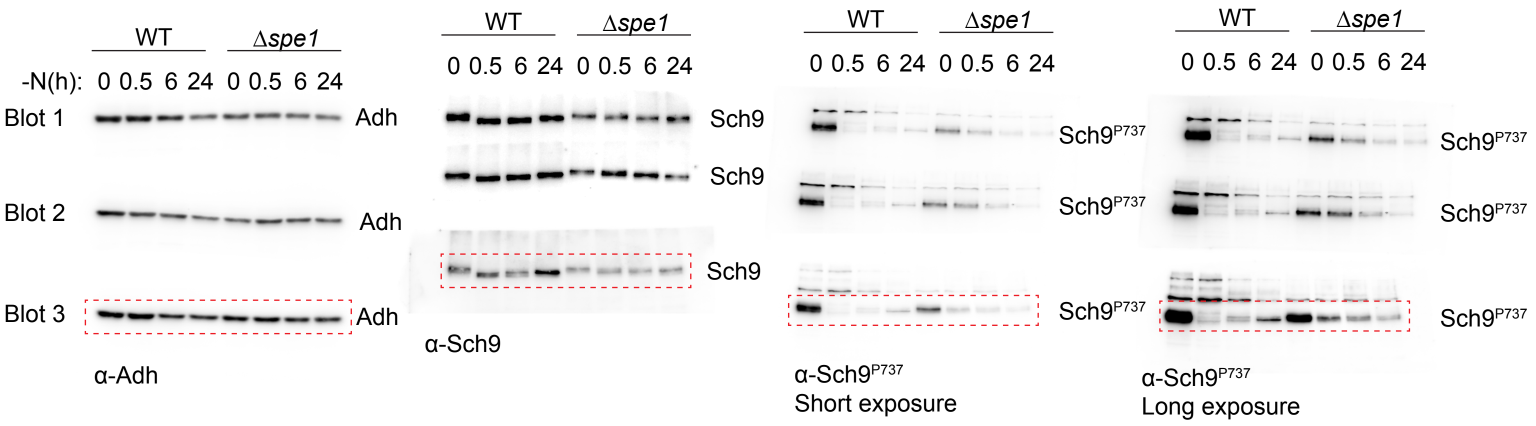
WT
Δspe1
-N(h): 0 0.5 6 24 0 0.5 6 24
Blot 1
Blot 2
Blot 3
Adh
α-Adh
Sch9
α-Sch9
Sch9P737
α-Sch9P737
Short exposure
Long exposure

Figure 3D

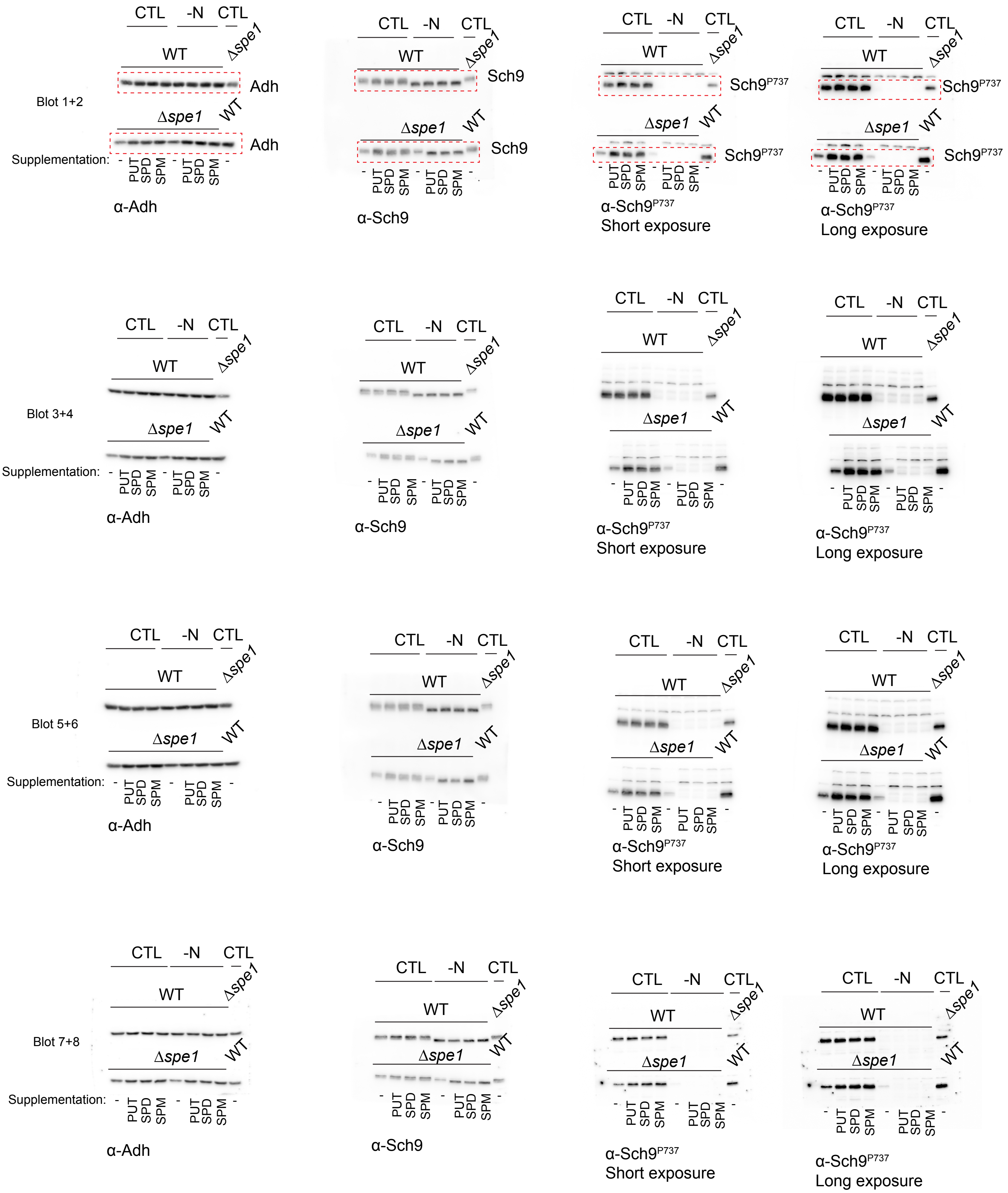
CTL -N CTL
WT Δspe1
Adh
Blot 1+2
Δspe1 WT
Adh
Supplementation: - PUT SPD SPM - PUT SPD SPM -
α-Adh
CTL -N CTL
WT Δspe1
Sch9
Δspe1 WT
Sch9
- PUT SPD SPM - PUT SPD SPM -
α-Sch9
CTL -N CTL
WT Δspe1
Sch9P737
Δspe1 WT
Sch9P737
- PUT SPD SPM - PUT SPD SPM -
α-Sch9P737
Short exposure
CTL -N CTL
WT Δspe1
Sch9P737
Δspe1 WT
Sch9P737
- PUT SPD SPM - PUT SPD SPM -
α-Sch9P737
Long exposure
CTL -N CTL
WT Δspe1
Blot 3+4
Δspe1 WT
Supplementation: - PUT SPD SPM - PUT SPD SPM -
α-Adh
CTL -N CTL
WT Δspe1
Δspe1 WT
- PUT SPD SPM - PUT SPD SPM -
α-Sch9
CTL -N CTL
WT Δspe1
Δspe1 WT
- PUT SPD SPM - PUT SPD SPM -
α-Sch9P737
Short exposure
CTL -N CTL
WT Δspe1
Δspe1 WT
- PUT SPD SPM - PUT SPD SPM -
α-Sch9P737
Long exposure
CTL -N CTL
WT Δspe1
Blot 5+6
Δspe1 WT
Supplementation: - PUT SPD SPM - PUT SPD SPM -
α-Adh
CTL -N CTL
WT Δspe1
Δspe1 WT
- PUT SPD SPM - PUT SPD SPM -
α-Sch9
CTL -N CTL
WT Δspe1
Δspe1 WT
- PUT SPD SPM - PUT SPD SPM -
α-Sch9P737
Short exposure
CTL -N CTL
WT Δspe1
Δspe1 WT
- PUT SPD SPM - PUT SPD SPM -
α-Sch9P737
Long exposure
CTL -N CTL
WT Δspe1
Blot 7+8
Δspe1 WT
Supplementation: - PUT SPD SPM - PUT SPD SPM -
α-Adh
CTL -N CTL
WT Δspe1
Δspe1 WT
- PUT SPD SPM - PUT SPD SPM -
α-Sch9
CTL -N CTL
WT Δspe1
Δspe1 WT
- PUT SPD SPM - PUT SPD SPM -
α-Sch9P737
Short exposure
CTL -N CTL
WT Δspe1
Δspe1 WT
- PUT SPD SPM - PUT SPD SPM -
α-Sch9P737
Long exposure

Figure 3E

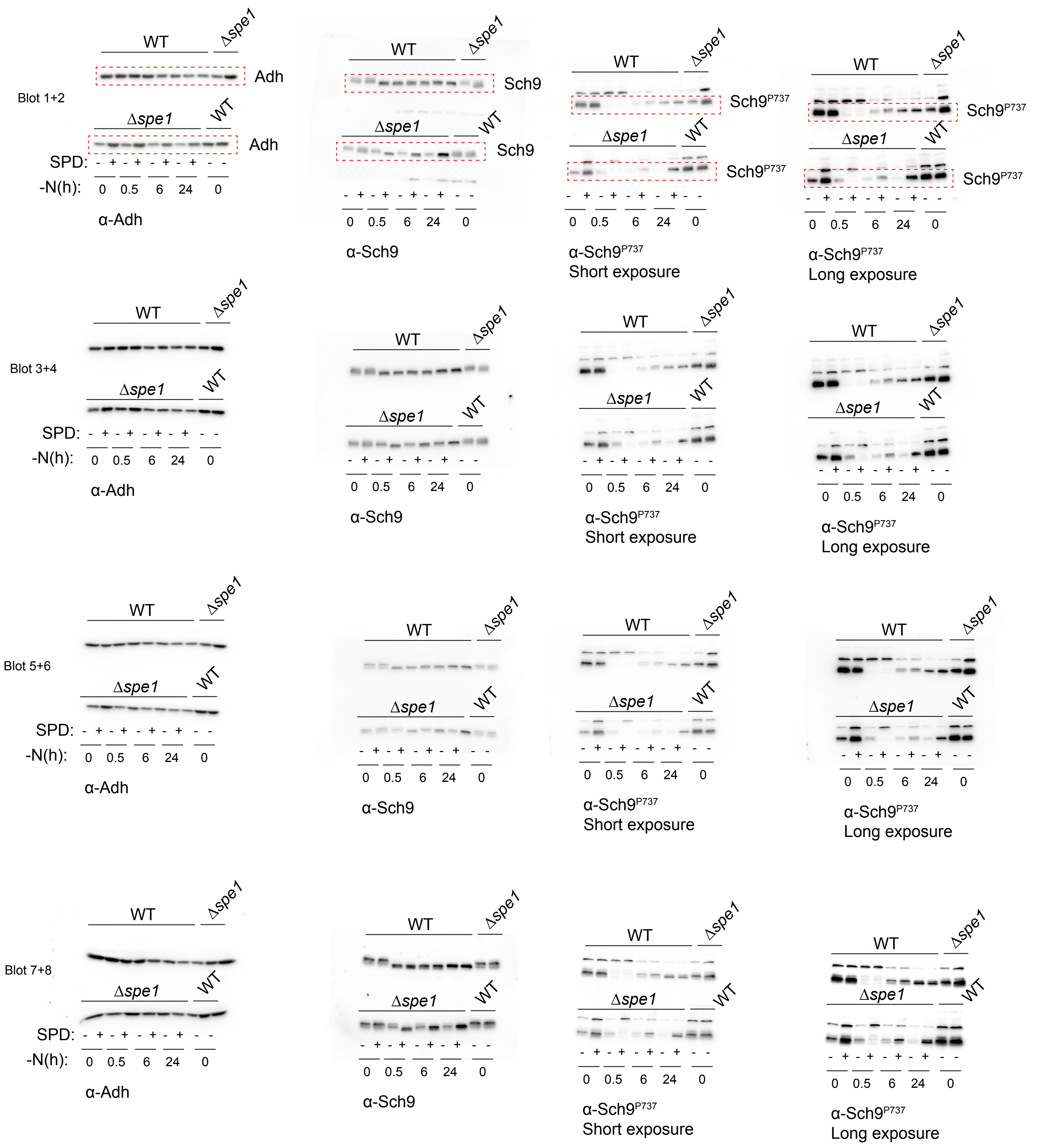

Blot 1+2
WT
Δspe1
Adh
SPD: - + - + - + - + - -
-N(h): 0 0.5 6 24 0
α-Adh
Sch9
α-Sch9
Sch9P737
α-Sch9P737
Short exposure
Long exposure
Blot 3+4
Blot 5+6
Blot 7+8

Figure 3H

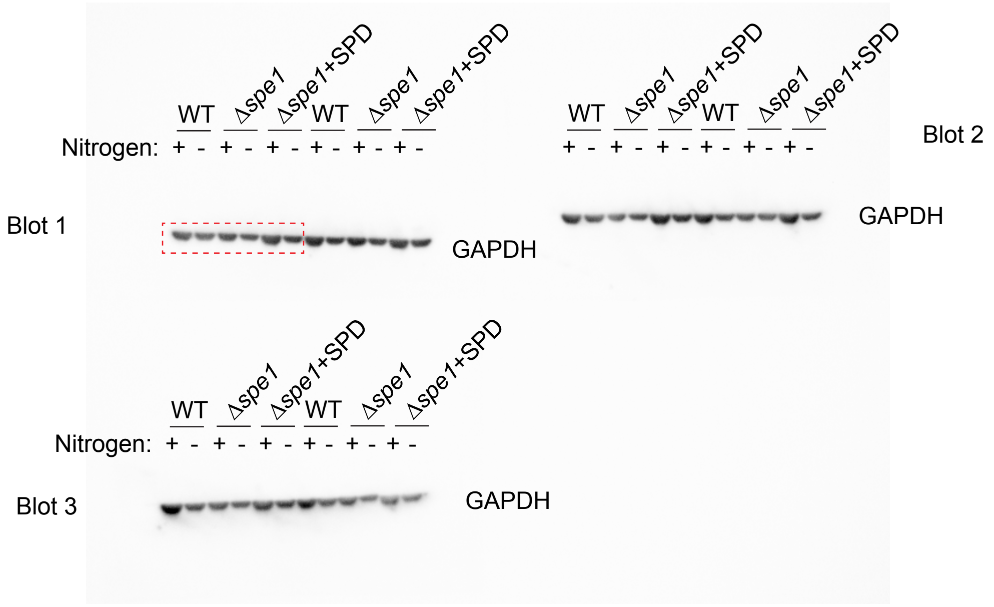

α-GAPDH

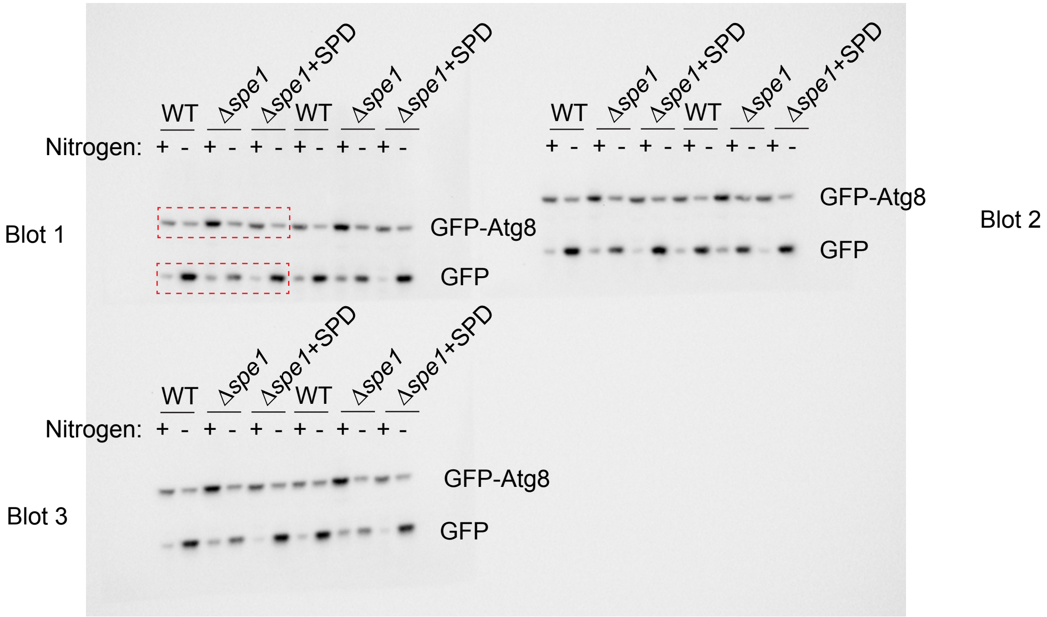

α-GFP

Figure 3K

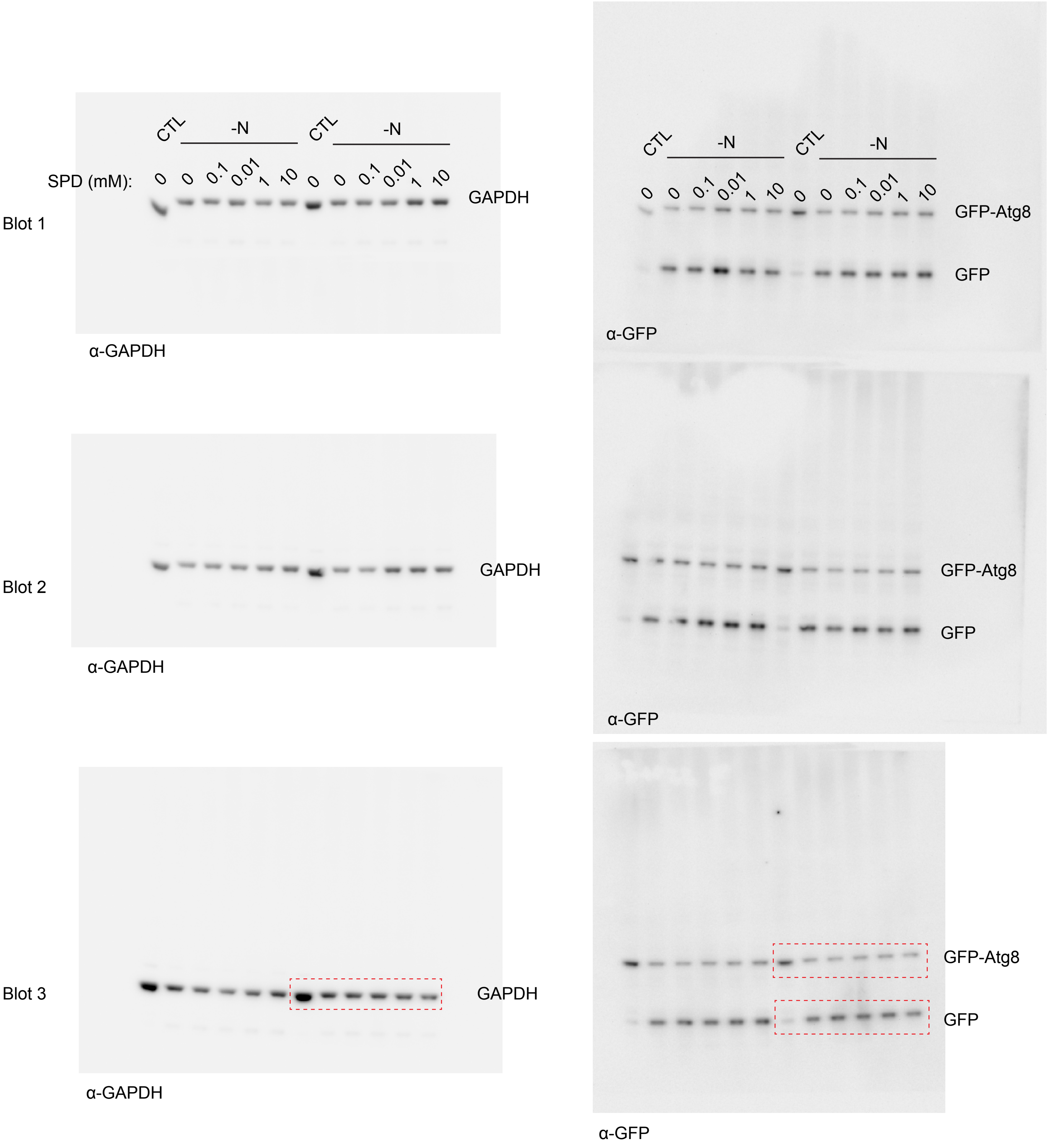

The lower parts of these blots were probed for α-hypusine in Extended Data Fig. 9E and therefore share the α-GAPDH images.
