# Supplementary figures and images for: Spermidine is essential for fasting-mediated autophagy and longevity
Source: Nat Cell Biol. 2024 Aug 8;26(9):1571–84. doi: 10.1038/s41556-024-01468-x (PMC11392816; doi:10.1038/s41556-024-01468-x)

Figure 6B

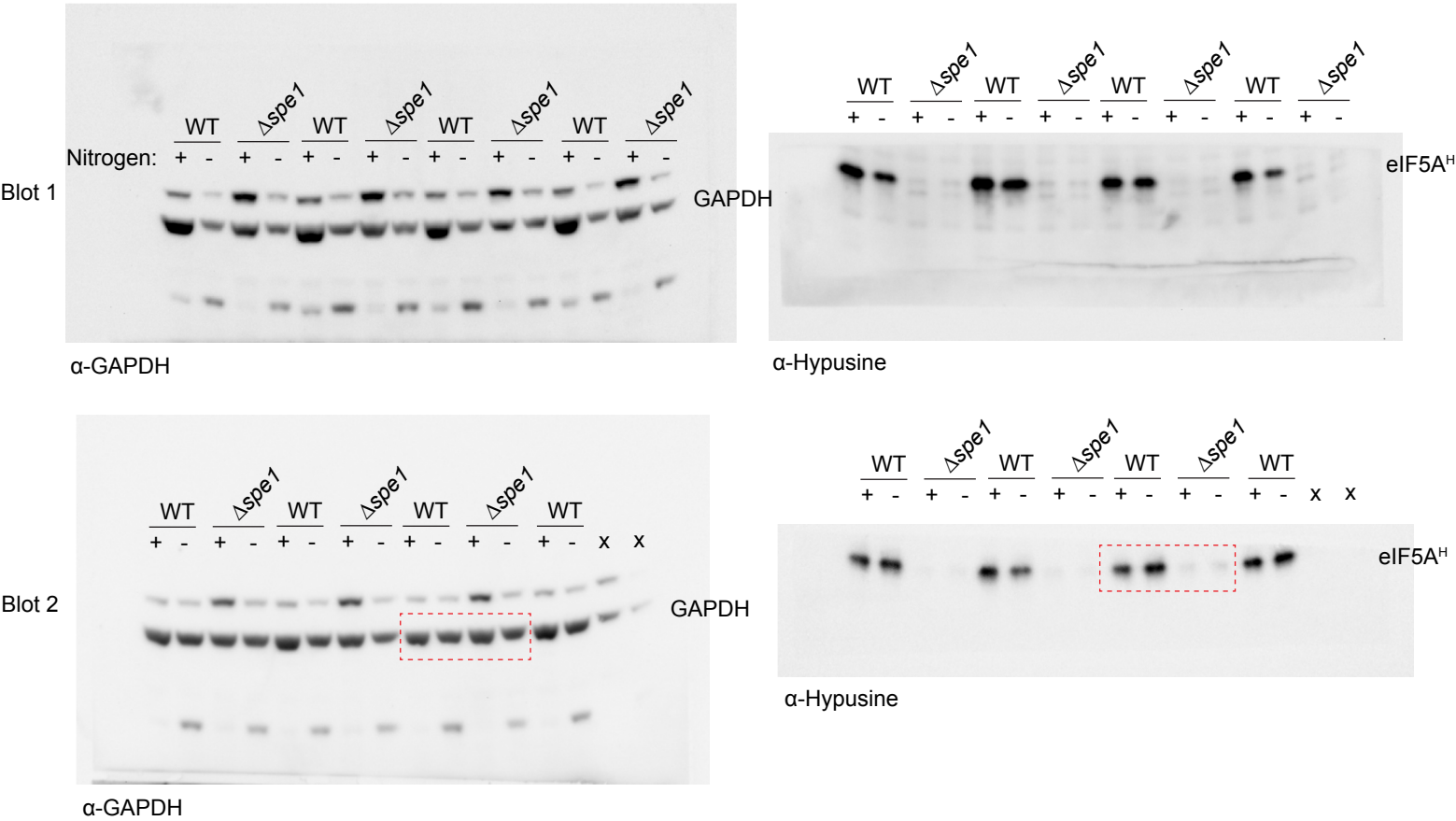

Figure 6F

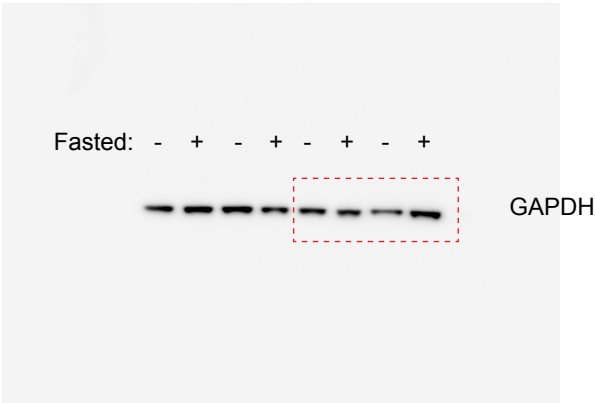

$\alpha$ -GAPDH

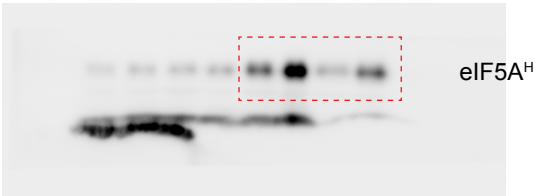

$\alpha$ -Hypusine

Figure 6H

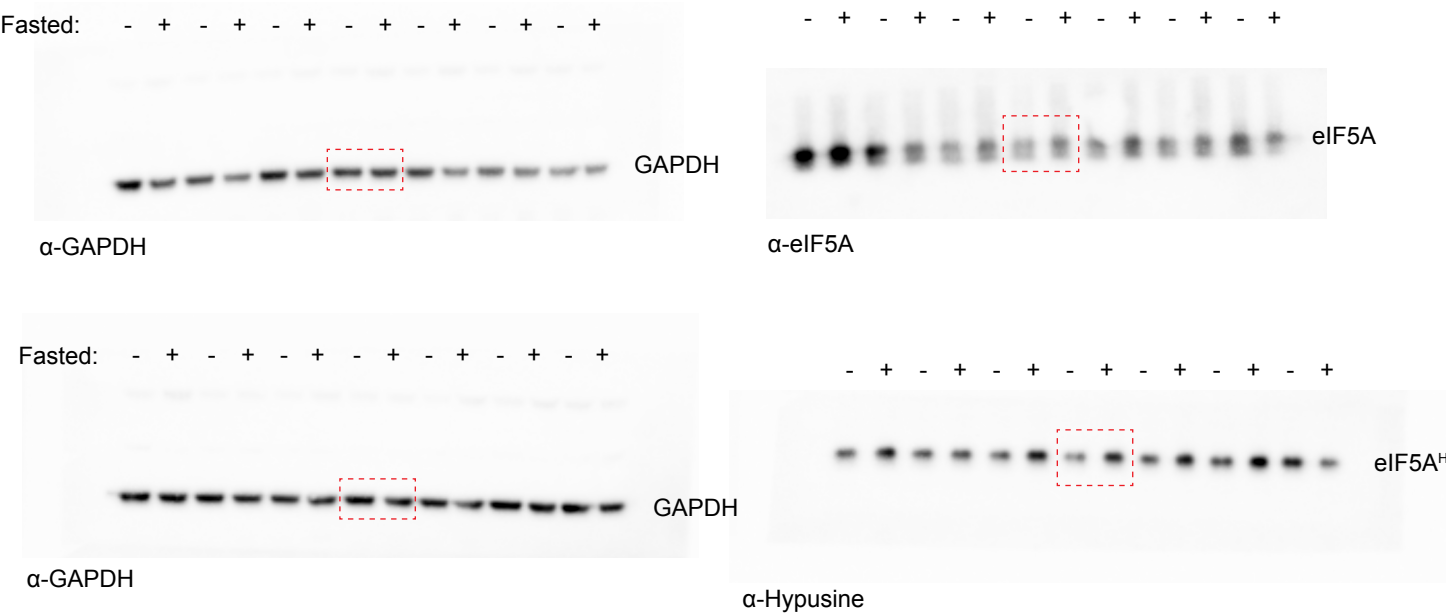

Figure 6K

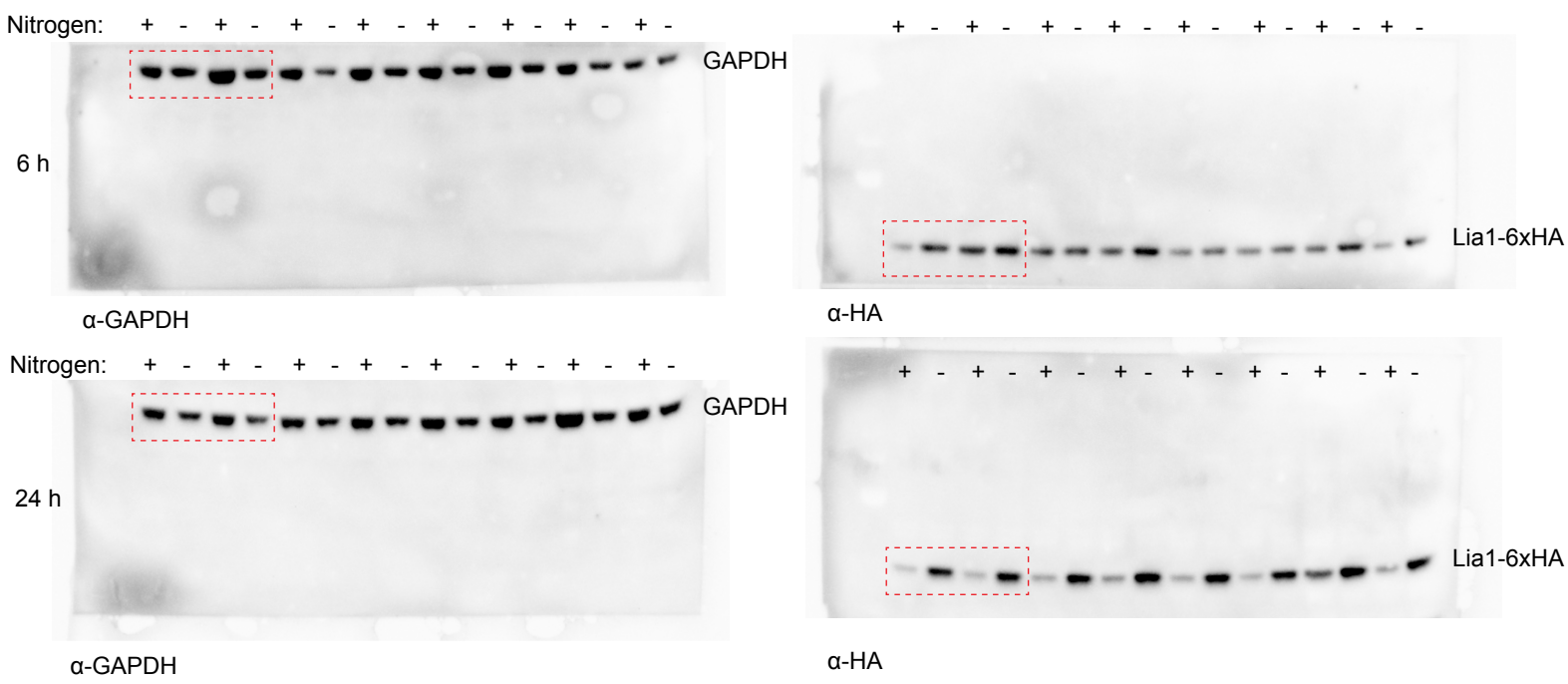

Supplement: Supplementary file 18 — Uncropped western blots. [file 41556_2024_1468_MOESM18_ESM.pdf]
