# Supplementary material for: Spermidine is essential for fasting-mediated autophagy and longevity
Source: Nat Cell Biol. 2024 Aug 8;26(9):1571–84. doi: 10.1038/s41556-024-01468-x (PMC11392816; doi:10.1038/s41556-024-01468-x)
Supplement: Supplementary file 23 — Uncropped western blots. [file 41556_2024_1468_MOESM23_ESM.pdf]

Extended Data Figure 4A

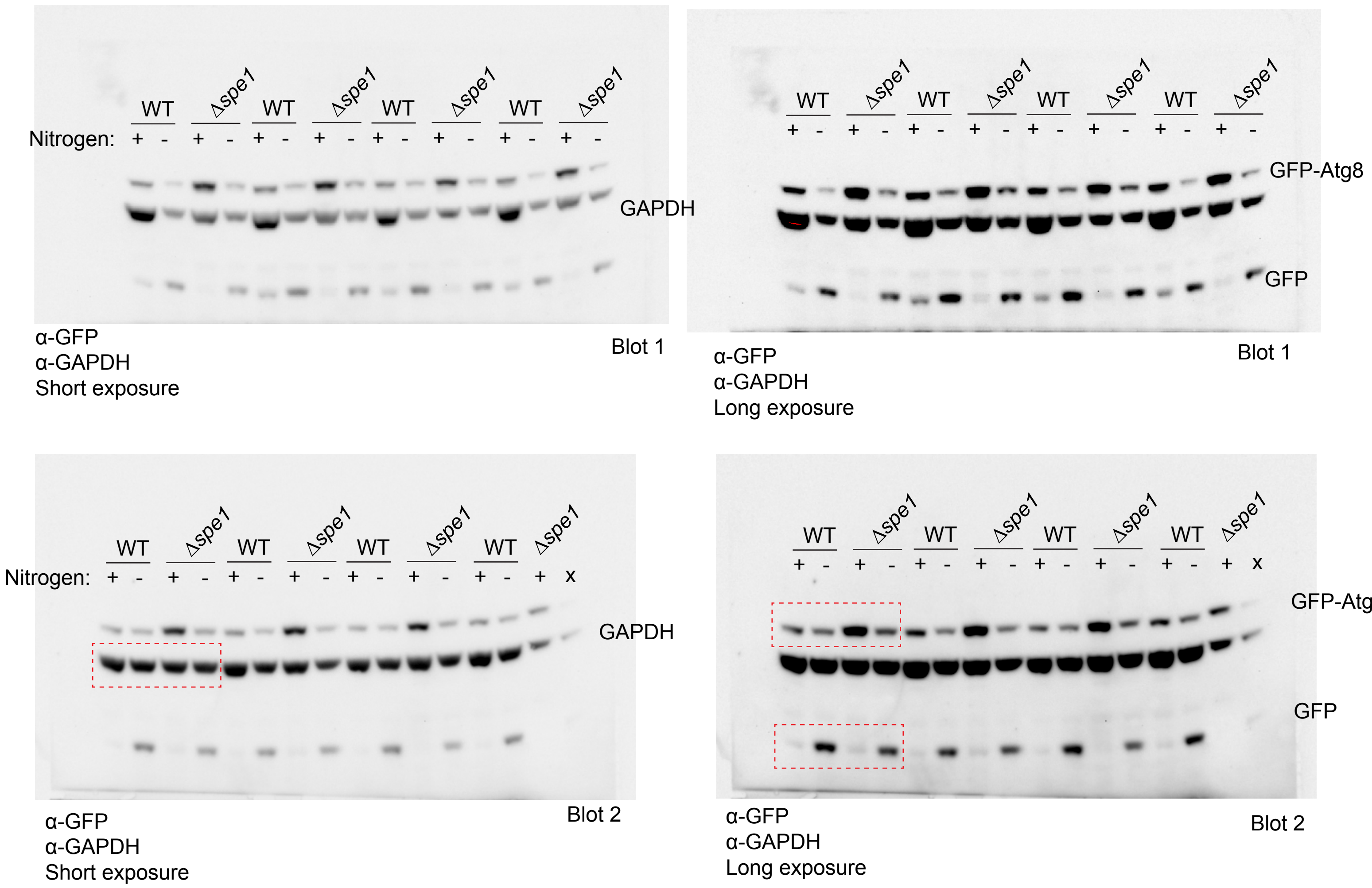

The lower parts of these blots were probed for α-hypusine in Fig. 6B and therefore share the α-GAPDH images.

Extended Data Figure 4C

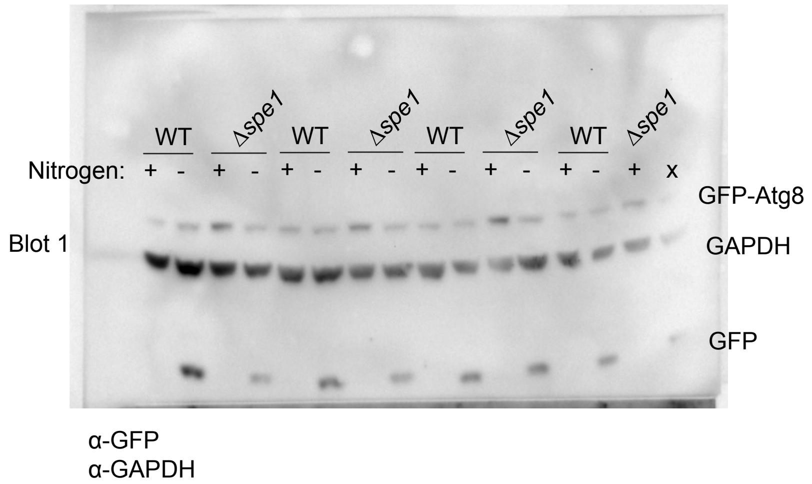

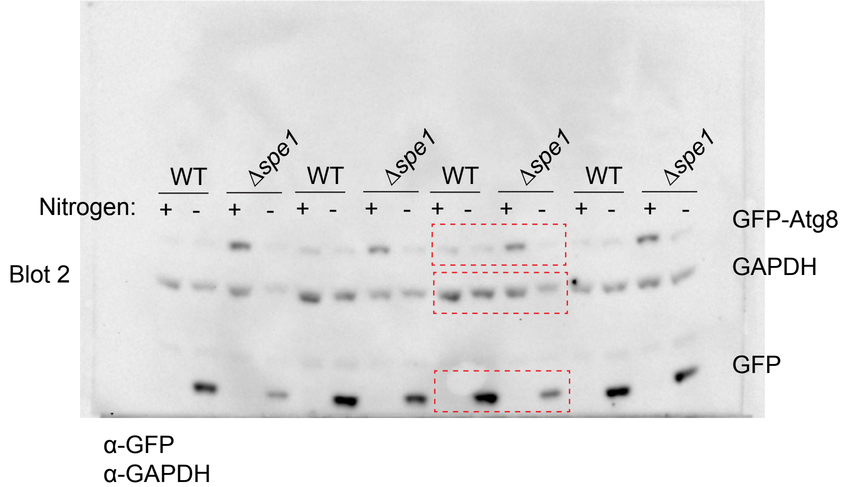

The lower parts of these blots were probed for α-hypusine in Extended Data Fig. 9A and therefore share the α-GAPDH images.

Extended Data Figure 4l

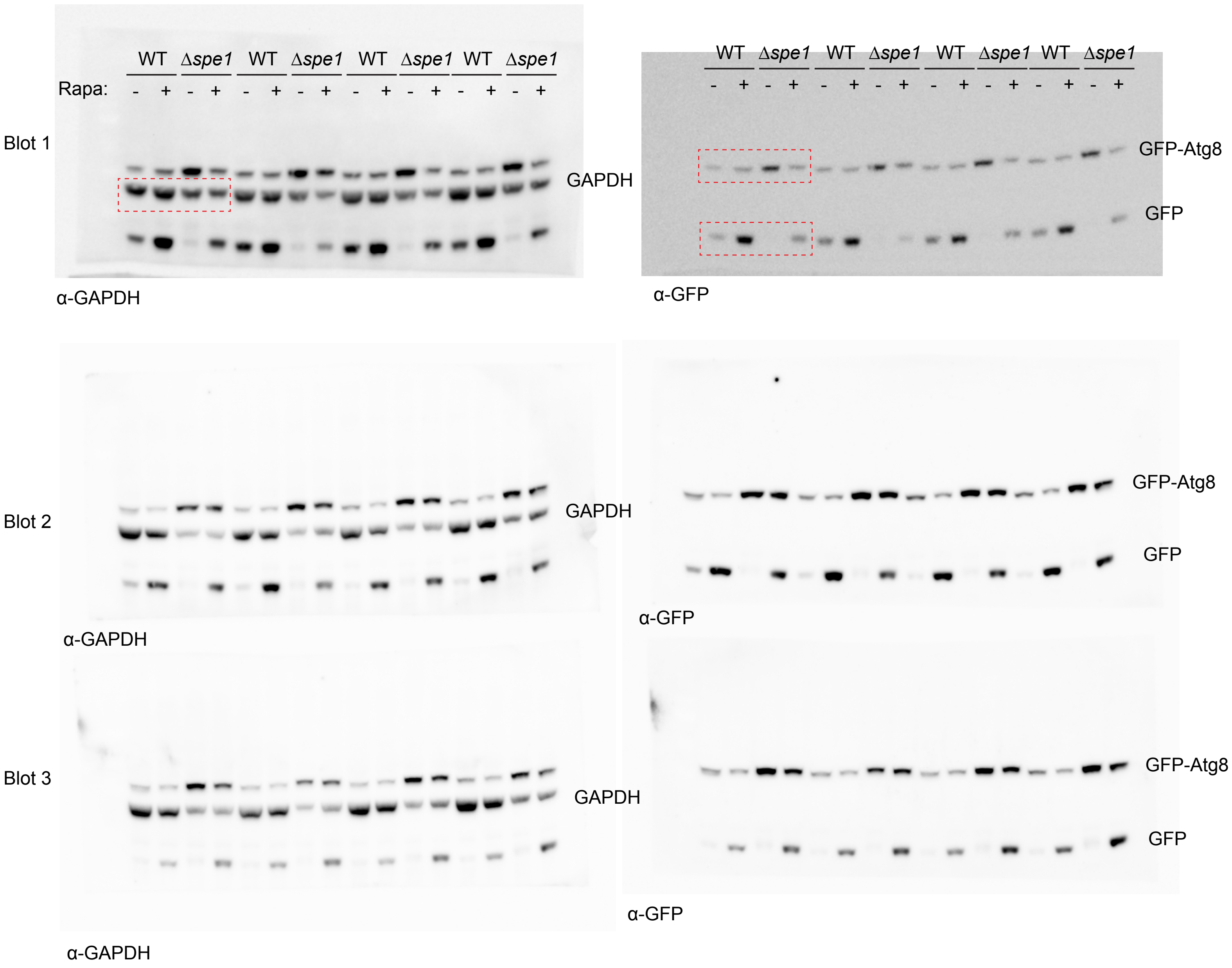

Extended Data Figure 4M

Blot 1

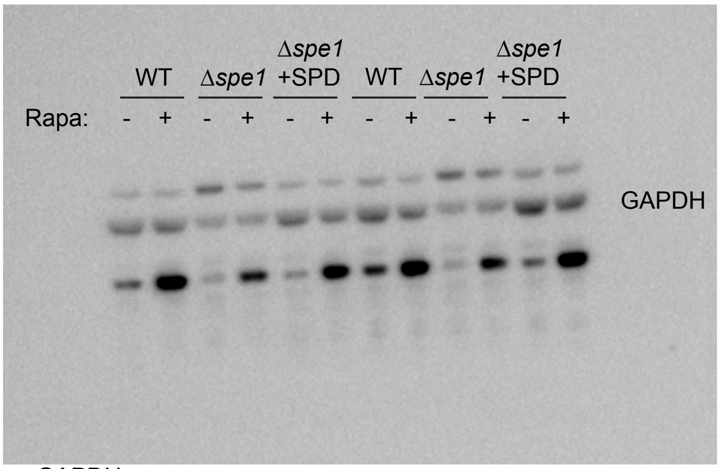

α-GAPDH

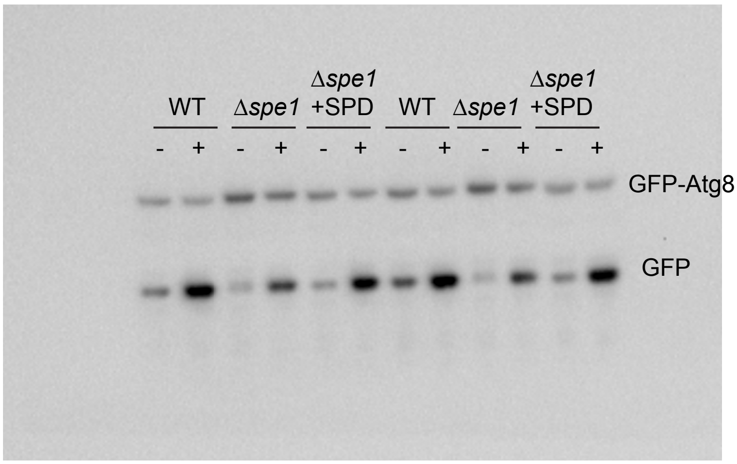

α-GFP

Blot 2

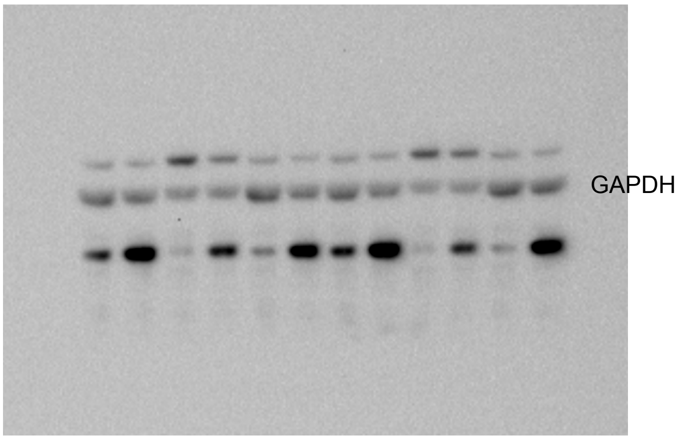

α-GAPDH

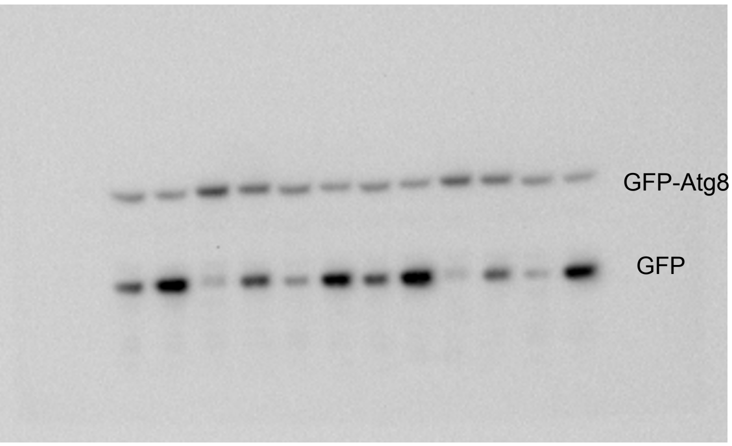

α-GFP

Blot 3

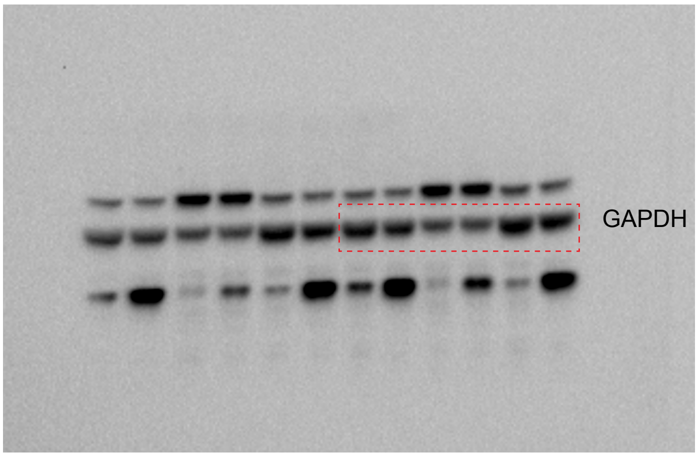

α-GAPDH

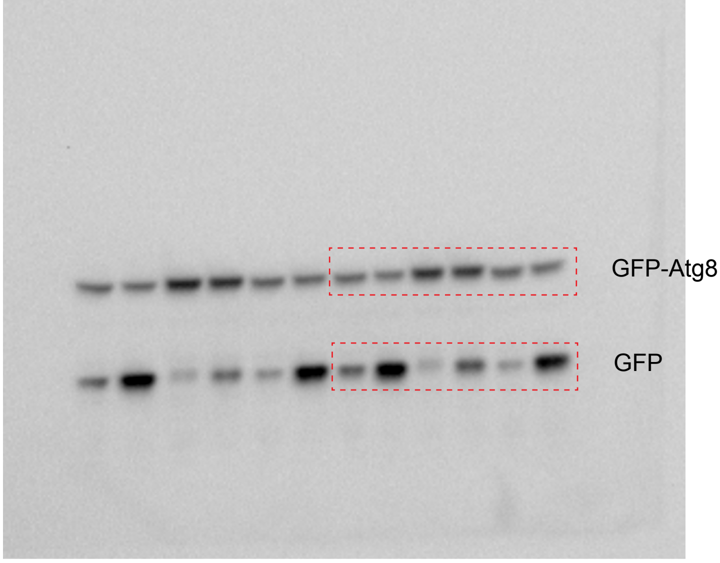

α-GFP
