# Supplementary material for: Spermidine is essential for fasting-mediated autophagy and longevity
Source: Nat Cell Biol. 2024 Aug 8;26(9):1571–84. doi: 10.1038/s41556-024-01468-x (PMC11392816; doi:10.1038/s41556-024-01468-x)
Supplement: Supplementary file 29 — Uncropped western blots. [file 41556_2024_1468_MOESM29_ESM.pdf]

Extended Data Figure 9A

Blot 1

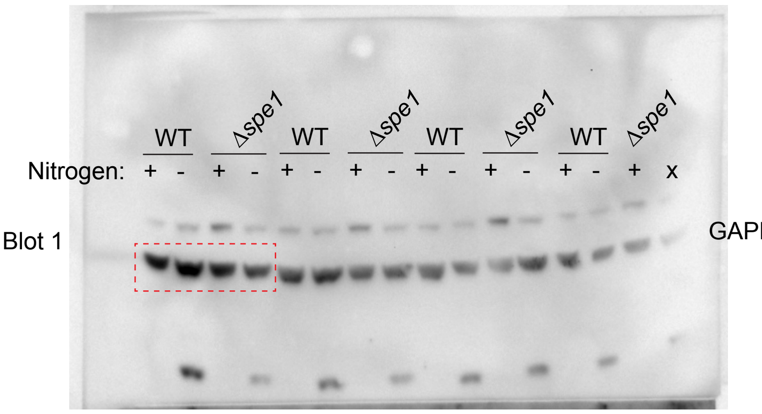

GAPDH

α-GAPDH

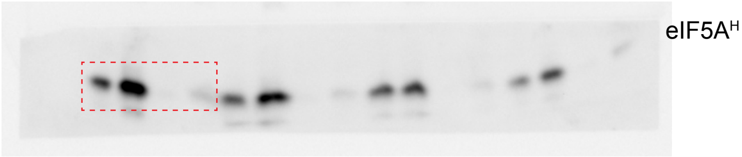

eIF5A^{H}

α-Hypusine

Blot 2

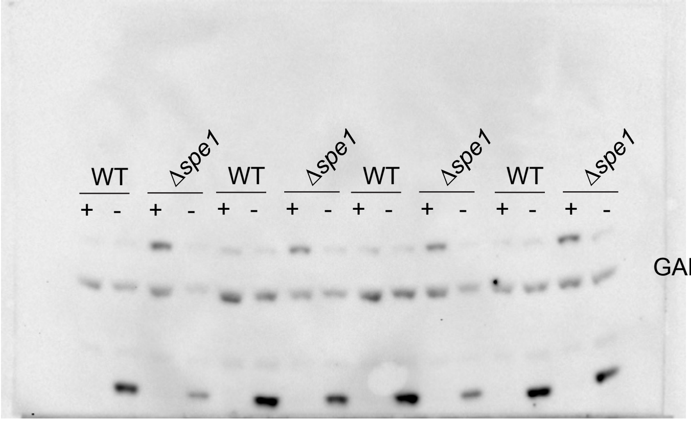

GAPDH

α-GAPDH

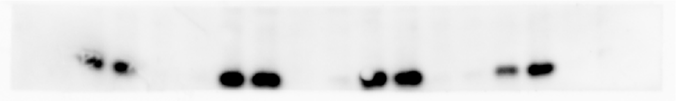

eIF5A^{H}

α-Hypusine

These blots were also probed for α-GFP in Extended Data Fig. 4C and therefore share the α-GAPDH images.

Extended Data Figure 9C

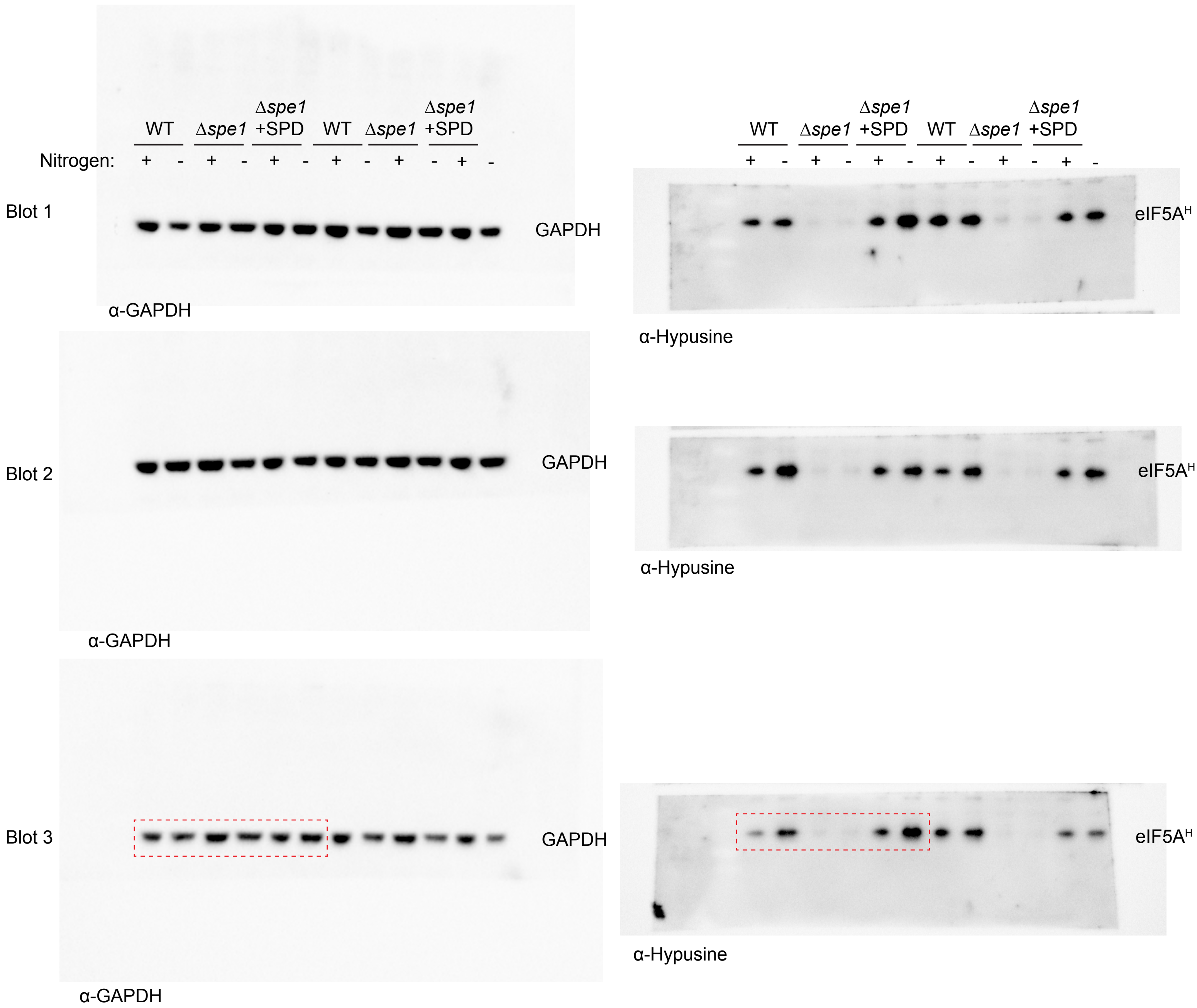

Extended Data Figure 9E

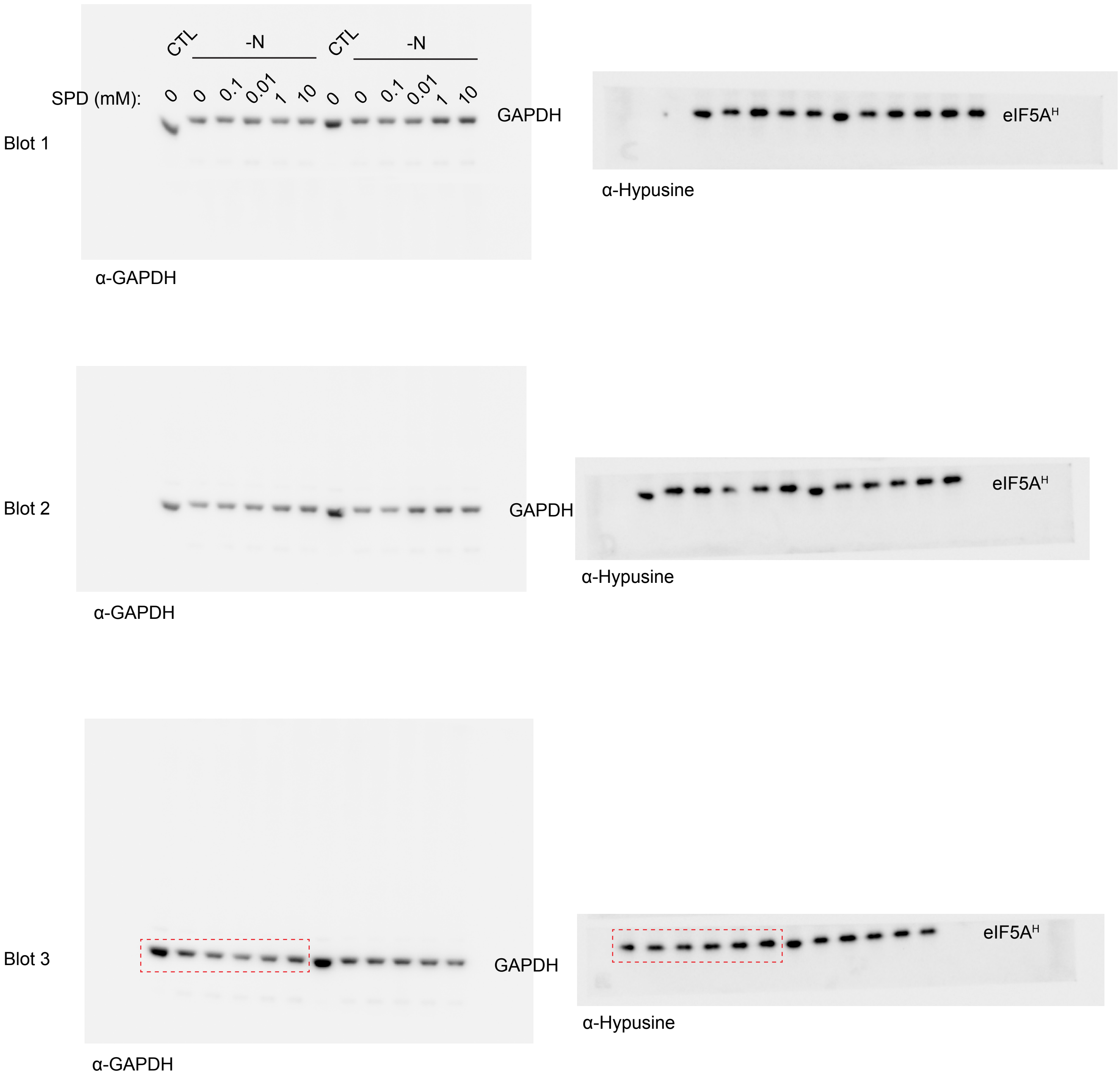

These blots were also probed for α-GFP in Fig. 3K and therefore share the α-GAPDH images.

Extended Data Figure 9I

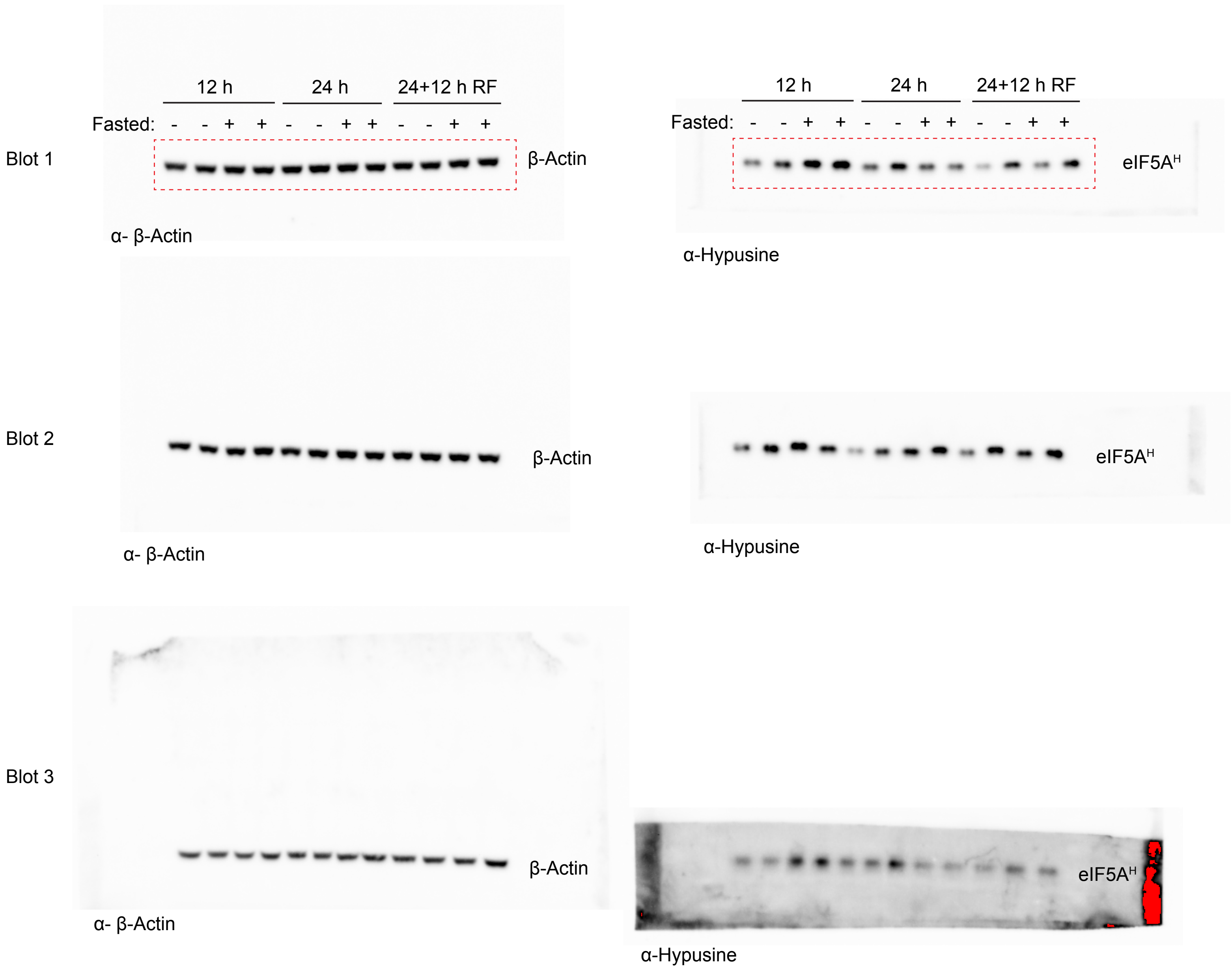

Extended Data Figure 9M

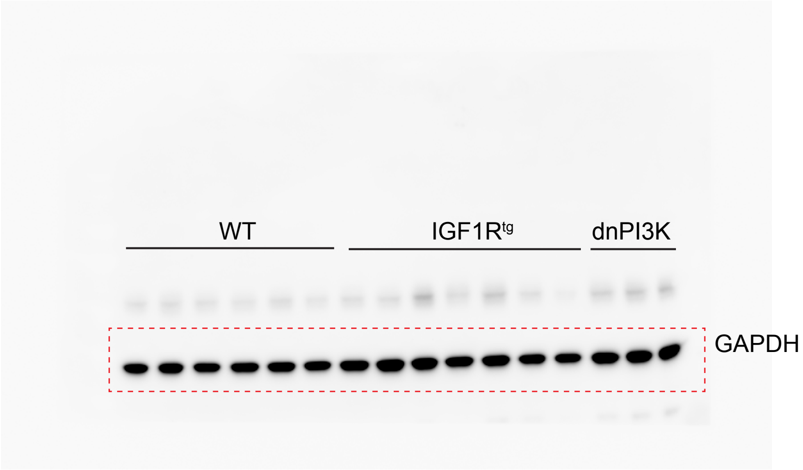

α-GAPDH

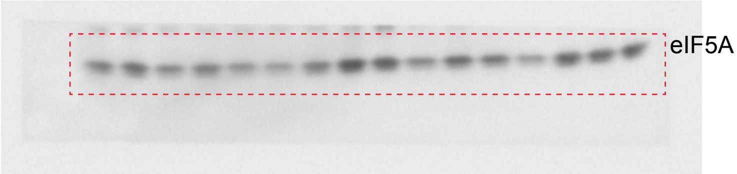

α-eIF5A

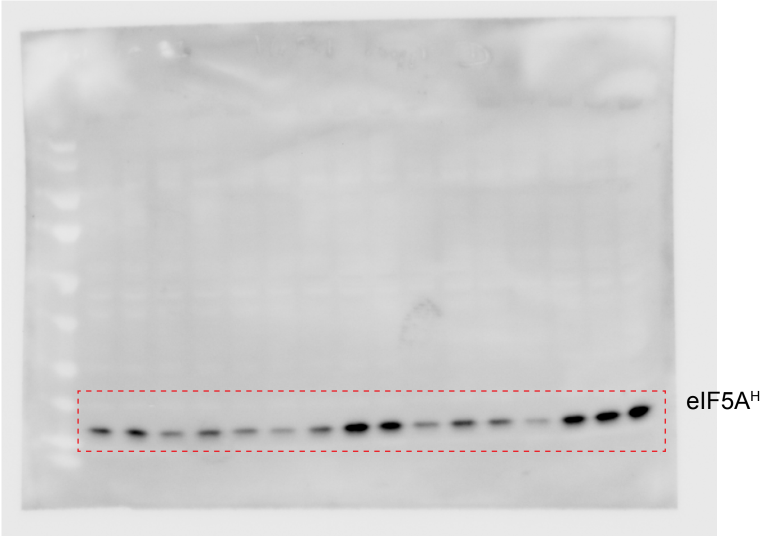

α-Hypusine

Extended Data Figure 9O

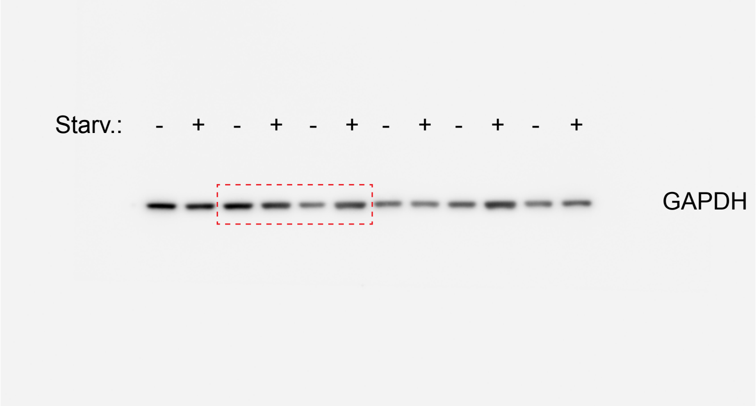

α-GAPDH

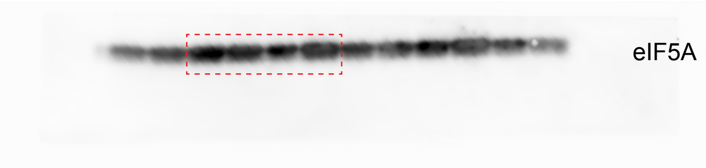

α-eIF5A

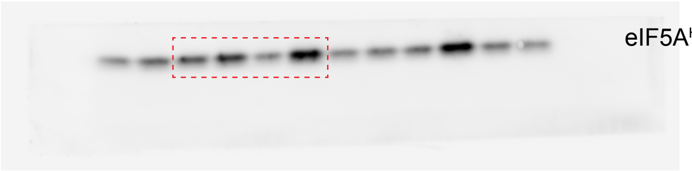

α-Hypusine
